# Supplementary material for: Experiences and challenges in accessing hospitalization in a government-funded health insurance scheme: Evidence from early implementation of Pradhan Mantri Jan Aarogya Yojana (PM-JAY) in India
Source: PLoS One. 2022 May 12;17(5):e0266798. doi: 10.1371/journal.pone.0266798 (PMC9098065; doi:10.1371/journal.pone.0266798)
Supplement: S1 File — (DOCX) [file pone.0266798.s001.docx]

**S1 File. Patient Survey Questionnaire**

| **Sr. No.** | **Question** | **Options** | **Response** |
| --- | --- | --- | --- |
|  | **ID details** | | |
|  | Enumerator’s Name |  | |
|  | Date of survey |  | |
|  | Patient’s name |  | |
|  | PMJAY ID | |  |
|  | QID | |  |
|  | State | 1. Gujarat 2. Madhya Pradesh |  |
|  | Name of hospital | **Confidential. Removed** | |
|  | Patient Location | 1. Same District, Urban 2. Same District, Non-Urban 3. Other District, Urban 4. Other District, Non-Urban |  |
|  | Name of location (City, Town, Village) |  | |

|  | **Background information** | | |
| --- | --- | --- | --- |
|  | Sex of patient | 1. Female 2. Male 3. Other |  |
|  | Age of patient ( In completed years) |  |  |
|  | Religion of patient | 1. Hindu 2. Muslim 3. Christian 4. Sikh 5. Other (specify) 6. Refuse to answer |  |
|  | Caste of patient | 1. Scheduled Tribe 2. Scheduled Caste 3. OBC/SEBC 4. Others (General) 5. Refuse to answer |  |
|  | Highest level of education of patient | 1. Not literate 2. Literate, no formal education 3. 1 to 5 standard 4. 6 to 8 standard 5. 9 to 12 standard/ Diploma (post class X) 6. Graduates/ Diploma (post class XII) and above 7. Other (specify) |  |
|  | Household size^^[[1]](#footnote-1)^^ | |  |
|  | Type of dwelling | 1. Kuccha 2. Pucca 3. Semi pucca |  |
|  | Main source of household income | 1. Farm Labour 2. Other labour work in rural area 3. Labour work in urban area 4. Self-employment (agricultural work) 5. Self-employment (other than agricultural work) 6. Salaried job 7. Others (Specify) | |
|  | Approximate monthly income (Rs.) |  | |

|  | **Experience with government sponsored health insurance scheme (GSHIS) before PMJAY** | | |
| --- | --- | --- | --- |
|  | Was the patient enrolled in any existing GSHIS before PMJAY? | 1. Yes 2. No (Skip to section 4) |  |
|  | Name of the scheme | 1. Rajya Bimari Sahayta Yojana 2. Bhopal Gas Victims Scheme 3. Deen Dayal Antyodaya Upchar Yojna 4. CM/PM Fund 5. RSBY 6. MA/MA Vatsalya 7. Other (Specify) |  |
|  | Ever availed hospitalization benefits under this coverage? | 1. Yes 2. No (Skip to 4) |  |
|  | Pl provide details of last such hospitalization_ reason for hospitalization |  | |
|  | Pl provide details of last such hospitalization_ details of care/services provided | 1. Hospitalization with surgery 2. Hospitalization without surgery 3. Day care procedures (Dialysis, BT, Chemo) 4. Others (Specify) | |
|  | Pl provide details of last such hospitalization_ type of hospital | 1. Public 2. Private 3. NGO | |
|  | Pl provide details of any additional payments made for this hospitalization | 1. Amount ______________________ 2. Reason for payment **_____________** | |
|  | How do you rate overall experience of treatment and coverage for that episode?    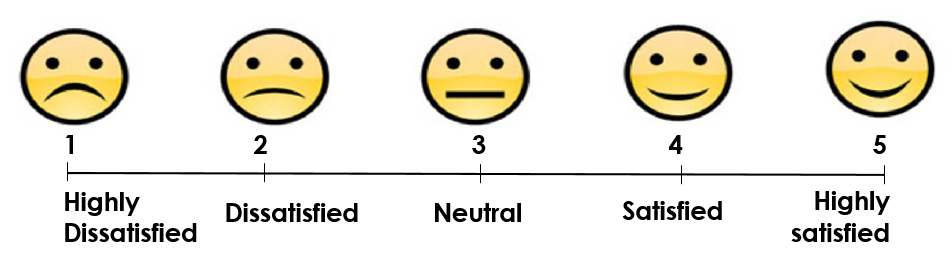 | | |

|  | **Experience of registration in PMJAY** | | | |
| --- | --- | --- | --- | --- |
|  | How did you come to know about PMJAY? (Multiple options are possible) | | 1. Received letter from government 2. Newspaper advertisement 3. From hospital at the time of admission 4. Internet 5. Other (Pl specify) |  |
|  | How did you check your eligibility? | | 1. Self-check through Am I Eligible Portal 2. Ayushman Bharat PM-JAY Mobile App 3. Calling the National Call Centre 4. Visiting the nearest Common Service Centre 5. Visiting nearest empanelled hospital 6. From hospital at the time of admission 7. Letter from Government |  |
|  | Where did you get registered under PMJAY | 1. Empanelled hospital 2. Common Service Centre | |  |
|  | How did you come to know about the list of empaneled hospitals/name of the hospital where you received treatment? | 1. Ayushman Bharat PM-JAY Mobile App 2. Calling the National Call Centre 3. Advertisement of the hospital 4. Explored at the hospital 5. From Relatives and friends 6. From Healthcare providers 7. Other (specify) | |  |
|  | What documents were required when you got registered in PMJAY? (Multiple answers are possible) | 1. Letter from government with PMJAY ID 2. Existing GSHIS ID card 3. Aadhar or ration card 4. Other (specify) | |  |
|  | Do you know about a staff called Ayush Mitra at the hospital? | 1. Yes 2. No | | |
|  | What helps did you receive at registration desk? (Multiple answers are possible) | 1. Provided information about PMJAY 2. Helped with documents and computerized registration 3. Guided about treatment within the hospital 4. Other (specify) | | |
|  | Did you face any difficulties in registration? Pl elaborate |  | | |
|  | Did you pay to anyone at the hospital for anything during registration? | 1. Yes 2. No (Skip to section 4.12) | | |
|  | Pl specify the amount (in Rs.) |  | | |
|  | Whom did you pay? | 1. Hospital Billing counter 2. Ayushman Mitra or anyone at Reg. Desk 3. Other (Specify) | |  |
|  | How do you rate overall experience of registration?  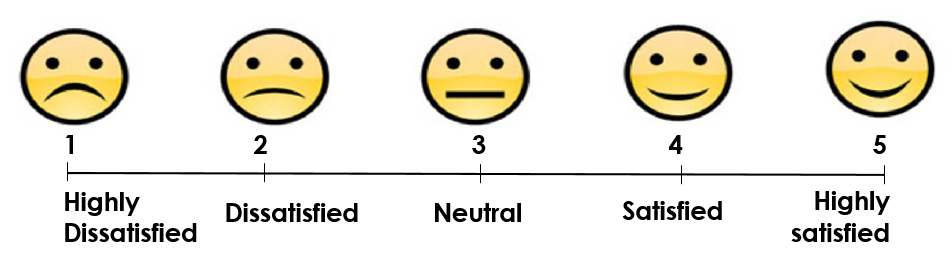 | | | |

|  | **Experience of hospitalization** | | |
| --- | --- | --- | --- |
|  | Note: We know you received treatment from ________ hospital recently. We would like to know more about your recent hospitalization. | | |
|  | Pl provide reason for hospitalization |  | |
|  | Pl provide details of care/services provided | 1. Hospitalization with surgery 2. Hospitalization without surgery 3. Day care procedures (Dialysis, BT, Chemo) 4. Others (Specify) | |
|  | For how many days the patient was admitted at the hospital? |  | |
|  | Did you face any difficulties in during hospitalization? Pl elaborate |  | |
|  | The PMJAY provides for the expenses of pre-hospitalization expenses for tests and medicines. Did you pay to anyone at the hospital for anything ***before*** getting admitted **that was not reimbursed to you**? | 1. Yes 2. No (Skip to 5.11) |  |
|  | Whom did you pay? | 1. Ayushman Mitra 2. Doctor 3. Nurse/Ward boy 4. Pharmacist/Technician 5. Hospital counter 6. Other (specify) |  |
|  | How much did you pay (In Rs.)? For what? | 1. Clinical services^^[[2]](#footnote-2)^^ |  |
|  |  | 1. Non-clinical services^^[[3]](#footnote-3)^^ |  |
|  |  | 1. Total |  |
|  | What was the reason why you were asked to make this payment? Pl elaborate |  | |
|  | The PMJAY provides for free treatment for hospitalization. Did you pay to anyone at the hospital for anything ***while the patient was admitted***? | 1. Yes 2. No (Skip to 5.15) |  |
|  | Whom did you pay? | 1. Ayushman Mitra 2. Doctor 3. Nurse/Ward boy 4. Pharmacist/Technician 5. Hospital counter 6. Other (specify) |  |
|  | How much did you pay (In Rs.)? For what? | 1. Clinical services^^[[4]](#footnote-4)^^ |  |
|  |  | 1. Non-clinical services^^[[5]](#footnote-5)^^ |  |
|  |  | 1. Total |  |
|  | What was the reason why you were asked to make this payment? Pl elaborate |  | |
|  | The PMJAY provides for post-hospitalization expenses of medicines up to 15 days. Did you pay to anyone at the hospital for anything ***within 15 days of discharge of the patient***? | 1. Yes 2. No (Skip to 5.19) |  |
|  | Whom did you pay? | 1. Ayushman Mitra 2. Doctor 3. Nurse/Ward boy 4. Pharmacist/Technician 5. Hospital counter 6. Other (specify) |  |
|  | How much did you pay (In Rs.)? For what? | 1. Clinical services^^[[6]](#footnote-6)^^ |  |
|  |  | 1. Non-clinical services^^[[7]](#footnote-7)^^ |  |
|  |  | 1. Total |  |
|  | What was the reason why you were asked to make this payment? Pl elaborate |  | |
|  | How do you rate overall experience of treatment coverage under PMJAY for this episode of illness?  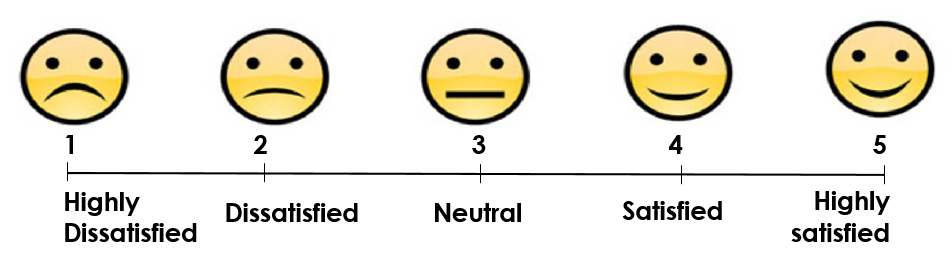 | | |

|  | **Time implications at each contact points / Touch points** | |
| --- | --- | --- |
|  | Following are critical touch-points for beneficiaries in PMJAY. We would like to know how much time (In minutes) did the patient/ relatives spent at each touch points.  If patient doesn’t remember, code the response as 9999. | |
|  | Beneficiary Verification |  |
|  | Hospitalization : Admission and Pre-authorization request |  |
|  | Hospitalization : Pre-authorization approval |  |
|  | Hospitalization : Discharge from hospital |  |

|  | **Information exchange in PMJAY** | |
| --- | --- | --- |
|  | PMJAY has an integrated feedback system for communication with patients with the help of calls and SMSs for various processes. We would like to know if patient/relative has received such information, from time to time, at below mentioned stages | 1. Call 2. SMS 3. Nothing |
|  | Beneficiary Verification _Silver record i.e. Verification in progress | 1. Call 2. SMS 3. Nothing |
|  | Beneficiary Verification _Golden record i.e. Details of scheme and coverage | 1. Call 2. SMS 3. Nothing |
|  | Hospitalization: Admission and Pre-authorization request i.e. Details of admission, packages and toll free no. | 1. Call 2. SMS 3. Nothing |
|  | Hospitalization : Pre-authorization approval i.e. Claims amount and details of financial implications, if any | 1. Call 2. SMS 3. Nothing |
|  | Hospitalization : Discharge from hospital, payment, balance, details of post-hospitalization, and financial implications, if any | 1. Call 2. SMS 3. Nothing |
|  | Post Discharge Feedback | 1. Call 2. SMS 3. Nothing |

|  | **Any other feedback / inputs with reference to PMJAY** |
| --- | --- |
|  |  |

1. Include all members who eat and sleep within the premises of the household for at least 10 days of the month [↑](#footnote-ref-1)
2. This will include payments related to hospitalization viz. diagnosis, consultation, medicine, etc. [↑](#footnote-ref-2)
3. This will include informal payments made to any of the service providers [↑](#footnote-ref-3)
4. This will include payments related to hospitalization viz. diagnosis, consultation, medicine, etc. [↑](#footnote-ref-4)
5. This will include informal payments made to any of the service providers [↑](#footnote-ref-5)
6. This will include payments related to hospitalization viz. diagnosis, consultation, medicine, etc. [↑](#footnote-ref-6)
7. This will include informal payments made to any of the service providers [↑](#footnote-ref-7)
