# Supplementary material for: Experiences and challenges in accessing hospitalization in a government-funded health insurance scheme: Evidence from early implementation of Pradhan Mantri Jan Aarogya Yojana (PM-JAY) in India
Source: PLoS One. 2022 May 12;17(5):e0266798. doi: 10.1371/journal.pone.0266798 (PMC9098065; doi:10.1371/journal.pone.0266798)
Supplement: S1 Table — (DOCX) [file pone.0266798.s002.docx]

| State | | Gujarat | Madhya Pradesh |
| --- | --- | --- | --- |
| Gross State Domestic Product (GSDP) per capita (INR)* | | 224896 | 99025 |
| State health expenditure (2019-20 Budget Estimate)* | Per capita (INR) | 1610 | 1284 |
|  | Percent of GSDP | 0.7 | 1.3 |
|  | Percent of total revenue expenditure | 7.1 | 5.9 |
| Per capita health expenditure (INR) (2016-17)** | Total | 3703 | 2820 |
|  | Government | 1429 | 811 |
|  | OOP | 1781 | 1944 |
| Average total expenditure per hospitalization case (excluding child birth) (INR) (2017-18)*** | Private hospital – Rural | 25027 | 25086 |
|  | Private hospital – Urban | 29281 | 31094 |
|  | Charitable hospital - Rural | 16585 | 34643 |
|  | Charitable hospital - Urban | 12917 | 26516 |
|  | Public hospital – Rural | 1151 | 2093 |
|  | Public hospital – Urban | 3529 | 2030 |
| Average out-of-pocket medical expenditure (OOPME) per hospitalization case (excluding child birth) (INR) (2017-18)*** | Private hospital – Rural | 23656 | 24471 |
|  | Private hospital – Urban | 21746 | 28603 |
|  | Charitable hospital - Rural | 15543 | 34643 |
|  | Charitable hospital - Urban | 10165 | 25741 |
|  | Public hospital – Rural | 1151 | 2093 |
|  | Public hospital – Urban | 3459 | 1889 |
| Sources: * State Finances: A Study of Budgets of 2020-21, Reserve Bank of India [[26](#_ENREF_26)]; ** National Health Accounts India (2016-17) [[27](#_ENREF_27)] *** National Sample Survey Organization [[29](#_ENREF_29)] | | | |

**S1 Table. Comparison of study states across selected expenditure parameters**
