# Supplementary material for: Experiences and challenges in accessing hospitalization in a government-funded health insurance scheme: Evidence from early implementation of Pradhan Mantri Jan Aarogya Yojana (PM-JAY) in India
Source: PLoS One. 2022 May 12;17(5):e0266798. doi: 10.1371/journal.pone.0266798 (PMC9098065; doi:10.1371/journal.pone.0266798)

PLOS ONE

# Improving Responsiveness and Financial Protection in Government-Funded Health Insurance Scheme in India: Evidence from Early Implementation of Pradhan Mantri Jan Aarogya Yojana (AB – PMJAY)

--Manuscript Draft--

| | |
|---|---|
| **Manuscript Number:** | PONE-D-21-06856 |
| **Article Type:** | Research Article |
| **Full Title:** | Improving Responsiveness and Financial Protection in Government-Funded Health Insurance Scheme in India: Evidence from Early Implementation of Pradhan Mantri Jan Aarogya Yojana (AB – PMJAY) |
| **Short Title:** | Responsiveness and Financial Protection in PMJAY |
| **Corresponding Author:** | Mayur Trivedi<br>Indian Institute of Public Health Gandhinagar<br>Gandhinagar, Gujarat INDIA |
| **Keywords:** | Health System Responsiveness; PM-JAY; India; Financial Protection; Universal Health Coverage; Ayushman Bharat; Health Insurance |
| **Abstract:** | Introduction<br><br>Improvement in health outcomes, responsiveness and financial protection remain priorities of the health system, in general, and health insurance system, in particular. This research work aimed to understand the hospitalization experiences of beneficiaries of Pradhan Mantri Jan Aarogya Yojana (PM-JAY), a Government-sponsored health insurance scheme in India. It focused on identifying the enablers and barriers of healthcare utilization, perceived satisfaction of patients, and their access to information for successful implementation of the scheme.<br><br>Method<br><br>Data for this study were obtained through a household survey of 200 beneficiaries from Gujarat and Madhya Pradesh (MP) was conducted. The study population included patients, who availed services from the 14 study hospitals in April 2019. Data collection was done using a structured questionnaire. The study was carried out from March 2019 to August 2019.<br><br>Results<br><br>This research provided evidence of experiences of the beneficiary's interaction with providers, processes, institutions, and information is central to the responsiveness of PM-JAY. It indicated that the patients are satisfied with the implementation of PM-JAY scheme, in general. It also provided evidence on mean out of pocket expense of Rs. 27648 during hospitalization during hospitalization. This influences financial and physical coverage and ultimately affects user satisfaction. With few patients indicating the receipt of SMSs and calls for various hospital-based transactions, the findings indicates the need for improvement in the provision of communication with patients for better responsiveness.<br><br>Conclusion<br><br>This research provides insights from the beneficiaries based on their experience of hospitalization under PM-JAY and emphasized the inter-connected issues of responsiveness and financial protection in the health insurance system. Among the very few research on the responsiveness of GSHIS schemes, this research provides valuable evidence and offers avenues of improvement in the implementation of PM-JAY. |
| **Order of Authors:** | Mayur Trivedi |
| | Anurag Saxena |
| | |

Powered by Editorial Manager® and ProduXion Manager® from Aries Systems Corporation

| | |
|---|---|
| | Zubin Shroff |
| | Manas Sharma |
| **Opposed Reviewers:** | |
| **Additional Information:** | |
| **Question** | **Response** |
| **Financial Disclosure**<br><br>Enter a financial disclosure statement that describes the sources of funding for the work included in this submission. Review the submission guidelines for detailed requirements. View published research articles from *PLOS ONE* for specific examples.<br><br>This statement is required for submission and **will appear in the published article** if the submission is accepted. Please make sure it is accurate.<br><br>**Unfunded studies**<br>Enter: *The author(s) received no specific funding for this work.*<br><br>**Funded studies**<br>Enter a statement with the following details:<br>• Initials of the authors who received each award<br>• Grant numbers awarded to each author<br>• The full name of each funder<br>• URL of each funder website<br>• Did the sponsors or funders play any role in the study design, data collection and analysis, decision to publish, or preparation of the manuscript?<br>• **NO** - Include this sentence at the end of your statement: *The funders had no role in study design, data collection and analysis, decision to publish, or preparation of the manuscript.*<br>• **YES** - Specify the role(s) played.<br><br>* typeset | The research was made possible through the financial support provided by the Alliance for Health Policy and Systems Research, World Health Organization vide PO Purchase Order 202214458 for research entited as Health Systems Research for PM-JAY Improving hospital-based processes for effective implementation. The grant period was 4<br>March to 3 September 2019. The URL of the funder is www.who.int. Dr Zubin Shroff of WHO provided guidance in strengthening the methodology and finalizing the instruments, and reviewed the manuscript critically. |
| **Competing Interests**<br><br>Use the instructions below to enter a competing interest statement for this submission. On behalf of all authors, disclose any competing interests that could be perceived to bias this | The authors have declared that no competing interests exist. |

| | |
|---|---|
| work—acknowledging all financial support and any other relevant financial or non-financial competing interests.<br><br>This statement is required for submission and **will appear in the published article** if the submission is accepted. Please make sure it is accurate and that any funding sources listed in your Funding Information later in the submission form are also declared in your Financial Disclosure statement.<br><br>View published research articles from *PLOS ONE* for specific examples.<br><br>**NO authors have competing interests**<br><br>Enter: *The authors have declared that no competing interests exist.*<br><br>**Authors with competing interests**<br><br>Enter competing interest details beginning with this statement:<br><br>*I have read the journal's policy and the authors of this manuscript have the following competing interests: [insert competing interests here]*<br><br>* typeset | |
| **Ethics Statement**<br><br>Enter an ethics statement for this submission. This statement is required if the study involved:<br><br>• Human participants<br>• Human specimens or tissue<br>• Vertebrate animals or cephalopods<br>• Vertebrate embryos or tissues<br>• Field research<br><br>Write "N/A" if the submission does not require an ethics statement. | The research was approved by the Institutional Ethics Committee at Indian Institute of Public Health Gandhinagar, India via letter TRC-IEC No: 14/2018-19. A verbal informed consent was ensured from all the respondents before beginning of the survey. The data was analyzed anonymously. |

Powered by Editorial Manager® and ProduXion Manager® from Aries Systems Corporation

| | |
|---|---|
| General guidance is provided below. Consult the submission guidelines for detailed instructions. **Make sure that all information entered here is included in the Methods section of the manuscript.**<br><br>**Format for specific study types**<br><br>**Human Subject Research (involving human participants and/or tissue)**<br>• Give the name of the institutional review board or ethics committee that approved the study<br>• Include the approval number and/or a statement indicating approval of this research<br>• Indicate the form of consent obtained (written/oral) or the reason that consent was not obtained (e.g. the data were analyzed anonymously)<br><br>**Animal Research (involving vertebrate animals, embryos or tissues)**<br>• Provide the name of the Institutional Animal Care and Use Committee (IACUC) or other relevant ethics board that reviewed the study protocol, and indicate whether they approved this research or granted a formal waiver of ethical approval<br>• Include an approval number if one was obtained<br>• If the study involved *non-human primates*, add *additional details* about animal welfare and steps taken to ameliorate suffering<br>• If anesthesia, euthanasia, or any kind of animal sacrifice is part of the study, include briefly which substances and/or methods were applied<br><br>**Field Research**<br><br>Include the following details if this study involves the collection of plant, animal, or other materials from a natural setting:<br>• Field permit number<br>• Name of the institution or relevant body that granted permission | |
| **Data Availability**<br><br>Authors are required to make all data underlying the findings described fully | No - some restrictions will apply |

| | |
|---|---|
| available, without restriction, and from the time of publication. PLOS allows rare exceptions to address legal and ethical concerns. See the PLOS Data Policy and FAQ for detailed information.<br><br>A Data Availability Statement describing where the data can be found is required at submission. Your answers to this question constitute the Data Availability Statement and **will be published in the article**, if accepted.<br><br>**Important:** Stating 'data available on request from the author' is not sufficient. If your data are only available upon request, select 'No' for the first question and explain your exceptional situation in the text box.<br><br>Do the authors confirm that all data underlying the findings described in their manuscript are fully available without restriction? | |
| **Describe where the data may be found in full sentences. If you are copying our sample text, replace any instances of XXX with the appropriate details.**<br><br>• If the data are **held or will be held in a public repository**, include URLs, accession numbers or DOIs. If this information will only be available after acceptance, indicate this by ticking the box below. For example: *All XXX files are available from the XXX database (accession number(s) XXX, XXX.*).<br>• If the data are all contained **within the manuscript and/or Supporting Information files**, enter the following: *All relevant data are within the manuscript and its Supporting Information files.*<br>• If neither of these applies but you are able to provide **details of access elsewhere**, with or without limitations, please do so. For example:<br><br>*Data cannot be shared publicly because of [XXX]. Data are available from the XXX Institutional Data Access / Ethics* | The datasets used and/or analysed during the current study are available from the corresponding author on reasonable request. |

| | |
|---|---|
| *Committee (contact via XXX) for researchers who meet the criteria for access to confidential data.*<br><br>*The data underlying the results presented in the study are available from (include the name of the third party and contact information or URL).*<br>• This text is appropriate if the data are owned by a third party and authors do not have permission to share the data.<br><br>* typeset | |
| Additional data availability information: | Tick here if your circumstances are not covered by the questions above and you need the journal’s help to make your data available. |

Powered by Editorial Manager® and ProduXion Manager® from Aries Systems Corporation

To,

Emily Chenette

Editor-in-chief, PLOS One

Subject: Manuscript submission

Dear Emily

Greetings!

I am submitting a manuscript titled *Improving Responsiveness and Financial Protection in Government-Funded Health Insurance Scheme in India: Evidence from Early Implementation of Pradhan Mantri Jan Aarogya Yojana (AB – PMJAY)*" in PLOS One. This 'research article' is submitted as a response to the calls for papers for Health Services Research.

The manuscript examines the hospitalization experiences of the beneficiaries of Ayushman Bharat Pradhan Mantri Jan Arogya Yojana (PM-JAY), a Government-sponsored health insurance scheme in India. It focused on identifying the enablers and barriers of healthcare utilization, perceived satisfaction of patients, and their access to information for successful implementation of the scheme. The results emphasized the inter-connected issues of responsiveness and financial protection in the health insurance system. The study connects to various existing literature from similar research on previous health insurance schemes. However, as one among the very few research on the responsiveness of GSHIS schemes, this research provides valuable evidence and offers avenues of improvement in the implementation of PM-JAY.

None of the authors of this manuscript have competing interests, all of the authors have approved the manuscript for submission, and the contents of this manuscript have not been submitted for publication elsewhere. A modified version of this manuscript is available as a working paper on https://pmjay.gov.in/sites/default/files/2020-02/WP_IIPH_study_2.pdf.

I look forward to encouraging feedback from your side.

Regards

Mayur Trivedi

Mauscript

Click here to access/download;Manuscript;PMJAY household survey paper for PLOS.docx

# Improving Responsiveness and Financial Protection in Government-Funded Health Insurance Scheme in India: Evidence from Early Implementation of Pradhan Mantri Jan Aarogya Yojana (AB – PMJAY)

## Responsiveness and Financial Protection in PMJAY

**Authors**: Mayur Trivedi[1 *], Anurag Saxena[1], Zubin Shroff [2], Manas Sharma[1]

[1] Indian Institute of Public Health Gandhinagar

[2] World Health Organization, Geneva

* Corresponding Author

Email:mtrivedi@iiphg.org (MT)

### DECLARATION

**Ethical Approval:** The research was approved by the Institutional Ethics Committee at Indian Institute of Public Health Gandhinagar, India via letter TRC-IEC No: 14/2018-19.

**Availability of data and materials**: The datasets used and/or analysed during the current study are available from the corresponding author on reasonable request.

**Competing interests:** The author(s) declare(s) that they have no competing interests

**Abstract**

**Introduction**: Improvement in health outcomes, responsiveness and financial protection remain priorities of the health system, in general, and health insurance system, in particular. This research work aimed to understand the hospitalization experiences of beneficiaries of Pradhan Mantri Jan Aarogya Yojana (PM-JAY), a Government-sponsored health insurance scheme in India. It focused on identifying the enablers and barriers of healthcare utilization, perceived satisfaction of patients, and their access to information for successful implementation of the scheme.

**Method**: Data for this study were obtained through a household survey of 200 beneficiaries from Gujarat and Madhya Pradesh (MP) was conducted. The study population included patients, who availed services from the 14 study hospitals in April 2019. Data collection was done using a structured questionnaire. The study was carried out from March 2019 to August 2019.

**Results**: This research provided evidence of experiences of the beneficiary's interaction with providers, processes, institutions, and information is central to the responsiveness of PM-JAY. It indicated that the patients are satisfied with the implementation of PM-JAY scheme, in general. It also provided evidence on mean out of pocket expense of Rs. 27648 during hospitalization during hospitalization. This influences financial and physical coverage and ultimately affects user satisfaction. With few patients indicating the receipt of SMSs and calls for various hospital-based transactions, the findings indicates the need for improvement in the provision of communication with patients for better responsiveness.

**Conclusion**: This research provides insights from the beneficiaries based on their experience of hospitalization under PM-JAY and emphasized the inter-connected issues of responsiveness and financial protection in the health insurance system. Among the very few research on the responsiveness of GSHIS schemes, this research provides valuable evidence and offers avenues of improvement in the implementation of PM-JAY.

**Keywords:** Health System Responsiveness, PM-JAY, India, Financial Protection, Universal Health Coverage, Ayushman Bharat, Health Insurance

# Introduction

An important goal of the health system of any country is to improve the health outcomes of its citizens. However, despite attaining better health outcomes, people may remain unhappy with the health system due either to high expenses of treatment or the lack of a responsive health system. To that end, the health systems across the world also strive to provide financial protection against the costs of ill health, and respond to people's expectations, while preserving, promoting, and improving their health. [1]

Health system responsiveness is defined as "the ability of the health system to meet the population's legitimate expectations regarding their interaction with the health system, apart from expectations for improvements in health or wealth". [2] The responsiveness is about the provision of physical and affective support during the treatment. It includes elements of respect for people and client orientation elements like confidentiality, prompt attention, choice of providers, quality of amenities, etc. Also, the flow and clarity of information between the health system and the patients form an important element of responsiveness owing largely to information asymmetries in healthcare. [3] While the information from the providers to the patients enables the latter to decide the course of action and appreciate the treatment, the feedback of patients forms important information for strengthening provision. In the context of government programs, feedback from users to services providers and the government has been documented to be a useful tool to inform policy reforms. [4-6] While access to information is an important aspect of healthcare provision in general, the information imbalance gains more importance in the resource-constrained setting with low penetration of insurance and low level of financial and insurance literacy. [7] This is because the insurance providers – as payers - would carry informed negotiation with the healthcare providers on behalf of ill-informed insured patients to reduce unnecessary and expensive healthcare. This

may ultimately lead to improved insurance literacy among the insured patients and their relatives. It is argued that households with higher financial and insurance literacy not only demand more insurance, they also tend to appreciate the terms and conditions of the contract. [8] While the discourse around health system responsiveness in terms of experience and expectations of patients are limited to healthcare provisions perspectives in the developed countries settings, certain studies explored it in context of social health insurance schemes in developing countries. [9-11]

Although not studied much, the responsiveness is essentially important in government sponsored health insurance schemes (GSHIS) that ensure the financial and physical coverage of healthcare service delivery through improved strategic purchasing of healthcare services on behalf of the citizens. [2, 12] The GSHIS have been advocated as a means to move towards Universal Health Coverage (UHC) envisioned in the form of the target 3.8 of the United Nation's Sustainable Development Goals (SDGs). [13] In the past two decades, many low and middle-income countries, including India, have introduced GSHIS of different scale and scopes. While many of these schemes are effective in improving access and utilization of secondary and tertiary healthcare services [14, 15], there has been evidence of out of pocket expenses (OOP) leading to dissatisfaction among the beneficiaries [16-19]. In addition to improving financial protections, it is important to address the responsiveness of GSHIS through better interactions of beneficiaries with providers, processes, and institutional arrangements. [20]

India had a history of state-level as well as pan-Indian GSHIS for a while now with the considerable expansion of coverage. [21] These efforts of more than a decade have culminated into the launch of a nationwide GSHIS i.e. Pradhan Mantri Jan Aarogya Yojana (PM-JAY). In the context, India needs to consider the enablers and barriers of healthcare utilization, perceived

satisfaction of patients, and their access to information for successful implementation of PM-JAY. [22]

## About PM-JAY

The PM-JAY, the GSHIS launched by the Government of India in September 2018, aims to cover around 500 million beneficiaries from around 100 million poor and vulnerable families that form the bottom 40% of the Indian population. [23] The scheme is rolled out in active partnership with the states. While PMJAY eventually subsumed existing health insurance schemes in many states with a history of implementing GSHIS (for example, Mukhyamantri Amrutam scheme in Gujarat), it was launched as a new scheme in states where there was no pre-existing scheme (for example, Madhya Pradesh). The scheme provides coverage of up to INR 500,000 per family per year to cover the expenses for medicines and diagnostics for selected 1393 surgery, medical and daycare treatments at public hospitals and empanelled private hospitals. Some of these procedures, deemed prone to fraud and abuse, are reserved for public hospitals only. The expenses for these treatment/procedures are covered under PM-JAY only when it is availed in a public hospital, and the beneficiaries need to bear the expenses if the treatment is availed in a private hospital. [24]

PM-JAY involves certain touchpoints for beneficiaries' interaction with the health insurance system from the time a beneficiary arrives at the hospital for treatment to discharge. Accordingly, clearly defined guidelines have been developed for each step of the hospitalization process viz. a) registration, b) selection of treatment package before hospitalization, c) preauthorization and admission during hospitalization, d) discharge and payments, and e) information exchange at various touchpoints. [25]

The guidelines state that empanelled hospital need to ensure a dedicated helpdesk for PM-JAY at a prominent place in the hospital. The helpdesk is to be managed by the Ayushman Mitra

(AM), who manages the operations, and liaison with patients, doctors, hospital administration, and the scheme managers. The primary purpose of the helpdesk is to provide one-stop services regarding registration, hospitalization, and discharge. The PM-JAY guidelines state that based on the diagnosis sheet provided by the doctor, the AM/Computer operator should be able to block the treatment package(s) using PM-JAY IT system. Once the package is selected and blocked, a request is received by the insurance company/trust for pre-authorization. According to the guideline, the request has to be approved or rejected within six hours. According to the guidelines for discharge, that the AM/computer operator should be able to fill the online discharge summary form. Lastly, the patient should be provided with a discharge summary at the time of the discharge. It does not mention the time duration to complete this process. [26]

Rationale of the study

PM-JAY is expected to emerge as an effective mechanism for ensuring health coverage to a large number of poor Indians, who remain passive recipients of the healthcare care from the public and private sector. While responsiveness depends on a range of factors within the health system, the beneficiaries' expectations and experience of availing health coverage can also provide useful insights in the early phase of implementation. However, there remains a gap in knowledge about the experiences of beneficiary interactions in navigating these hospitalization processes, and in receipt of the timely and useful information. Insights from early implementation about patients' experience of availing treatment and coverage benefits in the light of what is envisaged in the guideline can help policymakers and programme planners for timely and adequate midcourse corrections. [9] The responsiveness of the health insurance scheme like PM-JAY can also be an important element of monitoring and evaluation of important health insurance system.

In this context, this research aims to understand the experiences of beneficiaries who have availed services under PM-JAY from empanelled public and private hospitals. These include a) experience of beneficiary identification and authentication, b) experience of hospitalization and scope of services received (e.g. free medicines), c) information exchange during beneficiary verification (e.g. progress in verification, and details of scheme and coverage to a verified beneficiary), before hospitalization (e.g. receipt of SMS informing them about details of packages and balance), during discharge ( e.g. receipt of discharge summary and copies of diagnostic reports, and SMS), and after hospitalization (e.g. follow up call from the designated call centre to ask about the hospitalization experience), and d) perception of overall quality of services, including additional payments made to the hospital, over and above the cashless payment provided to hospitals under PM-JAY.

By aiming to understand the experiences of PM-JAY beneficiaries, this study enriches the existing PM-JAY literature base and provides actionable insights to strengthen the scheme.

## Methodology

The study was conducted during March-August 2019. Data for this study were obtained through a household survey of beneficiaries. Gujarat and Madhya Pradesh (MP) were selected as study locations, in consultation with the NHA. The study sites included seven hospitals from each state, selected by the respective SHA after ensuring representation of a) public and private hospitals, and b) multi-speciality and super-speciality hospitals. As can be seen from Table 1, the sampled fourteen hospitals comprised nine multi-speciality and five super-speciality hospitals. Of the fourteen hospitals, eight were private hospitals and the rest were public hospitals, two of which were autonomous hospitals.

The study population included patients who were hospitalized in April 2019. The sample selection was to be done from the list of all claims from study hospitals as provided by the respective SHA of the two study states. Towards this end, the claims list was first converted into a patient list by retaining one claim per patient, as needed in case of multiple claims filed for one beneficiary. Next, to ensure the hospital-level representation of most and least popular services, it was decided to select most popular clusters and least popular clusters of specialty from each hospital. For each of the hospitals, the specialty clusters were arranged from those having highest number of patients to those having lowest number of patients. From this arrangement, starting from the top, those clusters that together contain 10% of the patients were identified as most popular cluster for that particular hospital. Similarly, starting from the bottom, those clusters that together contain 10% of the patients were identified as least popular cluster for that particular hospital. This list of patients, who availed the hospitalization for 'top-bottom' clusters, was then used to generate a sample of 100 beneficiaries in each state through probability proportional to size method using study hospital as the sampling unit. This meant that higher sample was allocated to the hospital with more number of claims and the smallest sample was derived from the hospital with the least number of claims. The distribution of patients that were surveyed across the types of hospitals can be found in Table 1. These sampled patients were from six districts in Gujarat and five districts in Madhya Pradesh. From among 100 patients of Gujarat, 39 were enrolled only in the then existing critical illness coverage i.e. Mukhyamantri Amrutam (MA) scheme.

**Table 1: Study hospitals and sample beneficiaries: a comparison by states**

| Hospital type | Ownership type | Gujarat | | Madhya Pradesh | |
|---|---|---|---|---|---|
| | | Number of hospitals | Sample of patients | Number of hospitals | Sample of patients |
| **Multi-specialty** | Private | 2 | 35 | 2 | 29 |
| | Public | 2 | 21 | 3 | 57 |
| **Super-specialty** | Private | 2 | 21 | 2 | 14 |

| | | | | | |
|---|---|---|---|---|---|
| | Public | 1 | 23 | - | - |
| **Grand Total** | | **7** | **100** | **7** | **100** |

The data collection was done during May-June 2019. A structured questionnaire was developed to include details of the patient's hospitalization experience, following the guidelines of PM-JAY. The questionnaire was reviewed and approved by the technical experts at WHO and the NHA. The revised instrument was then piloted for accuracy and validation, before commencing the data collection. This included a discussion with a panel of in-house experts and a field-testing of the instrument.

The patients or their main caregiver relatives were survey respondents and were interviewed at their place of residence. After providing a brief description of the study and its objectives, request to participate in the survey was made and verbal consent was sought from the respondents. The respondents were asked to indicate the reasons for and duration of their hospitalization. They were also asked in detail about their experiences and challenges around beneficiary identification and authentication, the experience of hospitalization and scope of services received (e.g. free medicines). To ascertain the overall quality of services, the respondents were asked to rate their experience of registration and hospitalization on a five-point Likert scale that ranged from 'highly dissatisfied' to 'highly satisfied'. To ascertain the effectiveness of the helpdesk services at the hospitals, respondents were asked in closed-ended questions if they received three types of help from the registration desk viz. a) information about PM-JAY, b) help with documents and computerized registration and c) guidance about treatment within the hospital. As a part of the patient survey, the respondents were asked to report how much time did the patient/ relatives spent during admission and pre-authorization request. To ascertain the extent of out-of-pocket payment (OOP), the respondents were asked to provide information on the additional payments made to the hospital over and above the cashless payment provided to hospitals under PMJAY. Lastly, the respondents were asked to

provide details of SMS and telephone calls that patient/relative may have received about registration, preauthorization, and discharge.

The structured questionnaire for the household survey was configured in the Open Data Kit (ODK) software, and data were collected using a hand-held electronic device. On-site monitoring and cross-verification of data collection were carried out to ascertain the reliability of data being collected. The data was cleaned using MS-Excel software and analyzed to generate descriptive statistics using STATA software version 12. The study received ethical clearance from the institutional ethics committee of the Indian Institute of Public Health Gandhinagar (IIPHG) vide letter TRC-IEC No: 14/2018-19.

## Results and Findings

The results and findings of the study are divided across five sub-sections, following the experiences of the beneficiary interactions with the system viz. a) registration, b) treatment package selection before hospitalization, c) preauthorization and admission during hospitalization, d) discharge and payments, and e) information exchange. Before presenting the results for the experiences of interactions, a summary of the socio-demographic profile of the respondents of the household survey is presented below as Table 2.

.

**Table 2: Profile of the sample beneficiaries ( In percentage)**

| Particulars | Details | Gujarat (n=100) | Madhya Pradesh (n=100) | Total (n=200) |
|---|---|---|---|---|
| Type of hospitals | Private | 56 | 43 | 49.5 |
| | Public | 44 | 57 | 50.5 |
| Gender | Female | 38 | 53 | 45.5 |
| | Male | 62 | 47 | 54.5 |
| Mean Age (year) | | 49.1 | 42.2 | |
| Religion | Hindu | 89 | 89 | 89 |
| | Muslim | 11 | 11 | 11 |
| Caste | Scheduled Tribe | 5 | 0 | 2.5 |
| | Scheduled Caste | 20 | 5 | 12.5 |
| | Other Backward Caste | 49 | 69 | 59 |

| | | | | |
|---|---|---|---|---|
| | Others (General) | 26 | 25 | 25.5 |
| | Refuse to answer | 0 | 1 | 0.5 |
| Occupation | Farm Labour | 18 | 4 | 11 |
| | Other labour work in a rural area | 8 | 7 | 7.5 |
| | Labour work in an urban area | 14 | 22 | 18 |
| | Self-employment(agricultural work) | 20 | 7 | 13.5 |
| | Self-employment (non- agricultural) | 19 | 38 | 28.5 |
| | Salaried job | 19 | 22 | 20.5 |
| | Others | 2 | 0 | 1 |
| The proportion of respondents who received benefits under state-sponsored health coverage before PMJAY | | 75 | 3 | 39 |

Of the 200 respondents, 39% had 'Hospitalization with surgery', 37% reported 'Hospitalization without surgery', and the remaining one-fourth respondents received daycare treatment like dialysis or sessions of cancer treatment. These proportions were different across states and ownership of hospitals. Among the patients who availed services in public hospitals of Gujarat, the proportion of day-care procedures was 61%. In Madhya Pradesh, a higher proportion of respondents (61%) from the public hospitals reported hospitalization without surgery. Around half of the respondents who availed treatment in private hospitals did so for surgical procedures in both the states. The details can be better understood through Figure 1 below.

**Figure 1 Proportion of beneficiaries by reasons for hospitalization: Comparison across the type of hospitals and state**

The average length of stay for hospitalization, excluding day care procedures, was 3.5 days for Gujarat and 6.1 days for Madhya Pradesh. This was higher for surgical hospitalization (6.6 days), followed by non-surgical hospitalization (5.6 days). Similarly, the average length of hospitalization was 4 days for private hospitals and 5.1 days for public hospitals. While there was not much difference across two types of hospitals in Madhya Pradesh (6.1 days for private and 6.2 days for public hospitals), private hospitals in Gujarat had a lower length of stay of 2.3 days, as compared to around 3.9 days in public hospitals of Gujarat. With this background, the

experiences of respondents along the various touchpoints of hospitalization are presented below.

### A. Beneficiary identification and registration

This involves awareness about the scheme and the experience of availing the e-card indicating beneficiary registration in the scheme. From among 100 patients of Gujarat, 39 were enrolled only in the then existing critical illness coverage i.e. Mukhyamantri Amrutam (MA) scheme, and were yet to be subsumed under PMJAY. These patients did not have registration experience. Therefore, the 'n' for Gujarat in this section is 61. A large proportion of PM-JAY beneficiaries in Gujarat (74%) indicated that they got to know about the scheme as they received a letter from the government indicating their entitlement. In MP, no such letter was reported by any respondent. Instead, around one-third of the beneficiaries (30%) got to know about the scheme when they visited the hospitals for hospitalization, and another one-fourth respondents got to know about the scheme from their friends and relatives.

As far as the checking of eligibility is concerned, most beneficiaries from Gujarat (82%) mentioned that the letter from the government indicated their eligibility as well. In Madhya Pradesh, around half of the respondents indicated that they checked their eligibility at the common service centres (Figure 2). This proportion was 63% among beneficiaries from the private hospitals, as around one-third of beneficiaries from public hospitals checked their eligibility at the hospital at the time of the admission.

**Figure 2: Proportion of beneficiaries by the source of eligibility checking: a comparison across states (In %)**

As for the effectiveness of the helpdesk services at the hospitals, half of the beneficiaries (52%) reported to have received information about PM-JAY, help with documents and computerized

registration and guidance about treatment within the hospital. The proportion of respondents who received all three kinds of help was highest in the public hospitals of Gujarat (82%), and lowest in private hospitals of Madhya Pradesh (35%). Only 7% respondents, mainly from a public hospital in Madhya Pradesh, indicated that they did not receive any help at the helpdesk. Less than one in ten beneficiaries reported that they faced problem in registration, in the form of long waiting time or payment.

As can be seen from Figure 3, 81% of respondents were either 'satisfied' or 'highly satisfied' with the registration process. This was higher in Gujarat (86%) as compared to Madhya Pradesh (77%).

**Figure 3: Proportion of beneficiaries by their level of satisfaction with registration: a comparison across states (In %)**

## B. Package selection and blocking

The self-reported average time for admission and pre-authorization request was half an hour in Gujarat and three hours in Madhya Pradesh. This duration was around one hour for the patients who availed treatment in public hospitals and more than five hours for those who were hospitalized in private hospitals of Madhya Pradesh. The average time for pre-authorization approval was four hours for the patients from Gujarat and nine and a half hours for patients from MP. While the patients who availed hospitalization from the public hospitals of MP reported a mean time of 1.7 hours, the beneficiaries of private hospitals reported a mean time of 19.5 hours for receiving approvals (Figure 4).

**Figure 4: Average time (in hours) in selected processes under PM-JAY hospitalization**

## C. Discharge and Out of pocket (OOP) payments

According to the patient's experience, the hospitals in Gujarat took around one hour to discharge, as compared to three hours in Madhya Pradesh. The possibility of additional payment at the time of discharge remains an important concern for the patients. As can be seen from Table 3, around one-fourth respondents (27%) reported that they paid either before, during or after hospitalization. A higher proportion of respondents who reported 'zero payment' were from Gujarat (89%) as compared to MP (57%), and from Public hospitals (80%) as compared to Private hospitals (66%). There was a higher incidence of OOP among patients of private hospitals in MP.

**Table 3: Incidence of OOP payments during hospitalization (In %)**

| Hospital type | Gujarat | Madhya Pradesh | Total |
|---|---|---|---|
| Private | 13<br>(n=56) | 63<br>(n=43) | 34<br>(n=99) |
| Public | 9<br>(n=44) | 28<br>(n=57) | 20<br>(n=101) |
| Total | 11<br>(n=100) | 43<br>(n=100) | 27<br>(n=200) |

On exploring reasons for the payment, it was found that most respondents from public and private hospitals indicated that these payments were for medicine and diagnostic investigations. Patients needed to make such payments directly to the pharmacy or laboratory as they were told that certain treatment inputs were either not available or not covered under the PM-JAY. In addition, around one-fifth respondents – mostly from private hospitals in Madhya Pradesh - indicated that they were asked to make a lump sum payment directly at the hospital counter. They were informed that the actual cost of the procedure was higher than the funds the hospital would receive in the form of claim reimbursement from the scheme, and thus, patients needed to pay the balance amount as part-payment.

In terms of the magnitude of the OOP payment, there was a significant difference across state and type of hospitals, as can be seen in Table 4. The patients who availed treatment in public hospitals in Gujarat had the lowest average OOP payment at INR 1550 (n=4) and the patients from private hospitals in MP paid highest average OOP (INR 40,996 (n=27)). The Gujarat patients, as well as public hospital patients of MP who reported lower OOP, spent on medicine and other supplies that were to be purchased from outside the hospital. Most of the high OOP respondents from private hospitals of MP either claimed for cancer-related procedures or cardiovascular procedures that involved the replacement of the valve or pacemaker. It is important to note that the average OOP was around three times higher for those patients who were asked by the hospitals to make 'part-payment' on top of the coverage (Mean expenses- INR. 47840, Median expenses- INR. 42,500), as compared to those who reported spending on medicine/tests/blood etc. (Mean expenses- INR. 17172 Median expenses – INR. 3250).

**Table 4: Average payment during hospitalization (In INR)**

| Hospital type | Gujarat | Madhya Pradesh | Total |
|---|---|---|---|
| Private | 5558<br>(n=7) | 40996<br>(n=27) | 31341<br>(n=34) |
| Public | 1550<br>(n=4) | 5123<br>(n=16) | 4409<br>(n=20) |
| Total | 4100<br>(n=11) | 27648<br>(n=43) | 22851<br>(n=54) |

As per the results of the survey, three—fourth respondents were either 'satisfied' or 'highly satisfied' with their hospitalization experience. This was higher in Gujarat (82%) as compared to MP (71%). The dissatisfied respondents were those who also reported the problem in the form of a) poor quality services in the form of the bad behaviour of healthcare providers, b) long waiting time during preauthorization and admission, and c) out-of-pocket expenses.

### D. Experience of receipt of SMS during and feedback call after hospitalization

As can be seen from Figure 5, in Gujarat, more than half of the patients reported receiving SMS during the verification step, while lower proportions of patients reported receiving SMS for preauthorization, admission and discharge. In Madhya Pradesh, a significantly lower proportion of patients reported receiving SMSs across processes.

**Figure 5: Proportions of beneficiaries who received SMS/call about specific processes (in %)**

The most common information exchange was a call that the beneficiaries received after the discharge. Nearly half of the beneficiaries in Madhya Pradesh and 60% in Gujarat reported having received a post-discharge feedback call. This proportion was 64% among public hospital beneficiaries of Gujarat and 40% among Madhya Pradesh beneficiaries who received services from public hospitals.

## Discussion

Good health, responsiveness and financial protection are three main elements of performance of the health system, in general, and health insurance scheme, in particular. This research looked at two distinct but related issues of responsiveness and financial protection through experiences of the beneficiary's interaction with the providers, processes, institutions, and information. This research provides evidence of such experiences across a range of touchpoints in a journey of the patient while they availed hospitalization services under PM-JAY. Also, it provides evidence on financial protection in the form of OOP expenses during hospitalization that influences an important objective of the scheme.

The source of information about the scheme and eligibility thereof through a letter before illness, as the case was with Gujarat, is a positive sign that may influence the treatment-seeking behaviour as the patients knew about the coverage before the onset of the illness. This is in contrast with getting to know about the scheme and eligibility only at the time of illness, as was the case with patients in Madhya Pradesh.

The interactions at the PM-JAY helpdesk remains crucial as a source of information about the hospitalization coverage at the time of the illness. The better performance of public sector hospitals in Gujarat and worst performance of private sector hospitals in Madhya Pradesh in this first contact point for patient interaction corroborated well with the pattern of patient satisfaction regarding beneficiary identification and registration. The time taken in various processes is an important element of responsiveness. The importance of the quality of non-clinical care was reiterated in the form of higher satisfaction among patients who experienced lower waiting time in package selection, admission, and discharge. The finding of the importance of prompt attention with low waiting time is similar to the one observed in the Nigerian social health insurance scheme, which highlighted the importance of a) autonomy in terms of the involvement of patients in decisions, b) communication of providers with beneficiaries, and c) prompt attention with low waiting time as priority areas for action to improve responsiveness. [9]

The high incidence and extent of OOP payment, especially in Madhya Pradesh, and corresponding poor patient satisfaction, is along the lines of similar GSHIS experiences in India. [18, 27, 28] Such OOP in PM-JAY could be connected with the design of the scheme in terms of process flow, poor package rates, and policy of government-reserved procedures. Many hospitals may insist on payment of pre-operative diagnostic procedures fearing that there is a possibility that the patient may not be hospitalized based on the negative results. The

policymakers needs to look into this design feature. Next, the patients in private hospitals may be asked to make payments in certain multimodal treatments that include hospitalization with surgical as well as medical treatment, which are exclusively reserved for public facilities. This is especially important for conditions like cancer where there is a need for customizing treatment by a team of doctors using multiple treatment modalities. Last, the patients may be asked to pay for the difference in expenses if the choice of treatment (for example, medicine or implant) is different from what is covered as a package reimbursement. [29] Irrespective of the reasons, the OOP affects financial and physical coverage and ultimately affects user satisfaction. If not addressed optimally, such payments can lead to a paradoxical situation of poor financial and physical coverage, despite an overall increase in the utilization of services. [30]

Lastly, beneficiary feedback - i.e. communication between the patients and healthcare as well as insurance providers as well and acting upon the inputs of the stakeholders - remains an important element of the responsiveness of the health insurance system. In PM-JAY, this integrated mechanism of information exchange with patients' i.e. timely provision of SMSs and calls for various hospital-based transactions found to have been performing sub-optimally with a scope of improvement in terms of its reach. The instances of poor patient satisfaction because of bad provider behaviour also points to the need for a mechanism to collect such information regularly and corrective actions thereof.

## Conclusion

In this research, the experiences of patients who availed hospitalization in the early phase of implementation of PM-JAY were examined. The research provided certain valuable insights about the ability of the scheme in being responsive and in providing financial protection. It highlighted the importance of prompt attention and timely access to information as important

attributes of non-clinical expectations of patients. To that end, functional helpdesks at the empanelled hospitals and a vibrant ICT- enabled two-way communication with beneficiaries have emerged as important elements to make the scheme more responsive. Such efforts will also contribute to patient-level efforts for insurance education. Access to better information may enable the beneficiaries to make informed choices and seek accountability, which ultimately can enhance the performance of the scheme.

Ensuring financial protection through reduced OOP expenses is an additional agenda of action for the planners of the scheme. The research highlighted that the existing high OOP in PM-JAY – as the forbidden practice of balance billing – is a result of intricacies in provider payment mechanisms and poor governance. While there is a need to streamline the existing treatment guidelines in accordance with prevailing standard practice, there would always remain a gap in optimal choices provided under the scheme and the best available options from the provider's perspective. It may be difficult for the planners to revise the existing package rates in the early stage of implementation due to the cost containment. However, it is important to ensure upward revision in the existing low rates of treatment packages with the expansion of coverage and utilization, to reduce the OOP and increase patient satisfaction in PM-JAY.

Lastly, the research pointed out that the policy of restricting certain procedures to public hospitals not only reduces financial coverage through OOP, but it also limits physical coverage and choice of providers – an important element of health system responsiveness. The package rates and the restriction of packages to government hospitals need to be rationalized by streamlining the existing treatment guidelines. Also, the information on the list of procedures reserved for government hospitals needs to be communicated effectively to the patients so that they are not caught unaware.

To sum up, this research contributes to the existing literature by providing insights from the beneficiaries based on their experience of hospitalization under PM-JAY. The results of this study emphasize the inter-connected issues of responsiveness and financial protection in the health insurance system, and highlight the changes that need to be made to strengthen the scheme. While there is thin literature on various aspects of PM-JAY being a new scheme, the research on responsiveness of GSHIS schemes, in general, are also few. In this context, this research provides valuable evidence and offers avenues of improvement in the implementation of PM-JAY.

**Acknowledgements**

We are thankful to the National Health Authority, India for the guidance and necessary approvals for the research. We also acknowledge the inputs of Dr. Grace Kabaniha (WHO, India) in improving the manuscript.

## References:


1. World Health Organization. The world health report: health systems financing: the path to universal coverage. Geneva: World Health Organization; 2010.
2. WHO. The World Health Report 2000: Health Systems : Improving Performance. Geneva: World Health Organization, 2000 9789241561983.
3. Murray CJL, Frenk J, World Health Organization. Global Programme on Evidence for Health P. A WHO framework for health system performance assessment / Christopher J. L. Murray, Julio Frenk. Geneva: World Health Organization; 1999.
4. Olmen Jv, Criel B, Bhojani U, Marchal B, Belle Sv, Chenge MF, et al. The Health System Dynamics Framework: The introduction of an analytical model for health system analysis and its application to two case-studies. 2012. 2012;2(1):21. Epub 2012-05-15. doi: 10.5195/hcs.2012.71.
5. Thi Thu Ha B, Mirzoev T, Morgan R. Patient complaints in healthcare services in Vietnam's health system. SAGE Open Medicine. 2015;3:2050312115610127. doi: 10.1177/2050312115610127. PubMed PMID: 26770804.
6. Paul S. Public Spending, Outcomes and Accountability: Citizen Report Card as a Catalyst for Public Action. Economic and Political Weekly. 2006;41(4):333-40.
7. Mohammed Ahmar U. Microinsurance in India: Insurance literacy and demand. Business and Economic Horizons (BEH). 2017;13(2):182-91 DOI: 10.15208/beh.2017.14. doi: DOI: 10.15208/beh.2017.14.
8. Cole S, Giné X, Tobacman J, Topalova P, Townsend R, Vickery J. Barriers to Household Risk Management: Evidence from India. American Economic Journal: Applied Economics. 2013;5(1):104-35. doi: 10.1257/app.5.1.104.
9. Mohammed S, Bermejo JL, Souares A, Sauerborn R, Dong H. Assessing responsiveness of health care services within a health insurance scheme in Nigeria: users' perspectives. BMC Health Services Research. 2013;13(1):502. doi: 10.1186/1472-6963-13-502.
10. Valentine NB, de Silva A, Kawabata K. Health system responsiveness: concepts, domains and operationalization. In: Darby C, Murray CJ, Evans DB, editors. Health systems performance assessment: debates, methods and empiricism. 96. Geneva: World Health Organization; 2003. p. 573–96.
11. Carrin G, James C. Reaching universal coverage via social health insurance: key design features in the transition period2004; 13(EIP/FER/DP.04.2). Available from: https://apps.who.int/iris/handle/10665/69018.
12. WHO. Everybody's business -- strengthening health systems to improve health outcomes : WHO's framework for action. Geneva: World Health Organization; 2007.
13. United Nations. Transforming our world: the 2030 Agenda for Sustainable Development. New York, USA,: United Nations Publications; 2015.
14. Giedion U, Andrés Alfonso E, Díaz Y. The impact of universal coverage schemes in the developing world: a review of the existing evidence. Wahington DC: The World Bank; 2013.
15. Escobar ML, Griffin CC, Shaw RP. The Impact of Health Insurance in Low- and Middle-Income Countries. Wasahington DC: Brookings Institution Press; 2011.
16. Garg S, Chowdhury S, Sundararaman T. Utilisation and financial protection for hospital care under publicly funded health insurance in three states in Southern India. BMC Health Services Research. 2019;19. doi: https://doi.org/10.1186/s12913-019-4849-8.
17. Prinja S, Chauhan AS, Karan A, Kaur G, Kumar R. Impact of Publicly Financed Health Insurance Schemes on Healthcare Utilization and Financial Risk Protection in India: A Systematic Review. PLOS ONE. 2017;12(2):e0170996. doi: 10.1371/journal.pone.0170996.
18. Devadasan N, Seshadri T, Trivedi M, Criel B. Promoting universal financial protection: evidence from the Rashtriya Swasthya Bima Yojana (RSBY) in Gujarat, India. Health Research Policy and Systems. 2013;11(1):29. doi: 10.1186/1478-4505-11-29.

19. Karan A, Yip W, Mahal A. Extending health insurance to the poor in India: An impact evaluation of Rashtriya Swasthya Bima Yojana on out of pocket spending for healthcare. Social Science & Medicine. 2017;181:83-92. doi: https://doi.org/10.1016/j.socscimed.2017.03.053.
20. Mirzoev T, Kane S. What is health systems responsiveness? Review of existing knowledge and proposed conceptual framework. BMJ Global Health. 2017;2(4):e000486 doi: 10.1136/bmjgh-2017-. doi: 10.1136/bmjgh-2017-000486.
21. La Forgia G, Nagpal S. Government-Sponsored Health Insurance in India: Are You Covered? New Delhi: World Bank; 2012.
22. Peters DH, Kanjilal B. Health insurance & responsiveness to communities & patients: the future of health systems in India. Indian J Med Res. 2011;133(1):9-10. PubMed PMID: 21321414.
23. NHA. Guidelines for Pradhan Mantri Rashtriya Swasthya Suraksha Mission (PMRSSM). 2018.
24. PIB. Cost of Treatment Under Ayushman Bharat PMJAY. In: GoI MoSHaFW, editor. Delhi: Press Information Bureau, GoI; 2019.
25. NHA. Lessons Learned in one year implementation of PM-JAY. New Delhi: National Health Authority, 2019.
26. NHA. PMRSSM GUIDELINES ON PROCESSES FOR HOSPITAL TRANSACTION. Delhi2018.
27. Sundararaman T, Muraleedharan V, Mukhopadhyay I. NSSO 71st round data on health and beyond: Questioning frameworks of analysis. 2016;51:85-8.
28. Nandi S, Schneider H, Dixit P. Hospital utilization and out of pocket expenditure in public and private sectors under the universal government health insurance scheme in Chhattisgarh State, India: Lessons for universal health coverage. PLOS ONE. 2017;12(11):e0187904. doi: 10.1371/journal.pone.0187904.
29. Trivedi M, Saxena A, Mavalankar D, Sharma M. Improving Hospital-Based Processes for Effective Implementation Of PMJAY: Evidence from Early Implementation. New Delhi: National Health Authority, 2019.
30. Gupta I, Chowdhury S, Trivedi M, Prinja S. Do health coverage schemes ensure financial protection from hospitalization expenses? Evidence from eight districts in India. Journal of Social and Economic Development. 2017;19(1):83-93. doi: 10.1007/s40847-017-0040-4.

Figure 1

Click here to access/download;Figure;Fig 1_300 dpi.tif

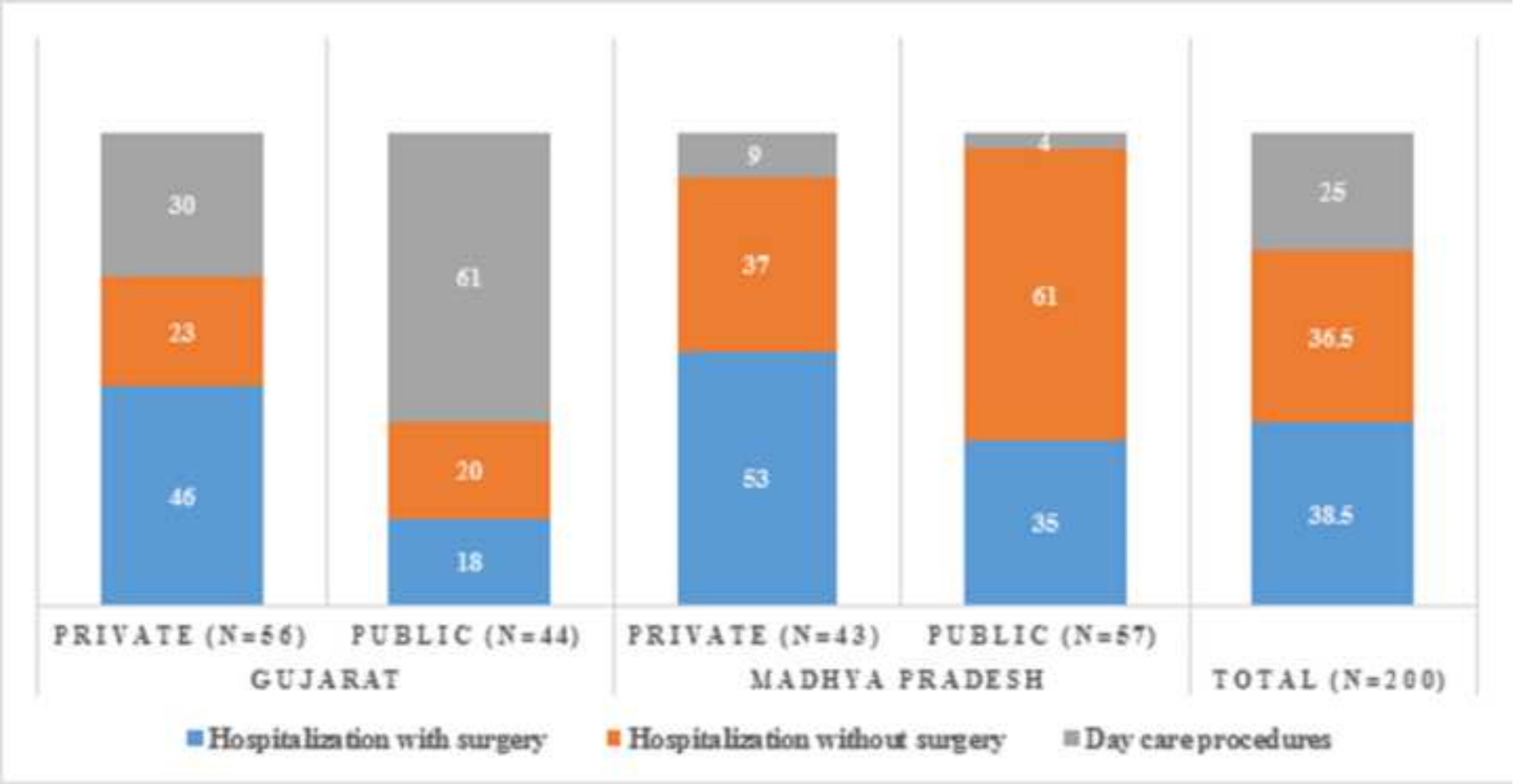

FIgure 2

Click here to access/download;Figure;Fig 2_300.tif

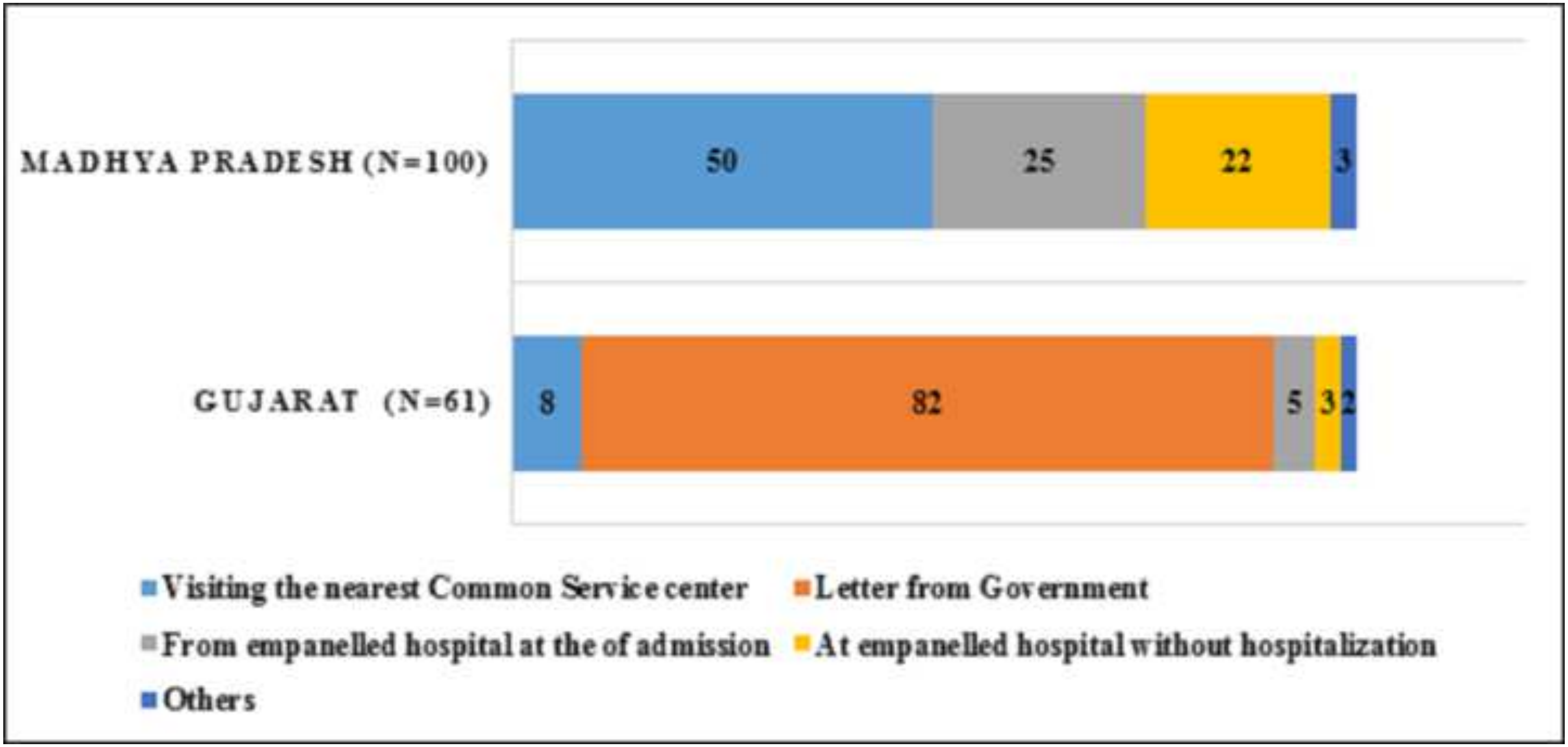

Click here to access/download;Figure;Fig 3_300.tif

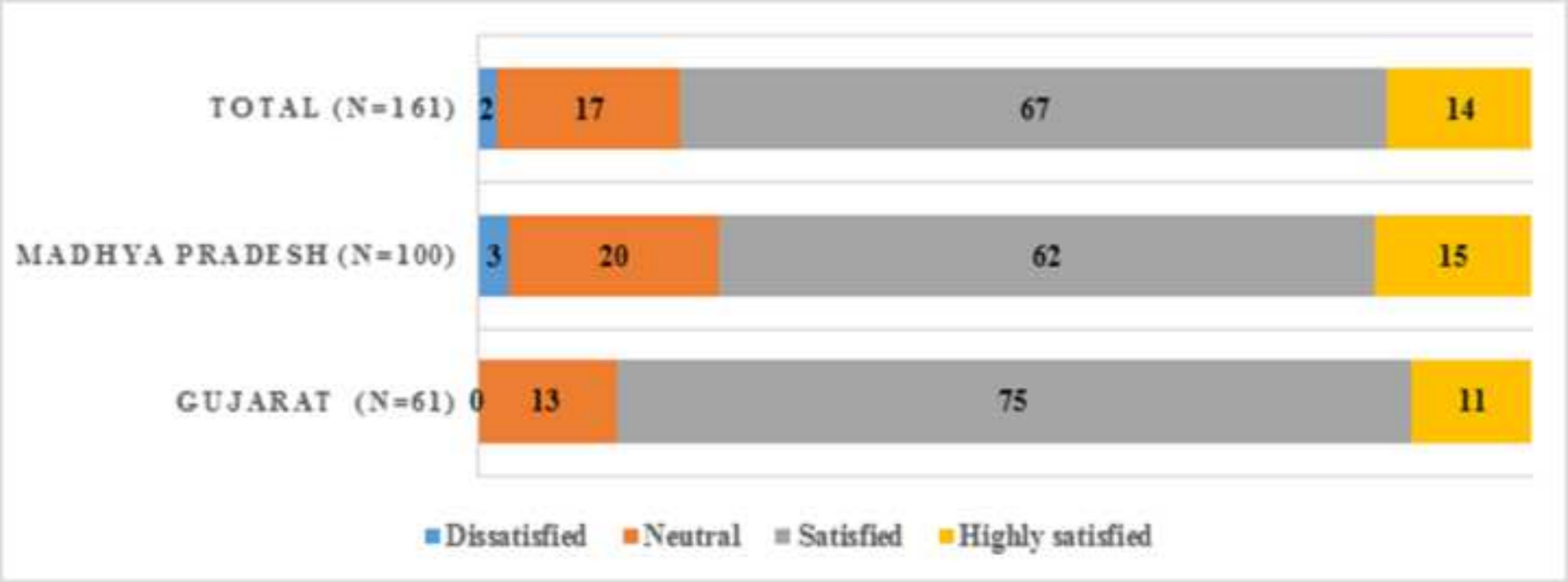

Figure 4

Click here to access/download;Figure;Fig 4_300.tif

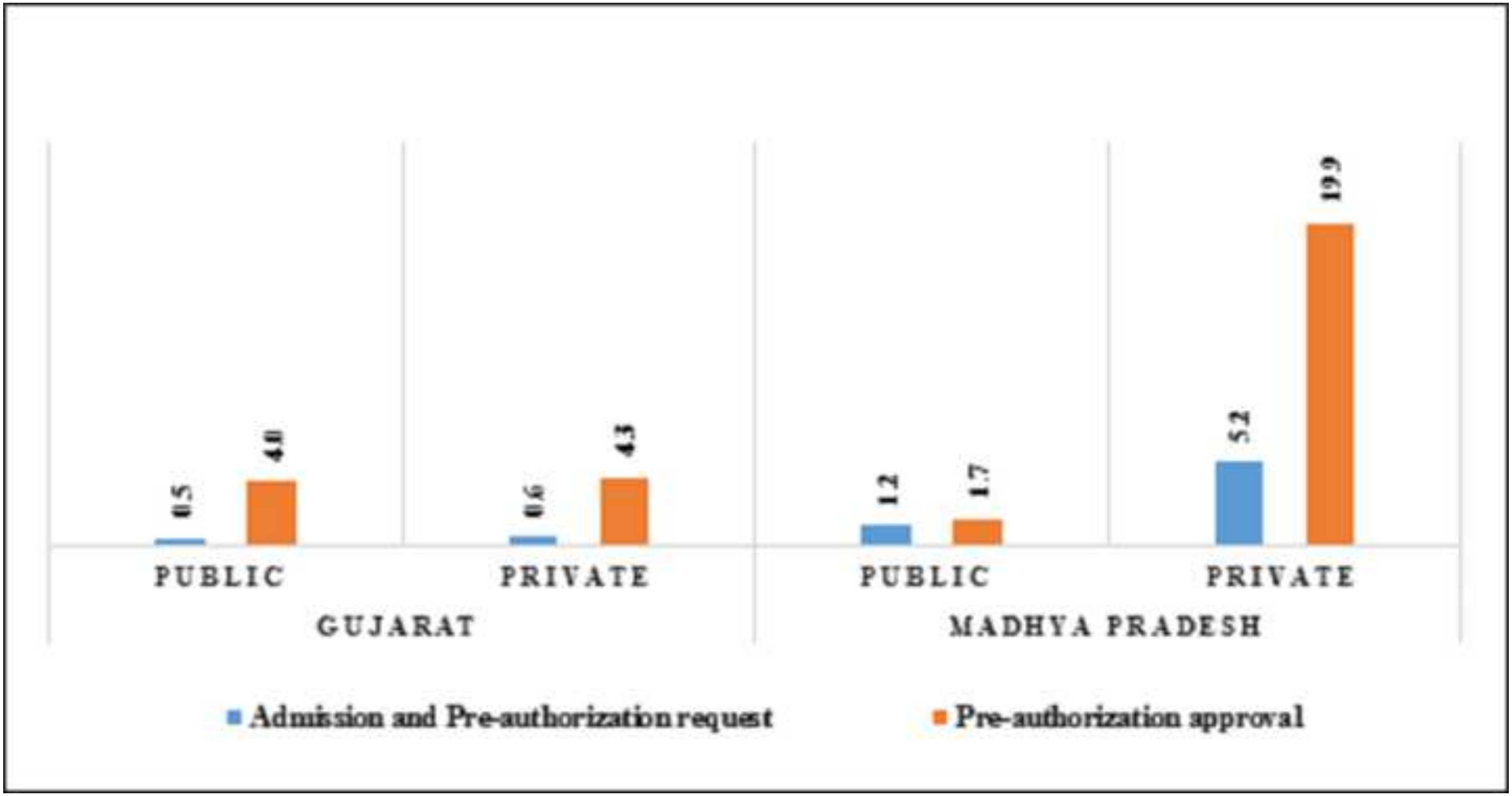

Figure 5

Click here to access/download;Figure;Fig 5_300.tif

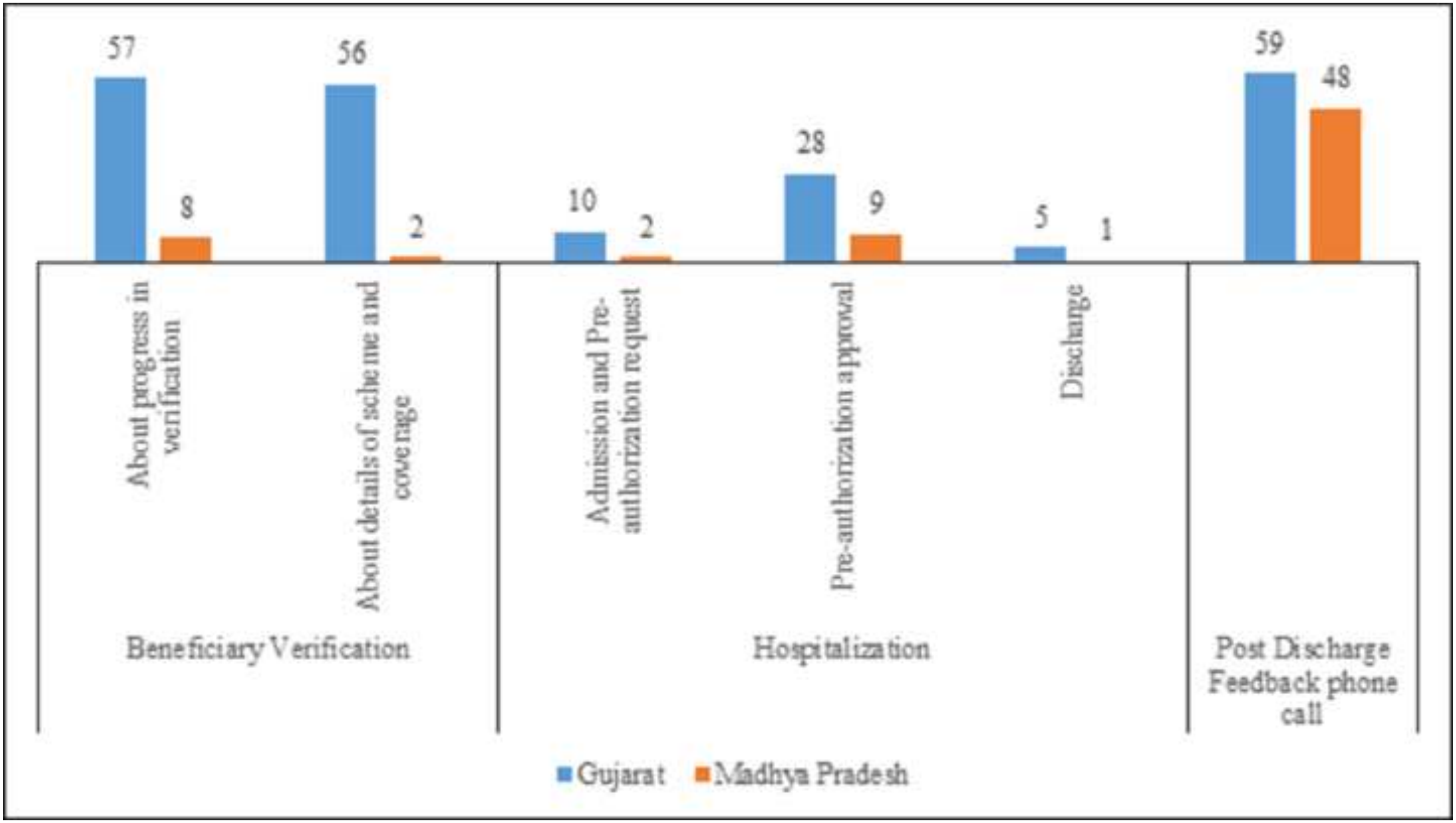

Supplement: Attachment — Submitted filename: PONE-D-21-06856- PMJAY scheme India - with comments.pdf [file pone.0266798.s003.pdf]
