# Supplementary material for: Experiences and challenges in accessing hospitalization in a government-funded health insurance scheme: Evidence from early implementation of Pradhan Mantri Jan Aarogya Yojana (PM-JAY) in India
Source: PLoS One. 2022 May 12;17(5):e0266798. doi: 10.1371/journal.pone.0266798 (PMC9098065; doi:10.1371/journal.pone.0266798)
Supplement: Attachment — Submitted filename: AnilEdit_PONE-D-21-06856_R1.pdf [file pone.0266798.s005.pdf]

PLOS ONE

Responsiveness and Financial Protection in a Government-Funded Health Insurance Scheme: Evidence from Early Implementation of Pradhan Mantri Jan Aarogya Yojana (PM-JAY) in India

--Manuscript Draft--

**Manuscript Number:**	PONE-D-21-06856R1
**Article Type:**	Research Article
**Full Title:**	Responsiveness and Financial Protection in a Government-Funded Health Insurance Scheme: Evidence from Early Implementation of Pradhan Mantri Jan Aarogya Yojana (PM-JAY) in India
**Short Title:**	Responsiveness and Financial Protection in India's PM-JAY Insurance Scheme
**Corresponding Author:**	Mayur Trivedi<br>Indian Institute of Public Health Gandhinagar<br>Gandhinagar, Gujarat INDIA
**Keywords:**	Health System Responsiveness, PM-JAY, India, Financial Protection, Universal Health Coverage, Health Insurance
**Abstract:**	Introduction : Improving health outcomes, assuring responsiveness and providing financial protection should be the key priorities of health systems and health insurance systems. This study explored the experiences of beneficiaries of Pradhan Mantri Jan Aarogya Yojana (PM-JAY), a government-sponsored health insurance scheme, in two states in India. The research asked recently hospitalized patients about their experiences of healthcare utilization, perceptions and overall satisfaction with the care they received, and the access they had to information from PM-JAY.<br>Methods : Data were obtained through a survey conducted with 200 PM-JAY beneficiaries (or their caregivers) in the Indian states of Gujarat and Madhya Pradesh. The study population was comprised of patients who received healthcare services at 14 study hospitals in April 2019. Data collection was done using a structured questionnaire and analyzed using Stata.<br>Results : Overall, patients generally reported feeling satisfied with their experiences of PM-JAY's coverage of their hospitalization, although satisfaction varied ~~depending on the state~~, the type of hospital they went to, and other factors. Patients' out-of-pocket expenses averaged 27,648 Indian rupees (US$ 398) during hospitalization. Out-of-pocket expenditures, waiting times, and other factors influenced the degree of financial protection and coverage patients experienced. Despite PM-JAY's protocols, less than half of patients indicated receiving either text messages or calls regarding various hospital-based transactions, indicating a particular need for the scheme to improve its communication with patients.<br>Conclusion : PM-JAY is a major new government-sponsored health insurance scheme in India. This study provides insights from its beneficiaries based on their experiences of hospitalization under PM-JAY. It emphasizes both the importance and the interconnectedness of responsiveness and financial protection for a health insurance system. This research provides valuable new evidence about the early implementation of PM-JAY and provides some concrete areas for improving its responsiveness and financial protection for beneficiaries.
**Order of Authors:**	Mayur Trivedi
	Anurag Saxena
	Zubin Shroff
	Manas Sharma
**Response to Reviewers:**	Reviewers' #1 comments:<br>General comments: The manuscript reviews major language editing and rewriting. There are far too many language errors across the manuscript, which makes it non readable.<br>All sections need major editing and shortening. The repetition should be eliminated. The discussion section needs to be improved with editing and in the quality of analysis.

Powered by Editorial Manager® and ProduXion Manager® from Aries Systems Corporation

	Response: We have revised the manuscript substantially with the help of a professional editor. This has reduced grammatical errors and improved the content.<br><br>Specific comment 1: Abstract need to be completely re-written. It does not capture the essence of manuscript. There are 200 study participants from whom data was collected. Some quantitative analysis findings should be part of the abstract, which currently focuses upon qualitative aspects only. The conclusion in abstract is generic. Please re-write. The acronyms can be avoided.<br>Response: We have revised the abstract to reflect these details.<br><br>Specific comment 2: Financial disclosure: Please confirm if this is full disclosure of grant or was there any other funding in addition to Health policy and system research at WHO HQ? Please also confirm if authors have acknowledged all the key people who contributed to this work?<br>Response: We have revised the financial disclosure statement. It now reads, "The research was made possible through the financial support provided by the World Health Organization. This included support from the Alliance for Health Policy and Systems Research, WHO Geneva as well as from the WHO Country Office, India under grant numbers 68345 (WHO, Geneva) and 67378 (WHO, India). Support was provided under the research programme titled- Health Systems Research for PM-JAY: Improving hospital-based processes for effective implementation. The grant was received by Prof. Dileep Mavalankar, Prof. Mayur Trivedi, and Prof. Anurag Saxena of the Indian Institute of Public Health Gandhinagar. The grant period was from 4 March to 3 September 2019. The URL of the funder is www.who.int. Dr Zubin Shroff of WHO provided guidance in strengthening the methodology and finalizing the instruments, and reviewed the manuscript critically."<br>We have revised the acknowledgment statement. It now reads, "We are thankful to the National Health Authority, India, for the guidance and necessary approvals for the research. We acknowledge the inputs of WHO India team members Dr. Vinod Verma in conducting the research, and Dr. Grace Kabaniha in improving the manuscript. We would also like to thank Dr. Chandrakant Lahariya and Dr. Hilde De Graeve for their support and guidance. Please refer to line number 472-476.<br>Specific comment 3: Ethical statement: was there any specific reason that only verbal consent was sought and not the written consent?<br>Response: The respondents as poor beneficiaries of the PMJAY scheme were expected to have a low level of education to be poor and would be illiterate or semi-literate individuals from urban and rural areas. Since the data collection for the survey was done using handheld digital devices, the thumb impression was difficult to record. Therefore, informal verbal consent was obtained. However, the respondents were provided all details of the research, including their rights to refuse to participate and not answer any questions. We have provided these details in the manuscript. Please refer to line number 188-192.<br>Specific comment 4: Introduction / main text: The language need major re-writing for academic standards. The wordings such as; line 3: 'people may remain unhappy with the health systems" could be avoided. This section is a bit superfluous and verbose. It need to edited and shortened. There are total 108 lines, it is difficult to follow and read. The information about PM-AY can be moved into a box.<br>Response: We have revised the introduction substantially. The introduction is also shortened by more than fifteen lines. The line "people may remain unhappy with the health systems" is also removed. The information on PMJAY Is now reduced and thus, not moved in a box.<br>Specific comment 5: Methods section, there is duplication of information and repetitions.<br>Response: We have revised the methodology section substantially to remove duplication of information and repetitions.<br>Specific comment 6: Results section. One of the limitations is that the small sample size and then then findings have been used in both tables and text.<br>Response: We have revised the results section to remove repetitions in the findings that have been used in both tables and text. We have acknowledged the small sample size as one of the limitations. Please refer to line number 438-443.<br><br>Specific comment 7: In discussion, the key actionable suggestions should be included. The limitations of this work need to be elaborated. More India specific literature review and citations should be used.

Powered by Editorial Manager® and ProduXion Manager® from Aries Systems Corporation

Response: We have revised the discussion section substantially. Certain key actionable suggestions were provided under the conclusion section. These are now shifted to the discussion section. Also, we have added India-specific literature while comparing our findings of awareness of schemes. Please refer to line number 379-381. We have also added literature on waiting time and satisfaction. Please refer to line number 397-398. Additionally, a comparison around OOP has already existed in the manuscript. Please refer to line number 404-406. The limitations of this work are added in the discussion section. Please refer to line number 438-443.

Specific comment 8: Conclusion: Should be succinct and single or maximum of two paragraphs.
Response: We have revised the conclusion section substantially. After shifting certain key actionable suggestions to the discussion section, the conclusion section is now of two paragraphs only.
Specific comment 9: Figure 1- 4 are repetition and can be removed.
Response: Agreeing to the suggestion, we have removed these figures, as the details are provided in the text. These are now provided as supplementary material.

Reviewer #2 comments:

Specific comment 1. The paper may give some context of health care utilization and expenditure about the states chosen
Response: We have now added this information, along with a Table. Please refer to line number 148-49 and Table 1 for these details.
Specific comment 2.Need to strengthen the discussion section with some comparison of the findings of PM-JAY with private health insurance schemes to judge the strengths and shortcomings of PM-JAY.
Response: We couldn't find relevant research on responsiveness and financial protection from the perspective of beneficiaries of private health insurance schemes. Thus, we could not add such a comparison of PMJAY with private health insurance schemes. However, we have compared our findings with other GSHIS. Please refer to line number 406-420 for these details.

Specific comment 3. Quantitative analysis may be augmented by estimating odds-ratios for the chosen indicators.
Response: The study did not analyze the associations or determinants of responsiveness (measured in terms of patient satisfaction) and financial protection (measured in terms of incidence and magnitude of OOP). Estimation of odds ratio was therefore not attempted as it was not related to the study objectives. Additionally, small sample size was a limitation of the study restricting the scope of statistical analysis. We, however, explored the subgroup difference in responsiveness and financial protection across socio-economic categories of the respondents. These details are presented in Table 1 as supplementary material.
Specific comment 4. The word "improving" may be dropped from the title.
Response: Agreeing to the suggestion, we have removed the word 'improving' from the title.

Specific comment 5. There are many grammatical errors and many sentences need editing to have a more terse presentation.
Response: We have revised the manuscript substantially with the help of a professional editor. This has reduced grammatical errors and improved the content.
Specific comment 6. In line 251, the figures in the Table and in the description do not match.
Response: These were because the findings were presented to highlight the proportion of the respondents who did not make any OOP payments. We have now revised the language and figures to reflect the information in terms of those who made the OOP payment, as presented in the table
Specific comment 7. Line 274, and other lines, the style of writing figures is not uniform.
Response: We have corrected the style of writing figures to make them uniform at all places.

Powered by Editorial Manager® and ProduXion Manager® from Aries Systems Corporation

	Specific comments in the attached document:<br>Comment1: check grammar.<br>Response: We have revised the abstract and edited it for grammatical errors.<br>Comment2: The abbreviation in title and text should be the same, otherwise need to repeat the full form here as well.<br>Response: We have revised the abbreviation in the title.<br>Comment3: Indicate dollar values also<br>Response: We have revised the abstract and added dollar values.<br>Comment4: delete. Repeated<br>Response: We have revised the abstract accordingly.<br>Comment5: Avoid abbreviation<br>Response: We have revised the abstract accordingly.<br>Comment6: The conclusion seems more like objective and does not follow from results.<br>Response: We have revised the conclusion part of the abstract accordingly.<br>Comment7: Very long introduction. Clearly provide the background in the context of the objective of the study.<br>Response: We have revised the introduction substantially. The introduction is also shortened by more than fifteen lines.<br>Comment8: Please place references as per the plos one style - different places before and after full stops.<br>Response: We have revised the references as per PLOS style. They are now after full stops at all places.<br>Comment9: check grammar.<br>Response: We have revised the entire document for grammatical errors.<br>Comment10: Major features to be more succinct and summarized in a box under headings<br>Response: We have revised the introduction of PM-JAY substantially and now it is shorter, and in the flow of the text. At this stage, it need not be in the box. However, if the reviewer feels appropriate, we would be happy to put the details in the box.<br>Comment11: Please provide full references with links and dates accessed as per PLOS style.<br>Response: We have revised this reference. We have used the ‘PLOS’ style in the EndNote referencing software for generating full references for this manuscript.<br>Comment12: what are autonomous hospitals<br>Response: These are publicly funded hospitals that have special status through legislation and thus, are governed independently. However, since this was not relevant for the objectives, we have removed this sentence.<br>Comment13: what if the claim os from the same episode of ailment.<br>Response: All patients with single claims were retained in the population list. Few patients required multiple sessions of treatment like dialysis and chemotherapy. In such cases, each session was claimed separately, and thus, there were multiple claims of those patients. We retained all these patients.<br>Comment14: Would this not add a bias for responsiveness, as the hospitals with least claims can also be the ones where people did not want to go.<br>Response: The hospitals for the study were selected by the state authorities. It was important to ensure the representation of patients across hospitals and specialties within the hospitals. The use of multi-stage sampling method with the use of Probability Proportional to Size ensured that the selection of respondents was not biased either in favour or against any type of specialty and hospital. This information is now explained in the manuscript. Please refer to line number 169-171.<br>Comment14: They would already have better knowledge of the health scheme, so their responsiveness may be better.<br>Response: The selection of the state with previous experience of implementing a GSHIS was an element of study at the time of selection of the study location. The responsiveness in terms of their registration in the scheme was not analyzed and therefore, ‘n’ for the beneficiary registration did not include these patients. The responsiveness of their hospitalization experience was measured only for the particular episode during the study duration. Thus, such possibility of their experience influencing the knowledge and responsiveness of the scheme may be ruled out.<br>Comment15: Can be added as supplementary material<br>Response: We have submitted a copy of the questionnaire as supplementary material.<br>Comment15: Would this not create seasonal bias<br>Response: The study population includes patients who availed hospitalization under

PMJAY during April 2019. This was given by the state authorities, and thus, was not under the control of the researchers. The data collection was done during May-June 2019. There could be seasonal effects on the type of illnesses, but the responsiveness and financial protection may not get affected by the time of the year.

Comment16: Can say informed consent was taken

Response: We have revised the statement and provided more details in the manuscript. Please refer to line number 190-194.

Comment17: These can be summarized under different headings in a box - in the same format as the results are presented. Some of it also repeated from above. Need to add them together.

Response: These are now well-defined and discussed in the methodology section between lines 196-211. We have removed the repetition. The results of this study were documented using the flow of processes in the standard official guideline as a template. The findings are therefore divided into subsections that correspond to the PM-JAY guidelines for hospital-based transactions. These sub-sections are beneficiary identification and registration, treatment package selection and blocking, discharge and payments, and information exchange.

Comment18: Is anything covered before and after hospitalization. Was information collected on experience before and after hospitalization? If referral etc. had the influence on registration process. What about coverage after hospitalization.

Response: The study collected information on pre-and post-hospitalization expenses in detail. The analysis of OOP payment includes these details in light of the provision of such coverage under the scheme. The information exchange is an in-built element regarding a) registration done before or at the time of the hospitalization, b) package selection during hospitalization, and c) calls for feedback after the hospitalization. These were studied in detail.

Comment19: why is education of beneficiary/ or highest education in HH not taken as indicator, as awareness and responsiveness depends quite a bit on that.

Response: We have now added this information in Table 3.

Comment20: mismatch - 61 vs 74. Also if they were already enrolled in a scheme, one would expect better experience about the process. Could it be they did not want to switch.

Response: This is not a mismatch. 74% of the total 61 respondents (i.e. 45 respondents) indicated that they knew about the scheme from the letter. The remaining 39 participants at the time of the research were yet to be transferred from the MA scheme to PMJAY. For all practical purposes, all beneficiaries, irrespective of the scheme received the same coverage for their hospitalization. We did not include these 39 participants for their registration experience as they were not registered in PMJAY till April 2019. The participants did not have choice in switching as the existing scheme was getting subsumed under PMJAY over time.

Comment21: what was the difference in public vs. private will be useful to know

Response: The satisfaction was higher among beneficiaries from public sector hospitals (83%) than their counterparts from the private sector (77%). This is now added in the result section. Please refer to line number 296 and 297.

Comment22: You mention preauthorization above also. These are longer than for admission and preauthorization. Do you mean only admission above? Otherwise hours should be more above.

Response: The first mention of pre-authorization is in the context of ‘admission and pre-authorization request’ – i.e. time that the AM took in preparing and submitting the request. The second mention is about ‘pre-authorization approval’ to be received from the scheme authorities. The first one is the efficiency of the insurance processes at the level of healthcare providers, and the latter is about the efficiency of the scheme managers at the level of the insurance company / Trust. These details are clarified and streamlined in the subsection of ‘treatment package selection and blocking’ in the result section.

Comment23: Why is the sum of total smaller than public plus private

Response: The total of ‘n’ is 100 (after adding 56 private and 44 public). The incidence of OOP is in proportion. So, the total need not be the summation of the two proportions. It is the mean of the two (i.e. 13 and 9).

Comment24: Does PMJAY not cover the medicine bills for medicines not available. Also for a procedure, would everything not be covered?

Response: Ideally, all expenses should be covered. According to the guideline, the patient need not pay and the hospital must not do balance billing. However, the finding of this study, therefore, brings evidence on OOP and connects it to the reasons for

Powered by Editorial Manager® and ProduXion Manager® from Aries Systems Corporation

such OOP in the discussion section. We, therefore, raise the need to address underlying supply-side reasons for OOP.
Comment25: Are they informed and consulted about it before the registration.
Response: Yes. In most cases, the patients were informed beforehand about the possibility of such part payments. This was ascertained during the survey through informal discussions with the respondents.

Comment26: Is insurance agency or the scheme informed about these payments charged to the patients?
Response: The survey respondents did not know of such communication. Therefore, this is not part of our analysis. However, since part payment is not allowed under the scheme, there is no mechanism/ mandate for the hospitals to make this information available to the insurer/scheme managers.
Comment27: This is really high. Can this be compared with OOPS data from HH survey.
Response: This is high in the case of patients who availed of hospitalization in a private hospital in Madhya Pradesh, a state which saw such GSHIS with 5 lac coverage for the first time. Therefore, this needs to be seen contextually for the GSHISs covering secondary and tertiary hospitalization. We refrained from comparing the magnitude of OOP of this study (which was measured for those who made some payment) with similar data from the HH survey as the study population is not comparable. Our study population comprises those who exclusively opted for secondary and tertiary hospitalization, and thus are not comparable with households incurring expenses on a range of healthcare, including primary care and outpatient treatment. We have compared the incidence of OOP among the insured patients with similar research on beneficiaries of past GSHIS like RSBY that offered relatively low coverage. Please refer to line number 406-420 for these details.
Comment28: This should and most of it is part of introduction. Not required here.
Response: We have revised this paragraph and removed irrelevant sentences from here.
Comment29: What about the links to the socio economic conditions of the households. Also does education matter.

Response: The study did not analyze the determinants of responsiveness (measured in terms of patient satisfaction) and financial protection (measured in terms of incidence and magnitude of OOP). However, on comparing these parameters across the socio-economic subgroups of the respondents, it was found that the satisfaction for registration (91%) and hospitalization (82%) was highest among the illiterate respondents. This sub-group has the lowest incidence of OOP (15%), but reported the highest amount of OOP payment (Rs. 41075). We have provided this information with a table as supplementary material.

Comment30: What about the links to the rights and information. Also not discussed clearly about the payments and links to Aarogya Mitra. Should AM not play a role in helping the patients to keep their payments low. Even if AM desk is not there in hospitals, should AM not visit the hospitalized patient under the scheme. That would improve the quality and financial protection.

Response: Aarogya Mitra is a staff of the hospital appointed to streamline the liaison between patients, doctors, and scheme authorities. As an employee of the hospital, the AM interacts with the patients on behalf of the hospitals – at the helpdesk or bedside. The AM's interaction with the patient is limited to information and documentation for preauthorization and discharge. As for the payment, the AM merely conveys the decision of 'balance payment', if any, taken by the hospital authorities. Lastly, being a household survey of the patients, this research did not dwell deeper in the supply side issues of how AM can play an improved role at the provider level.
Comment31: how do this and some other conclusions follow from your results. Please restrict discussion and conclusions to what your results show.
Response: This is connected to the reasons that patients indicated for their OOP, especially in the case of those who made a lump sum payment. These patients reported that they were informed at the helpdesk that the actual costs of their treatments or procedures were higher than the amount the hospital would receive from claim reimbursements from PM-JAY; thus, patients needed to pay the balance. This is indicated in the result section (Please refer to line number 32-335). We have revised

	the language and added corresponding arguments and evidence (Please refer to line number 414-416).
**Additional Information:**	
**Question**	**Response**
**Financial Disclosure**<br><br>Enter a financial disclosure statement that describes the sources of funding for the work included in this submission. Review the submission guidelines for detailed requirements. View published research articles from *PLOS ONE* for specific examples.<br><br>This statement is required for submission and **will appear in the published article** if the submission is accepted. Please make sure it is accurate.<br><br>**Unfunded studies**<br>Enter: *The author(s) received no specific funding for this work.*<br><br>**Funded studies**<br>Enter a statement with the following details:<br>• Initials of the authors who received each award<br>• Grant numbers awarded to each author<br>• The full name of each funder<br>• URL of each funder website<br>• Did the sponsors or funders play any role in the study design, data collection and analysis, decision to publish, or preparation of the manuscript?<br>• **NO** - Include this sentence at the end of your statement: *The funders had no role in study design, data collection and analysis, decision to publish, or preparation of the manuscript.*<br>• **YES** - Specify the role(s) played.<br><br>* typeset	The research was made possible through the financial support provided by the Alliance for Health Policy and Systems Research, World Health Organization vide PO Purchase Order 202214458 for research entited as Health Systems Research for PM-JAY Improving hospital-based processes for effective implementation. The grant period was 4<br>March to 3 September 2019. The URL of the funder is www.who.int. Dr Zubin Shroff of WHO provided guidance in strengthening the methodology and finalizing the instruments, and reviewed the manuscript critically.
**Competing Interests**<br><br>Use the instructions below to enter a competing interest statement for this submission. On behalf of all authors, disclose any competing interests that could be perceived to bias this work—acknowledging all financial support and any other relevant financial or non-	The authors have declared that no competing interests exist.

financial competing interests.<br><br>This statement is required for submission and **will appear in the published article** if the submission is accepted. Please make sure it is accurate and that any funding sources listed in your Funding Information later in the submission form are also declared in your Financial Disclosure statement.<br><br>View published research articles from *PLOS ONE* for specific examples.<br><br>**NO authors have competing interests**<br><br>Enter: *The authors have declared that no competing interests exist.*<br><br>**Authors with competing interests**<br><br>Enter competing interest details beginning with this statement:<br><br>*I have read the journal's policy and the authors of this manuscript have the following competing interests: [insert competing interests here]*<br><br>* typeset	
**Ethics Statement**<br><br>Enter an ethics statement for this submission. This statement is required if the study involved:<br><br>• Human participants<br>• Human specimens or tissue<br>• Vertebrate animals or cephalopods<br>• Vertebrate embryos or tissues<br>• Field research<br><br>Write "N/A" if the submission does not require an ethics statement.<br><br>General guidance is provided below. Consult the submission guidelines for	The research was approved by the Institutional Ethics Committee at Indian Institute of Public Health Gandhinagar, India via letter TRC-IEC No: 14/2018-19. The respondents as poor beneficiaries of PMJAY scheme were expected to be poor and illiterate or semi-literate individuals from urban and rural areas. Since the data collection for survey was done using handheld digital devices, the thumb impression was difficult to record. Therefore, an informal verbal consent was obtained. However, the respondents were provided all details of the research, including their rights to refuse to participate and not answer any questions. All participants were provided with a participant information sheet that described the ethical concerns and emphasized the rights of the respondents. After providing a brief description of the study and its objectives, surveyors requested the respondent's participation in the survey and verbal non-witnessed informed consent was taken. The data was analyzed anonymously.

Powered by Editorial Manager® and ProduXion Manager® from Aries Systems Corporation

detailed instructions. **Make sure that all information entered here is included in the Methods section of the manuscript.**<br><br>**Format for specific study types**<br><br>**Human Subject Research (involving human participants and/or tissue)**<br>• Give the name of the institutional review board or ethics committee that approved the study<br>• Include the approval number and/or a statement indicating approval of this research<br>• Indicate the form of consent obtained (written/oral) or the reason that consent was not obtained (e.g. the data were analyzed anonymously)<br><br>**Animal Research (involving vertebrate animals, embryos or tissues)**<br>• Provide the name of the Institutional Animal Care and Use Committee (IACUC) or other relevant ethics board that reviewed the study protocol, and indicate whether they approved this research or granted a formal waiver of ethical approval<br>• Include an approval number if one was obtained<br>• If the study involved *non-human primates*, add *additional details* about animal welfare and steps taken to ameliorate suffering<br>• If anesthesia, euthanasia, or any kind of animal sacrifice is part of the study, include briefly which substances and/or methods were applied<br><br>**Field Research**<br><br>Include the following details if this study involves the collection of plant, animal, or other materials from a natural setting:<br>• Field permit number<br>• Name of the institution or relevant body that granted permission	
**Data Availability**<br><br>Authors are required to make all data underlying the findings described fully available, without restriction, and from the time of publication. PLOS allows rare	No - some restrictions will apply

Powered by Editorial Manager® and ProduXion Manager® from Aries Systems Corporation

exceptions to address legal and ethical concerns. See the PLOS Data Policy and FAQ for detailed information.<br><br>A Data Availability Statement describing where the data can be found is required at submission. Your answers to this question constitute the Data Availability Statement and **will be published in the article**, if accepted.<br><br>**Important:** Stating 'data available on request from the author' is not sufficient. If your data are only available upon request, select 'No' for the first question and explain your exceptional situation in the text box.<br><br>Do the authors confirm that all data underlying the findings described in their manuscript are fully available without restriction?	
**Describe where the data may be found in full sentences. If you are copying our sample text, replace any instances of XXX with the appropriate details.**<br><br>• If the data are **held or will be held in a public repository**, include URLs, accession numbers or DOIs. If this information will only be available after acceptance, indicate this by ticking the box below. For example: *All XXX files are available from the XXX database (accession number(s) XXX, XXX.*).<br>• If the data are all contained **within the manuscript and/or Supporting Information files**, enter the following: *All relevant data are within the manuscript and its Supporting Information files.*<br>• If neither of these applies but you are able to provide **details of access elsewhere**, with or without limitations, please do so. For example:<br><br>*Data cannot be shared publicly because of [XXX]. Data are available from the XXX Institutional Data Access / Ethics Committee (contact via XXX) for researchers who meet the criteria for*	This data contained in this study were made available to the research team by the National Health Authority, India for exclusive use in this study. The data belong to the National Health Authority. For more information please contact Dr Ruchira Agrawal , Consultant M&R, National Health Authority at Mel.cons1@nha.gov.in ."

access to confidential data.<br><br>*The data underlying the results presented in the study are available from (include the name of the third party and contact information or URL).*<br>• This text is appropriate if the data are owned by a third party and authors do not have permission to share the data.<br><br>* typeset	
Additional data availability information:	Tick here if your circumstances are not covered by the questions above and you need the journal's help to make your data available.

Powered by Editorial Manager® and ProduXion Manager® from Aries Systems Corporation

Manuscript Click here to access/download;Manuscript;Manuscript.docx

Full Title: Responsiveness and Financial Protection in a Government-Funded Health Insurance Scheme: Evidence from Early Implementation of Pradhan Mantri Jan Aarogya Yojana (PM-JAY) in India

Short Title: Responsiveness and Financial Protection in India's PM-JAY Insurance Scheme

Authors: Mayur Trivedi[1 *], Anurag Saxena[1], Zubin Shroff [2], Manas Sharma[1]

Author Affiliations:

[1] Indian Institute of Public Health, Gandhinagar, Gujarat, India

[2] World Health Organization, Geneva, Switzerland

* Corresponding Author: Mayur Trivedi, e-mail: mtrivedi@iiphg.org

DECLARATIONS

Ethical Approval: The research was approved by the Institutional Ethics Committee at the Indian Institute of Public Health Gandhinagar, India via letter TRC-IEC No: 14/2018-19.

Availability of data and materials: This data contained in this study were made available to the research team by the National Health Authority, India for exclusive use in this study. The data belong to the National Health Authority. For more information please contact Dr Ruchira Agrawal , Consultant M&R, National Health Authority at Mel.cons1@nha.gov.in ."

Competing interests: The authors declare that they have no competing interests.

Abstract

Introduction: Improving health outcomes, assuring responsiveness and providing financial protection should be the key priorities of health systems and health insurance systems. This study explored the experiences of beneficiaries of Pradhan Mantri Jan Aarogya Yojana (PM-JAY), a government-sponsored health insurance scheme, in two states in India. The research asked recently hospitalized patients about their experiences of healthcare utilization, perceptions and overall satisfaction with the care they received, and the access they had to information from PM-JAY.

Methods: Data were obtained through a survey conducted with 200 PM-JAY beneficiaries (or their caregivers) in the Indian states of Gujarat and Madhya Pradesh. The study population was comprised of patients who received healthcare services at 14 study hospitals in April 2019. Data collection was done using a structured questionnaire and analyzed using Stata.

Results: Overall, patients generally reported feeling satisfied with their experiences of PM-JAY's coverage of their hospitalization, although satisfaction varied depending on the state, the type of hospital they went to, and other factors. Patients' out-of-pocket expenses averaged 27,648 Indian rupees (US$ 398) during hospitalization. Out-of-pocket expenditures, waiting times, and other factors influenced the degree of financial protection and coverage patients experienced. Despite PM-JAY's protocols, less than half of patients indicated receiving either text messages or calls regarding various hospital-based transactions, indicating a particular need for the scheme to improve its communication with patients.

Conclusion: PM-JAY is a major new government-sponsored health insurance scheme in India. This study provides insights from its beneficiaries based on their experiences of hospitalization under PM-JAY. It emphasizes both the importance and the interconnectedness of responsiveness and financial protection for a health insurance system. This research provides valuable new evidence about the early implementation of PM-JAY and provides some concrete areas for improving its responsiveness and financial protection for beneficiaries.

Keywords: Health System Responsiveness, PM-JAY, India, Financial Protection, Universal Health Coverage, Health Insurance

Introduction

An important goal of the health system of any country is to improve the health outcomes of its citizens. However, even a health system that successfully attains better health outcomes can be deemed unsuccessful if it fails to please users. Users can disapprove of a health system if treatment costs are too high or if they perceive a lack of responsiveness to their felt needs. Health systems must therefore also strive to provide financial protection and to respond to the expectations of the population as it works to preserve, promote, and improve their health. [1]

Health system responsiveness is defined as "the ability of the health system to meet the population's legitimate expectations regarding their interaction with the health system, apart from expectations for improvements in health or wealth." [2] Responsiveness includes both physical and affective support during treatment. It incorporates elements of respect for and orientation to the rights of clients, including confidentiality, prompt attention, choice of providers, and the quality of amenities. The flow and clarity of information between the health system and its patients also form an important element of responsiveness. [3] Information asymmetries between patients and providers are typical in healthcare—however, a good health system provides sufficient information to patients to enable them to make informed decisions on the course of treatment or other actions recommended by providers. Health systems should also have mechanisms to solicit feedback from patients in order to gather information for strengthening service provision. In addition, government programs can use feedback from users to inform policy reforms. [4-6]

Much of the published discourse around health system responsiveness to the experiences and expectations of patients is limited to perspectives from developed country settings. Only a few studies have explored it in the context of social health insurance schemes in developing countries. [7-9] Responsiveness is especially essential for government-sponsored health

insurance schemes (GSHIS) that seek to ensure citizens' coverage by healthcare service delivery through improved strategic purchasing of healthcare services. [2, 10] GSHIS have been advocated as a means for governments to fulfil their responsibilities to citizens, including their commitments to move towards the Universal Health Coverage (UHC) envisioned under target 3.8 of the United Nation's Sustainable Development Goals (SDGs). [11]

In the past two decades, many low- and middle-income countries have introduced GSHIS of different scales and scopes. While many of these schemes have been effective in improving access and utilization of secondary and tertiary healthcare services [12, 13], there is evidence that out-of-pocket (OOP) expenses lead to dissatisfaction among the beneficiaries [14-17]. Beyond financial protections, GSHIS responsiveness also needs to be improved through cultivating better interactions of beneficiaries with providers, processes, and institutional arrangements. [18]

India is one of the countries that has been rapidly expanding health insurance coverage. The country has experience with both state-level and pan-Indian GSHIS. [19] These efforts culminated in 2018 in the launch of a nationwide GSHIS called Pradhan Mantri Jan Aarogya Yojana (PM-JAY). For successful implementation, this nationwide insurance scheme needs to cultivate its responsiveness to enrollees by considering enablers of and barriers to healthcare utilization, patients' perceptions of satisfaction, and access to information. [20]

The Government of India launched PM-JAY in September 2018, to provide insurance coverage to approximately 500 million beneficiaries from the 100 million poor and vulnerable families that form the bottom 40% of the Indian population. [21] The scheme is being rolled out in active partnership with the states. In many states with a history of implementing GSHIS, PM-JAY subsumed existing health insurance schemes, such as the Mukhyamantri Amrutam scheme in Gujarat. In states where there was no pre-existing scheme, such as Madhya Pradesh,

PM-JAY was launched as a new scheme. The state governments are implementing the scheme through a trust or an insurance company, that empanels the hospitals, approves the pre-authorization process, and makes payment to the hospitals.

The scheme provides coverage of up to INR 500,000 (USD 6800) per family per year to cover a package of medicines and diagnostics, including 1393 different surgery, medical and outpatient treatments at public hospitals and empaneled private hospitals. [22] Some procedures, deemed prone to fraud and abuse, are reserved for public hospitals only. Beneficiaries must bear the expenses for these procedures if treatment is availed in a private hospital. [23]

PM-JAY has established certain touchpoints for beneficiaries' interactions with the health insurance system when seeking treatment. Guidelines are clearly defined for each non-clinical step of the hospitalization process: registration, selection of treatment package before hospitalization, preauthorization and admission during hospitalization, discharge and payments, and information exchange at various touchpoints. [24] To support beneficiaries' engagement with PM-JAY, its guidelines state that all empaneled hospitals must place a dedicated PM-JAY helpdesk in a prominent location. The helpdesk is to be managed by an Ayushman Mitra (AM), a representative who manages operations and liaises between patients, doctors, hospital administration, and the scheme managers. The primary purpose of the helpdesk is to provide 'one-stop' services during registration, hospitalization, and discharge. The PM-JAY guidelines state that the AM should be able to use a diagnosis sheet provided by the doctor to select the treatment package(s) in the PM-JAY IT system. Once the package is selected and blocked, the request is submitted to the insurance company/trust for pre-authorization. According to guidelines, this request must be either approved or rejected within six hours. When the patient is discharged from the hospital, the AM similarly completes an online discharge summary form. The patient receives a discharge summary from the hospital.

Being a cashless scheme, patients are not supposed to make any payments during any stage of hospitalization. The patient is also expected to receive text messages via short message service (SMS) and calls regarding these processes from the scheme authorities. [25]

PM-JAY was designed to be an effective mechanism for ensuring health insurance coverage to a large number of economically poor Indian citizens. This population has previously been considered to be passive recipients of services from public and private sector healthcare, and neither sector has been especially responsive to their preferences. Policymakers currently lack sufficient knowledge about beneficiaries' experiences of navigating hospitalization processes and accessing information. While health system responsiveness depends on a range of factors, beneficiaries' expectations and experiences provide useful insight, both in the early phase of implementation as well as for midcourse corrections. [7] Tracking the responsiveness of PM-JAY (and other insurance schemes) is also an important element of monitoring and evaluation.

In this context, this research aims to understand the experiences of beneficiaries who utilized services under PM-JAY at empaneled public and private hospitals. Specific areas of interest include their experiences of: beneficiary identification and authentication; hospitalization and services received, including medicines; information exchange during beneficiary verification, before hospitalization, during discharge, and after hospitalization; and, perception of overall quality of services and any additional payments made to the hospital separate from the cashless payments by PM-JAY.

By documenting the experiences of PM-JAY beneficiaries, this study seeks to enrich the existing literature on PM-JAY responsiveness and provide actionable insights to strengthen the scheme.

Methodology

This study was conducted between March and August 2019. In consultation with the National Health Authority (NHA), Gujarat and Madhya Pradesh were selected as the study locations. Gujarat is an economically developed state with a higher annual per capita Gross State Domestic Product (GSDP), and a higher level of health expenditure (Table 1). Study sites included seven hospitals in each state; these were selected by the respective State Health Agency (SHA) after ensuring representation of a) public and private hospitals, and b) multi-speciality and super-speciality hospitals. As shown in Table 2, the fourteen hospitals included in the study comprised nine multi-speciality and five super-speciality hospitals. Eight of the hospitals were private and the rest were public hospitals.

Table 1: Comparison of study states across selected expenditure parameters

State		Gujarat	Madhya Pradesh
Gross State Domestic Product (GSDP) per capita (INR)*		224896	99025
State health expenditure (2019-20 Budget Estimate)*	Per capita (INR)	1610	1284
	Percent of GSDP	0.7	1.3
	Percent of total revenue expenditure	7.1	5.9
Per capita health expenditure (INR) (2015-16)**	Total	3703	2820
	Government	1429	811
	OOP	1781	1944
Average total expenditure on account of hospitalization per hospitalization case (INR) (2014)***	Rural	32503	19385
	Urban	26401	17117
Sources: * State Finances: A Study of Budgets of 2020-21, Reserve Bank of India [26]; ** National Health Accounts India (2015-16) [27] *** National Health Profile 2019 [28]			

The study population was drawn from patients who were hospitalized in April 2019. Sample selection was done using the list of all claims from study hospitals, as provided by the SHAs in the two states. Each claims list was converted into a patient list by retaining one claim per

beneficiary. Next, to ensure representation of most and least popular services at each hospital, the most and least popular specialties were selected. For each hospital, specialty clusters were arranged from those having the highest number of patients to those having the lowest. Those clusters that together contained the top 10% of patients were identified as the 'most popular cluster' for that hospital. Similarly, those clusters that together contained the bottom 10% of patients were identified as the 'least popular cluster' for that hospital. The resulting list of patients in the 'top - bottom' clusters was then used to generate a sample of 100 beneficiaries in each state through the probability proportional to size (PPS) method using the hospital as the sampling unit. Thus a higher proportion of the sample was drawn from the hospital with the most claims and the smallest sample was derived from the hospital with the fewest claims. The use of a multi-stage sampling method with the use of PPS ensured that the selection of respondents was not biased either in favour or against any type of specialty and hospital. [29] The final distribution of patients included in the study across the different types of hospitals is shown in Table 2. The sampled patients were from six districts in Gujarat and five districts in Madhya Pradesh. Among the 100 patients in Gujarat, 39 were only enrolled in the Mukhyamantri Amrutam critical illness coverage scheme that existed at the time, not the full PM-JAY scheme (which has since subsumed Mukhyamantri Amrutam).

Table 2: Study hospitals and surveyed beneficiaries, by state

Hospital type	Ownership type	Gujarat		Madhya Pradesh	
		Number of hospitals	Sample of beneficiaries	Number of hospitals	Sample of beneficiaries
Multi-specialty	Private	2	35	2	29
	Public	2	21	3	57
Super-specialty	Private	2	21	2	14
	Public	1	23	-	-
**Total**		**7**	**100**	**7**	**100**

Data for this study were obtained through a household survey of the sampled beneficiaries. A structured questionnaire was developed to solicit details of the patient's hospitalization

experience keeping in consideration the guidelines from PM-JAY. The draft questionnaire was reviewed and approved by technical experts at WHO and the NHA. Before data collection commenced, the revised instrument was piloted for accuracy and validation through a discussion with a panel of in-house experts and a field test. The survey questionnaire is available as supplementary material to this paper. The data collection was done by a team of six investigators in May and June 2019. Before going to the field, these investigators underwent a one-day training specifically designed for the data collection under this research. The questions were asked in the local language i.e. Gujarati for Gujarat and Hindi for Madhya Pradesh. Each survey administration lasted for 30-40 minutes.

The survey was administered to - either the patient or his/her primary caregiver - at their residences. They were contacted telephonically in advance to ascertain their availability for the survey. All participants were provided with a participant information sheet that described the ethical concerns and emphasized the rights of the respondents. After providing a brief description of the study and its objectives, surveyors requested the respondent’s participation in the survey and verbal non-witnessed informed consent was taken. Nine of the initially contacted respondents refused to participate in the survey. The consented respondents were asked to indicate the reasons for and duration of their hospitalization. They were asked in detail about their experiences and any challenges experienced during beneficiary identification and authentication, the experience of hospitalization and the scope of services received (including medicines). The responsiveness was measured in the form of a) effectiveness of helpdesk, b) time taken at different stages of hospitalization and discharge events, and c) information exchange between patient and scheme authorities. To ascertain the effectiveness of the helpdesk services, respondents were asked closed-ended questions about whether they had received three types of help from the registration desk: 1) information about PM-JAY; 2) help with documents and computerized registration; and, 3) guidance about treatment within the

hospital. Respondents were asked to provide details of any message and telephone calls received from the scheme authorities about registration, preauthorization, and discharge. Respondents were also asked to report how long the admission, pre-authorization request and discharge processes took. The financial protection was measured in terms of OOP payment by the patients. To ascertain the extent of OOP payments, respondents were asked to provide information on any additional payments made to the hospital separate from the cashless payment provided to hospitals by PMJAY. The quality of services was measured in the form of patient satisfaction. To ascertain the overall quality of services, respondents were asked to rate their experiences of registration and hospitalization on a five-point Likert scale that ranged from ‘highly dissatisfied’ to ‘highly satisfied’.

The questionnaire for the household survey was configured using Open Data Kit (ODK) software, a free and open-source tool developed by the University of Washington. The data were collected using hand-held electronic devices. On-site monitoring and cross-verification of data collection were carried out to ascertain the reliability of the data being collected. This was done by the research supervisors through observation and spot-checking. The data were cleaned using MS-Excel software and descriptive statistics were generated using STATA (Version 12), developed by StataCorp LLC. The study received ethical clearance from the institutional ethics committee of the Indian Institute of Public Health Gandhinagar (IIPHG) (TRC-IEC No: 14/2018-19).

Results

The results of the study are presented in six sections. The first section provides basic sociodemographic information on the 200 survey respondents, as well as an overview of the health care services they received during their hospital visits and their overall satisfaction. The following sections are organized based on the beneficiaries’ interactions with aspects of the

PM-JAY system: identification and registration; treatment package selection before hospitalization; discharge and payments; and, information exchange.

Respondents' characteristics and services received

Table 3 presents a summary of the socio-demographic profile of the survey respondents. With a high proportion of respondents having a low level of education, belonging to socially marginalized sections of scheduled caste and other backward castes, and fewer having a salaried job, it indicates that the PM-JAY is reaching the socio-economically poorer section of the society.

Table 3: Profile of the surveyed beneficiaries

Particulars	Details	Gujarat (n=100)	Madhya Pradesh (n=100)	Total (%)
Type of hospital	Private	56	43	49.5
	Public	44	57	50.5
Gender	Female	38	53	45.5
	Male	62	47	54.5
Mean age (years)		49.1 (Range 6-79)	42.2 (Range 2-75)	
Religion	Hindu	89	89	89
	Muslim	11	11	11
Caste	Scheduled Tribe	5	0	2.5
	Scheduled Caste	20	5	12.5
	Other Backward Caste	49	69	59
	Other (General)	26	25	25.5
	Refuse to answer	0	1	0.5
Highest level of education attained in the household	Illiterate or no formal education	39	15	27
	Primary education (1-8 standard)	35	50	42.5
	Secondary education (9-12 standard and diploma etc.)	23	26	24.5
	Class 12 and above	3	9	6
Occupation	Farm Labor	18	4	11
	Other labor work in a rural area	8	7	7.5
	Labor work in an urban area	14	22	18
	Self-employment (agricultural)	20	7	13.5
	Self-employment (non-agricultural)	19	38	28.5
	Salaried job	19	22	20.5
	Other	2	0	1
Respondents who received benefits under state-sponsored health coverage before PMJAY		75	3	39

In terms of the services received by the beneficiaries, 39% had hospitalization with surgery, 37% reported hospitalization without surgery, and the remaining respondents received daycare treatment as an outpatient without having to remain overnight at the hospital. These daycare treatments included dialysis or cancer treatment sessions. These proportions varied across states and type of hospital. Among the patients who utilized services in public hospitals in Gujarat, 61% received day-care procedures. In Madhya Pradesh, 61% of respondents from public hospitals reported receiving hospitalization without surgery. In both states, around half of the respondents who received treatment in private hospitals had surgical procedures.

The average length of stay for hospitalizations, excluding day care procedures, was 3.5 days for Gujarat and 6.1 days for Madhya Pradesh. Stays were longer for surgical hospitalization (6.6 days) than for non-surgical hospitalization (5.6 days). The average length of hospitalization was 5.1 days in public hospitals and 4 days in private hospitals. There was little difference between the two types of hospitals in Madhya Pradesh: 6.1 days for private and 6.2 days for public hospitals. Private hospitals in Gujarat, however, had noticeably shorter stays (2.3 days) than public hospitals (3.9 days).

Beneficiary identification and registration

The first touchpoint of the patient experience that was examined was beneficiary identification and registration. This involves beneficiaries' awareness of the insurance scheme and their experiences using the e-card indicating registration in the scheme. The number of respondents from Gujarat in this section is 61, as at the time 39 were enrolled in the Mukhyamantri Amrutam scheme, which had not yet been subsumed under PMJAY; these patients did not have a comparable registration experience.

A large proportion (74%) of PM-JAY beneficiaries in Gujarat indicated that they were made aware of the scheme through a letter from the government indicating their entitlement. Another

11% indicated that they got to know about the scheme through village level health workers and 7% got to know about the scheme only at the time of the hospitalization. The remaining respondents knew about the scheme from friends and relatives or through newspapers. None of the respondents from Madhya Pradesh reported receiving any such letter. Instead, nearly one-third of the beneficiaries (30%) learned about the scheme when they arrived at the hospital, while one-quarter of the respondents got to know about the scheme from their friends and relatives. The remaining sources included newspaper and internet, etc.

Most (82%) beneficiaries from Gujarat mentioned that the letter from the government indicated their eligibility for entitlements in the scheme as well. In Madhya Pradesh, around half of the respondents indicated that they checked their eligibility at common service centers, government-authorized one-stop-facility for availing digital services on various public schemes and entitlements. (Figure 1). This proportion was 63% among those using private hospitals in Madhya Pradesh; around one-third of beneficiaries using public hospitals only checked their eligibility at admission at the hospital.

Figure 1: Proportion of beneficiaries by the source of eligibility checking: a comparison across states (In %)

As mentioned, survey respondents were asked specifically about whether they received help with three aspects at the PM-JAY helpdesks. Half of the beneficiaries (52%) reported receiving information about PM-JAY, help with documents and computerized registration, and guidance about treatment within the hospital. The proportion of respondents who received all three kinds of help was highest in the public hospitals of Gujarat (82%), and lowest in the private hospitals of Madhya Pradesh (35%). Only 7% of respondents indicated that they did not receive any help at the helpdesk—these were respondents who had been at a public hospital in Madhya Pradesh.

Fewer than one in ten beneficiaries reported facing problems with registration in the form of long waiting time or payment before registration.

As shown in Figure 2, 81% of respondents felt either ‘satisfied’ or ‘highly satisfied’ with the registration process. This was higher in Gujarat (86%) than in Madhya Pradesh (77%), and among beneficiaries from public sector hospitals (83%) than their counterparts from the private sector (77%).

Figure 2: Proportion of beneficiaries by their level of satisfaction with registration: a comparison across states (In %)

Treatment package selection and blocking

PMJAY guidelines state that once the patient’ eligibility is ascertained, the AM should be able to block the benefit package(s) efficiently using the PMJAY IT system. The self-reported average time for ‘admission and pre-authorization request’ – i.e. time that the AM took in preparing and submitting the request - was 30 minutes in Gujarat and three hours in Madhya Pradesh. In Madhya Pradesh, the duration was approximately one hour for the patients at public hospitals and more than five hours for those who were hospitalized in private hospitals. The average time for the ‘pre-authorization approval’ to be received from the scheme authorities was four hours for the patients from Gujarat and nine and a half hours for patients from MP. Patients in the public hospitals of MP reported a mean time of 1.7 hours, while the beneficiaries at private hospitals reported a mean time of 19.5 hours for receiving approvals.

Discharge and payments

According to the patients, hospitals in Gujarat took around one hour to complete the discharge process, compared to 2.3 hours in Madhya Pradesh. While all hospitals took a similar time for

discharge in Gujarat, this was higher for private hospitals (3 hours) as compared to public hospitals (1.8 hours) in Madhya Pradesh. The possibility of having to make additional payments outside of insurance coverage at the time of discharge remained an important concern for the patients. As shown in Table 4, 27% of respondents reported that they paid OOP fees either before, during, or after hospitalization. A lower proportion of respondents who reported making OOP payments were from Gujarat (11%) as compared to 43% in Madhya Pradesh. A lower proportion (20%) of patients from public hospitals made OOP payment as compared to those at private hospitals (34%). The highest incidence of OOP was among patients of private hospitals in Madhya Pradesh.

Table 4: Incidence of OOP payments during hospitalization (%)

**Hospital type**	**Gujarat**	**Madhya Pradesh**	**Total**
Private	13<br>(n=56)	63<br>(n=43)	34<br>(n=99)
Public	9<br>(n=44)	28<br>(n=57)	20<br>(n=101)
Total	11<br>(n=100)	43<br>(n=100)	27<br>(n=200)

Most respondents who reported making OOP payments indicated they were for either medicines or diagnostic tests. Patients were required to make payments directly to the pharmacies or laboratories outside the hospitals, as they were told that certain inputs were either not available or not covered under the PM-JAY. In addition, around one-fifth of respondents who made OOP payments, mostly from private hospitals in Madhya Pradesh, indicated that they were asked to make a lump-sum payment directly at the hospital billing counter. These patients reported that they were informed at the helpdesk that the actual costs of their treatments or procedures were higher than the amount the hospital would receive from claim reimbursements from PM-JAY; thus, patients needed to pay the balance.

In terms of the amounts of the OOP payments, there were notable differences across states and types of hospitals, as shown in Table 5. The four patients who had to make OOP payments while receiving treatment in public hospitals in Gujarat had the lowest average amount: INR 1,550. Patients at private hospitals in Madhya Pradesh paid the highest average amount of OOP: INR 40,996 (n=27). Patients in Gujarat, as well as public hospital patients in Madhya Pradesh who reported lower OOP payments, had to purchase medicine and other supplies from outside the hospital. Most of the respondents from private hospitals of MP who made high OOP payments had claimed for either cancer-related procedures or cardiovascular procedures that involved the replacement of a valve or pacemaker. The average OOP payment was around three times higher for those patients who were asked by the hospitals to make a part-payment on top of their insurance coverage (mean: INR 47,840; median: INR. 42,500), when compared with those who reported paying OOP for medicines, tests, blood, or other individual costs (mean: INR 17,172; median expenses: INR 3,250).

Table 5 Average payment during hospitalization (INR)

Hospital type	Gujarat	Madhya Pradesh	Total
Private	5,558 (n=7)	40,996 (n=27)	31,341 (n=34)
Public	1,550 (n=4)	5,123 (n=16)	4,409 (n=20)
Total	4,100 (n=11)	27,648 (n=43)	22,851 (n=54)

Overall, a significant majority of survey respondents were either ‘satisfied’ or ‘highly-satisfied’ with their hospitalization experience. This was higher in Gujarat (82%), compared with Madhya Pradesh (71%), and among public hospitals (82%), compared with private hospitals (66%) Dissatisfied respondents reported problems, including bad behaviour on the part of healthcare providers, long waiting times during preauthorization and admission, and OOP expenses.

Information exchange before, during, and after hospitalization

The PM-JAY guidelines indicate that patients should receive information about various processes concerning their hospitalization through calls and SMSs, which are made by the scheme authorities. Figure 3 shows that more than half of the patients in Gujarat reported receiving messages via SMS during the verification step; lower proportions of patients reported receiving SMSs for preauthorization, admission and discharge. In Madhya Pradesh, a significantly lower proportion of patients reported receiving SMSs across processes.

Figure 3: Proportions of beneficiaries who received SMS/call about specific processes (in %)

The most commonly reported information exchange was a call after discharge soliciting their feedback. Nearly half of the beneficiaries in Madhya Pradesh and 60% in Gujarat reported having received a post-discharge feedback call. This proportion was 64% among public hospital beneficiaries of Gujarat and 40% among Madhya Pradesh beneficiaries who received services from public hospitals.

Discussion

Health outcomes, responsiveness, and financial protection are three main elements of the performance of any health system, including a health insurance scheme such as PM-JAY. This study looked at issues related to responsiveness and financial protection under PM-JAY by soliciting data on beneficiaries' experiences with providers, processes, institutions, and information. The research provides evidence on patient experiences across a range of touchpoints during hospitalizations covered by PM-JAY. It also provides evidence on the

degree of financial protection afforded by the scheme by quantifying OOP expenses during hospitalizations.

Overall, the study found that PM-JAY was relatively successful in both responsiveness and financial protection, although significant room for improvement on both fronts remains. The study also showed that patient experiences vary depending on what state they are in and what type of hospital they use.

To begin with, patients need basic information about their coverage under PM-JAY. In Gujarat, many patients had received a letter about the scheme and their eligibility before they experienced illness—this is a positive sign that the scheme is reaching out to beneficiaries. This is an improvement over poor information among the beneficiaries of similar GSHIS in Gujarat. [16, 30] Knowing about the nature of their coverage before the onset of an illness can influence patients' treatment-seeking behaviour. This contrasts with the less-ideal situation for most patients in Madhya Pradesh, who only learned about the scheme and their eligibility only at the time of illness and arrival at a hospital.

Interactions with the PM-JAY helpdesks are crucial sources of information about hospitalization coverage at the time of illness. Access to a helpdesk was correlated with overall patient satisfaction: public sector hospitals in Gujarat performed better and private sector hospitals in Madhya Pradesh performed poorly at this first contact point for patient interaction. These performance levels mirrored patient satisfaction regarding beneficiary identification and registration. Ensuring access to functional helpdesks at PM-JAY's empaneled hospitals and using ICT to enable two-way communication with beneficiaries have emerged as important tools that make the scheme more responsive to its clients and which need to be further expanded. These efforts will also contribute to patient-level insurance education.

The duration of various non-clinical processes was also an important element of service delivery responsiveness. This was evident in the higher satisfaction among patients who experienced lower waiting times during package selection, admission, and discharge processes. The connection of low waiting time and higher patient satisfaction was also observed among the beneficiaries of Rashtriya Swasthya Bima Yojana (RSBY). [31] This finding of the importance to patients of prompt attention and short waiting times is similar to observations from the Nigerian social health insurance scheme, which highlighted the three important elements as priority areas for action to improve responsiveness: autonomy in terms of the involvement of patients in decisions; communication between providers and beneficiaries; and, prompt attention with low waiting times. [7]

The high incidence and extent of OOP payments, especially in Madhya Pradesh, corresponding with low levels of patient satisfaction, is similar to other GSHIS experiences in India. [16, 32, 33] Such OOP payments in PM-JAY could be connected with the design of the scheme in terms of process flow, poor package rates, and policy of government-reserved procedures. Hospitals may be requiring such payments from patients for pre-operative diagnostic procedures due to concern that they will not receive a payment if the patient is not ultimately hospitalized. Patients in private hospitals may also be asked to make payments for certain multimodal treatments that are reserved for public facilities. This is most relevant for patients with conditions, like cancer, that requires customized treatment by a team of doctors using multiple treatment modalities. Finally, patients may be asked to pay for the difference in costs if the hospital chooses a treatment that is different from what is covered as a package reimbursement (for example, medicine or an implant for treatment of a cardiovascular condition). [34] Irrespective of the reasons, OOP payments affect financial and physical coverage and affect user satisfaction. If not addressed optimally, such payments can lead to a paradoxical situation in which an overall increase in the utilization of services results in poor financial and physical coverage. [35]

The scheme managers should streamline the existing treatment guidelines following the prevailing standard practices among the healthcare providers, but there will always be gaps between options covered by the scheme and the best options from providers' perspectives. It may be difficult for policymakers to revise existing PM-JAY packages now, in the early stages of implementation, due to the need for cost containment. However, with the expansion of coverage and utilization over time, it will be important to ensure upward revision of the existing treatment package rates to reduce OOP payments and increase patient satisfaction with PM-JAY. The benefit package rates and the restriction of certain procedures to government hospitals need to be further rationalized by streamlining existing treatment guidelines. Also, information about the list of procedures reserved for government hospitals needs to be communicated effectively to patients.

Lastly, communication among patients, healthcare providers and insurance providers remain an important element of the responsiveness of the health insurance system, as does acting upon these inputs from stakeholders. In PM-JAY, an integrated mechanism for information exchange with patients is based on the timely provision of SMSs and calls for various hospital-based transactions. However, this study found that the mechanism is performing sub-optimally, with significant room for improvement in its reach. Survey respondents' reports of low patient satisfaction due to bad provider behaviour also points to the need for a mechanism to collect such information and tie it to corrective actions.

The study has the following limitations. First, the sample size is small. In future, similar studies with a larger sample size need to be carried out to explore the phenomena from a larger pool of PM-JAY beneficiaries. Second, for this study the hospitals to shortlist PM-JAY beneficiaries were selected by the respective SHAs. This may have unknowingly introduced some biases. In future, studies may look at alternate ways of sampling the hospitals and patients.

Conclusion

This research explored the experiences of patients who used hospital services during an early phase of PM-JAY implementation. The findings provide insight into the scheme's responsiveness and success in providing financial protection. These findings highlight the importance of prompt attention and timely access to information as important attributes of patients' non-clinical expectations of health care services. Access to better information should enable PM-JAY's beneficiaries to make informed choices and seek accountability from the healthcare providers and the scheme managers, which ultimately would enhance the performance of the scheme on all three fronts of health outcomes, responsiveness and financial protection. Ensuring financial protection through reducing OOP expenses is another agenda for action by PM-JAY. This study highlighted that PM-JAY beneficiaries still face high OOP costs, through balance billing, resulting from the intricacies of provider payment mechanisms and poor governance. Lastly, the findings indicate that the policy of restricting certain procedures to public hospitals both reduces financial coverage through OOP costs and limits physical coverage and choice of provider, which are both important elements of health system responsiveness.

This research provides important insights from PM-JAY beneficiaries on their experiences of receiving treatment at hospitals under the new scheme. The results of this study emphasize that responsiveness and financial protection are inter-connected issues for a health insurance system; they also highlight several areas where changes are needed to strengthen the scheme. Because PM-JAY is a new scheme, the literature on various aspects of it remains limited. Research on the responsiveness of GSHIS schemes, in general, is also limited. This study provides new and valuable evidence in this sphere and offers policymakers options for improving the implementation of PM-JAY. Perfected implementation of this pioneering

scheme will have a major impact on the health of many of India's poorest citizens and help the country move toward achieving UHC.

Acknowledgements

We are thankful to the National Health Authority, India, for the guidance and necessary approvals for the research. We acknowledge the inputs of WHO India team members Dr Vinod Verma in conducting the research, and Dr Grace Kabaniha in improving the manuscript. We would also like to thank Dr Chandrakant Lahariya and Dr Hilde De Graeve for their support and guidance.

References

1. World Health Organization. The world health report: health systems financing: the path to universal coverage. Geneva: World Health Organization; 2010.
2. WHO. The World Health Report 2000: Health Systems : Improving Performance. Geneva: World Health Organization, 2000 9789241561983.
3. Murray CJL, Frenk J, World Health Organization. Global Programme on Evidence for Health P. A WHO framework for health system performance assessment / Christopher J. L. Murray, Julio Frenk. Geneva: World Health Organization; 1999.
4. Olmen Jv, Criel B, Bhojani U, Marchal B, Belle Sv, Chenge MF, et al. The Health System Dynamics Framework: The introduction of an analytical model for health system analysis and its application to two case-studies. 2012. 2012;2(1):21. Epub 2012-05-15. doi: 10.5195/hcs.2012.71.
5. Thi Thu Ha B, Mirzoev T, Morgan R. Patient complaints in healthcare services in Vietnam's health system. SAGE Open Medicine. 2015;3:2050312115610127. doi: 10.1177/2050312115610127. PubMed PMID: 26770804.
6. Paul S. Public Spending, Outcomes and Accountability: Citizen Report Card as a Catalyst for Public Action. Economic and Political Weekly. 2006;41(4):333-40.
7. Mohammed S, Bermejo JL, Souares A, Sauerborn R, Dong H. Assessing responsiveness of health care services within a health insurance scheme in Nigeria: users' perspectives. BMC Health Services Research. 2013;13(1):502. doi: 10.1186/1472-6963-13-502.
8. Valentine NB, de Silva A, Kawabata K. Health system responsiveness: concepts, domains and operationalization. In: Darby C, Murray CJ, Evans DB, editors. Health systems performance assessment: debates, methods and empiricism. 96. Geneva: World Health Organization; 2003. p. 573–96.
9. Carrin G, James C. Reaching universal coverage via social health insurance: key design features in the transition period2004; 13(EIP/FER/DP.04.2). Available from: https://apps.who.int/iris/handle/10665/69018.
10. WHO. Everybody's business -- strengthening health systems to improve health outcomes : WHO's framework for action. Geneva: World Health Organization; 2007.
11. United Nations. Transforming our world: the 2030 Agenda for Sustainable Development. New York, USA,: United Nations Publications; 2015.
12. Giedion U, Andrés Alfonso E, Díaz Y. The impact of universal coverage schemes in the developing world: a review of the existing evidence. Wahington DC: The World Bank; 2013.
13. Escobar ML, Griffin CC, Shaw RP. The Impact of Health Insurance in Low- and Middle-Income Countries. Wasahington DC: Brookings Institution Press; 2011.
14. Garg S, Chowdhury S, Sundararaman T. Utilisation and financial protection for hospital care under publicly funded health insurance in three states in Southern India. BMC Health Services Research. 2019;19. doi: https://doi.org/10.1186/s12913-019-4849-8.
15. Prinja S, Chauhan AS, Karan A, Kaur G, Kumar R. Impact of Publicly Financed Health Insurance Schemes on Healthcare Utilization and Financial Risk Protection in India: A Systematic Review. PLOS ONE. 2017;12(2):e0170996. doi: 10.1371/journal.pone.0170996.
16. Devadasan N, Seshadri T, Trivedi M, Criel B. Promoting universal financial protection: evidence from the Rashtriya Swasthya Bima Yojana (RSBY) in Gujarat, India. Health Research Policy and Systems. 2013;11(1):29. doi: 10.1186/1478-4505-11-29.
17. Karan A, Yip W, Mahal A. Extending health insurance to the poor in India: An impact evaluation of Rashtriya Swasthya Bima Yojana on out of pocket spending for healthcare. Social Science & Medicine. 2017;181:83-92. doi: https://doi.org/10.1016/j.socscimed.2017.03.053.
18. Mirzoev T, Kane S. What is health systems responsiveness? Review of existing knowledge and proposed conceptual framework. BMJ Global Health. 2017;2(4):e000486 doi: 10.1136/bmjgh-2017-. doi: 10.1136/bmjgh-2017-000486.
19. La Forgia G, Nagpal S. Government-Sponsored Health Insurance in India: Are You Covered? New Delhi: World Bank; 2012.

20. Peters DH, Kanjilal B. Health insurance & responsiveness to communities & patients: the future of health systems in India. Indian J Med Res. 2011;133(1):9-10. PubMed PMID: 21321414.
21. GoI. About Pradhan Mantri Jan Arogya Yojana (PM-JAY) New Delhi: National Health Authority, Ministry of Health andFamily Welfare, Government of India; 2019 [cited 2021 May 27]. Available from: https://pmjay.gov.in/about/pmjay.
22. NHA. Health Benefit Packages 2.0 User Guidelines New Delhi: National health Authority; 2020 [cited 2020 November 20]. Available from: https://pmjay.gov.in/sites/default/files/2020-10/HBP-2-0-User-Guidelines-vFinal.pdf.
23. PIB. Cost of Treatment Under Ayushman Bharat PMJAY. In: GoI MoSHaFW, editor. Delhi: Press Information Bureau, GoI; 2019.
24. NHA. Lessons Learned in one year implementation of PM-JAY New Delhi: National Health Authority; 2019 [cited 2020 7 October]. 34]. Available from: https://pmjay.gov.in/sites/default/files/2019-09/Lessons%20Learnt%20small%20version.pdf.
25. NHA. PMRSSM GUIDELINES ON PROCESSES FOR HOSPITAL TRANSACTION Delhi2018 [cited 2019 July 24]. Available from: https://www.pmjay.gov.in/sites/default/files/2018-07/GuidelinesonProcessesforHospitalTransaction_0.pdf.
26. RBI. State Finances : A Study of Budgets of 2020-21 Mumbai: Reserve Bank of India; 2020 [cited 2021 May 28]. Available from: https://rbidocs.rbi.org.in/rdocs/Publications/PDFs/0SF_271020FCF77451F1DF744B2B244875C785B8EF3.PDF.
27. National Health Systems Resource Centre. National Health Accounts Estimates for India (2016-17). New Delhi: Ministry of Health and Family Welfare, Government of India, 2019.
28. CBHI. National Health Profile 2019. New Delhi: Central Bureau of Health Intelligence, 2019.
29. Skinner CJ. Probability Proportional to Size (PPS) Sampling. In: Balakrishnan; N, Colton; T, Everitt; B, Piegorsch; W, Ruggeri; F, Teugels; JL, editors. Wiley StatsRef: Statistics Reference Online2016. p. 1-5.
30. Saluja D. KNOWLEDGE LEVELS OF RSBY BENEFICIARIES IN JHAGADIA, GUJARAT. BMJ Global Health. 2016;1(Suppl 1):A20-A1. doi: 10.1136/bmjgh-2016-EPHPabstracts.26.
31. Sihare H, Taneja P, Sihare M. Changing behavior of poor in micro health insurance Moral hazard or moral opportunity? Evidences from India. Archives: The International Journal of Medicine. 2010;3(2).
32. Sundararaman T, Muraleedharan V, Mukhopadhyay I. NSSO 71st round data on health and beyond: Questioning frameworks of analysis. 2016;51:85-8.
33. Nandi S, Schneider H, Dixit P. Hospital utilization and out of pocket expenditure in public and private sectors under the universal government health insurance scheme in Chhattisgarh State, India: Lessons for universal health coverage. PLOS ONE. 2017;12(11):e0187904. doi: 10.1371/journal.pone.0187904.
34. Trivedi M, Saxena A, Mavalankar D, Sharma M. Improving Hospital-Based Processes for Effective Implementation Of PMJAY: Evidence from Early Implementation. New Delhi: National Health Authority, 2019.
35. Gupta I, Chowdhury S, Trivedi M, Prinja S. Do health coverage schemes ensure financial protection from hospitalization expenses? Evidence from eight districts in India. Journal of Social and Economic Development. 2017;19(1):83-93. doi: 10.1007/s40847-017-0040-4.

Figure 1

Click here to access/download;Figure;S1 Fig 1.tif

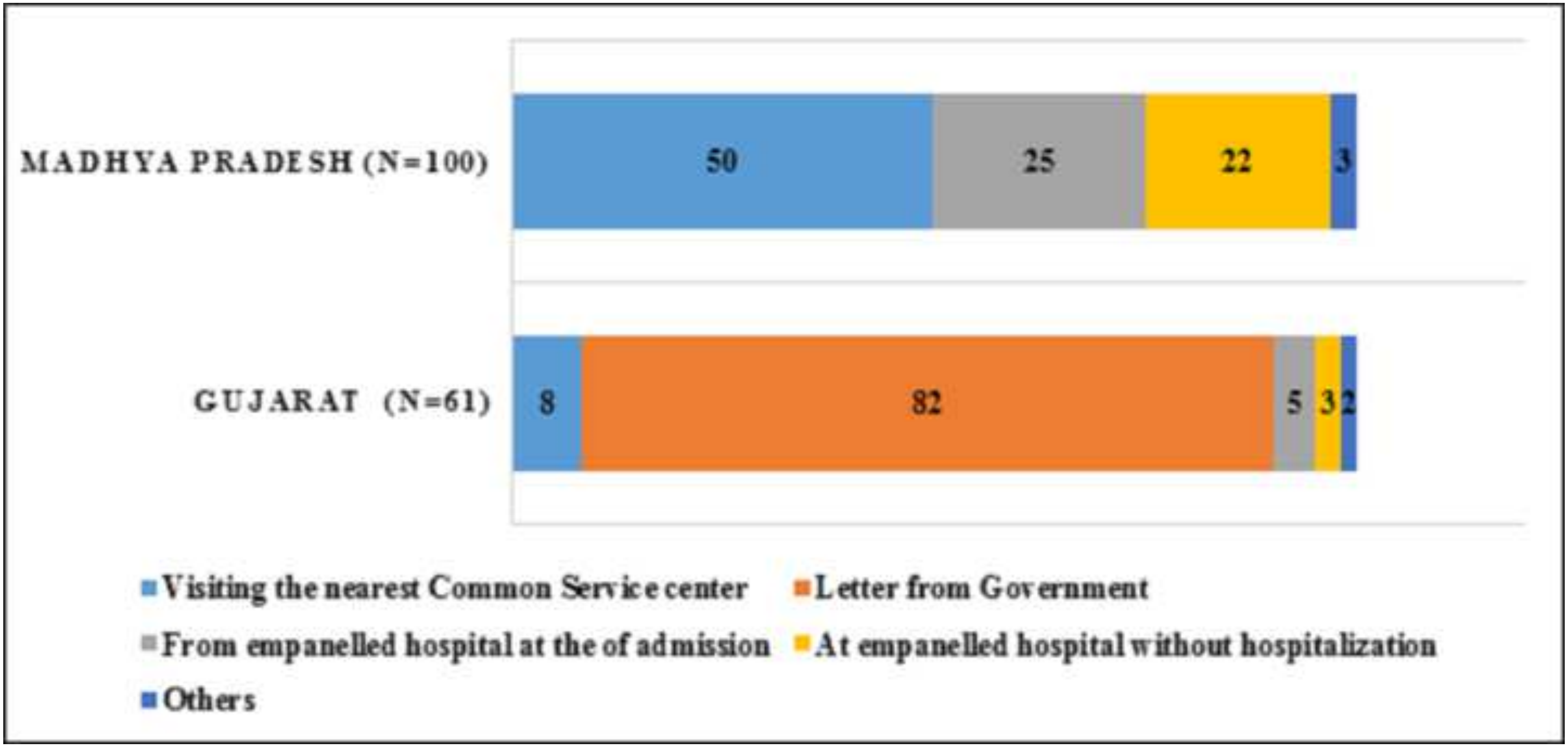

Figure 2

Click here to access/download;Figure;S2 Fig 2.tif

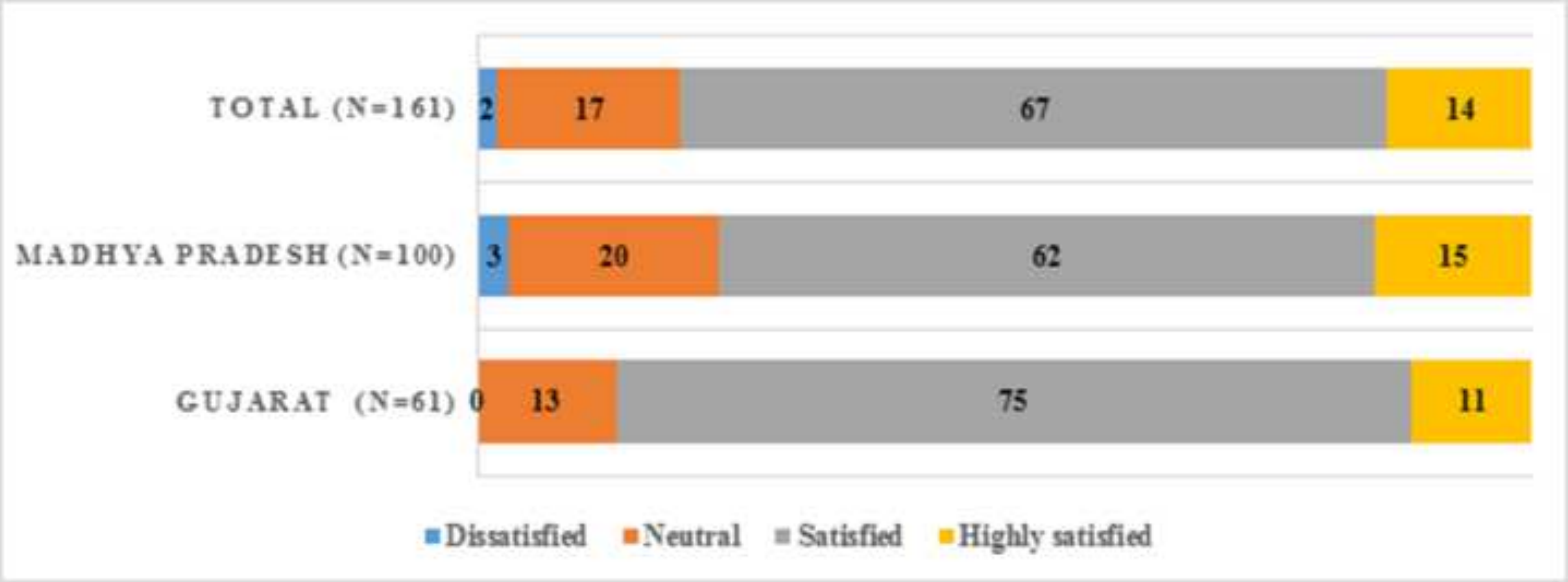

Figure 3 Click here to access/download;Figure;S3 Fig 3.tif

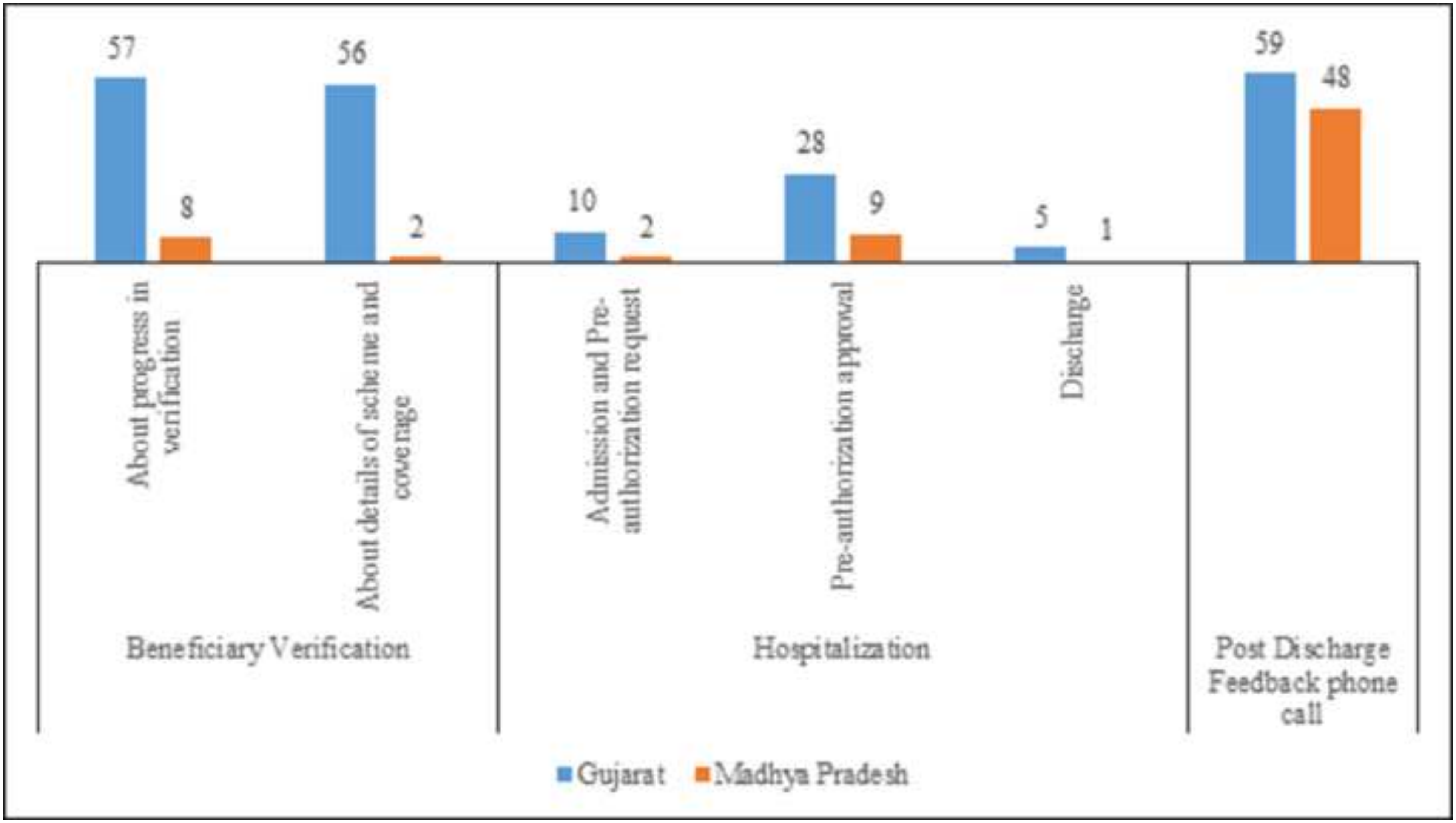

Click here to access/download
Supporting Information
S6_ Patient Survey Questionnaire_PLOS.pdf

Supporting Information Supplementary table

Click here to access/download
Supporting Information
S1 Table S1.docx

Supporting Information Figure 1 details of reasons of hospitalization

Click here to access/download
Supporting Information
S1a Fig 1.tif

Supporting Information Figure 1 details of waiting tiem for selected processes

Click here to access/download
Supporting Information
S4a Fig 4.tif

Full Title:~~Improving~~ Responsiveness and Financial Protection in a Government-Funded Health Insurance Scheme ~~in India~~: Evidence from Early Implementation of Pradhan Mantri Jan Aarogya Yojana (PM-JAY) in India ~~AB – PMJAY)~~

Short Title: Responsiveness and Financial Protection in India's PM-JAY Insurance Scheme~~PMJAY~~

Authors: Mayur Trivedi[1] *, Anurag Saxena[1], Zubin Shroff [2], Manas Sharma[1]

Author Affiliations:

[1] Indian Institute of Public Health, Gandhinagar, Gujarat, India

[2] World Health Organization, Geneva, Switzerland

* Corresponding Author: Mayur Trivedi, e-mail:

~~Email:~~mtrivedi@iiphg.org ~~(MT)~~

DECLARATIONS~~DECLARATION~~

Ethical Approval: The research was approved by the Institutional Ethics Committee at the Indian Institute of Public Health Gandhinagar, India via letter TRC-IEC No: 14/2018-19.

Availability of data and materials: This data contained in this study were made available to the research team by the National Health Authority, India for exclusive use in this study. The data belong to the National Health Authority. For more information please contact Dr Ruchira Agrawal , Consultant M&R, National Health Authority at Mel.cons1@nha.gov.in ."

~~**Availability of data and materials**: The datasets used and/or analysed during the current study are available from the corresponding author on reasonable request.~~

Competing interests: The authors~~author(s)~~ declare~~(s)~~ that they have no competing interests.

Formatted: Font: Not Bold
Formatted: Font: Bold
Formatted: Left
Formatted: Font: Bold
Formatted: Font: Not Bold
Formatted: Font: Bold
Formatted: Font: Not Bold
Formatted: Font: Not Bold
Formatted: Font: 12 pt, Not Bold
Formatted: Font: 12 pt, Not Bold
Formatted: Font: Not Bold
Formatted: Left, Line spacing: 1.5 lines
Formatted: Normal, Left, Line spacing: 1.5 lines
Formatted: Font: (Default) +Body (Calibri), Not Bold

Abstract

Introduction: Improving ~~Improvement in~~ health outcomes, assuring responsiveness and providing financial protection should be the key~~remain~~ priorities of ~~the~~ health systems~~system, in general,~~ and health insurance systems.~~system, in particular.~~ This study explored the~~research work aimed to understand the hospitalization~~ experiences of beneficiaries of Pradhan Mantri Jan Aarogya Yojana (PM-JAY), a government~~Government~~-sponsored health insurance scheme, in two states in India. The research asked recently hospitalized patients about their experiences~~It focused on identifying the enablers and barriers~~ of healthcare utilization, perceptions and overall~~perceived~~ satisfaction with the care they received~~of patients~~, and the~~their~~ access they had to information from PM-JAY~~for successful implementation of the scheme~~.

Methods~~Method~~: Data ~~for this study~~ were obtained through a ~~household~~ survey conducted with ~~of~~ 200 PM-JAY beneficiaries (or their caregivers) in the Indian states of ~~from~~ Gujarat and Madhya Pradesh.~~ (MP) was conducted.~~ The study population was comprised of~~included~~ patients~~,~~ who received healthcare~~availed~~ services at~~from the~~ 14 study hospitals in April 2019. Data collection was done using a structured questionnaire and analyzed using Stata. ~~. The study was carried out from March 2019 to August 2019.~~

Results: Overall, ~~This research provided evidence of experiences of the beneficiary's interaction with providers, processes, institutions, and information is central to the responsiveness of PM-JAY. It indicated that the~~ patients generally reported feeling~~are~~ satisfied with their experiences of PM-JAY's coverage of their hospitalization, although satisfaction varied depending~~the implementation of PM-JAY scheme, in general. It also provided evidence~~ on the state, the type of hospital they went to, and other factors. Patients'~~mean~~ out-of-pocket expenses averaged 27,648 Indian rupees (US$ 398)~~expense of Rs. 27648 during hospitalization~~ during hospitalization. Out-of-pocket expenditures, waiting times, and other factors influenced the degree of~~This influences~~ financial protection and ~~physical~~ coverage~~ and ultimately affects user satisfaction. With few~~ patients experienced. Despite PM-JAY's protocols, less than half~~indicating the receipt~~ of patients indicated receiving either text messages or~~SMSs and~~ calls regarding~~for~~ various hospital-based transactions, indicating a particular~~the findings indicates the~~ need for ~~improvement in~~ the scheme to improve its~~provision of~~ communication with patients~~ for better responsiveness~~.

Formatted: Font: 12 pt

Formatted: Heading 1

Conclusion: PM-JAY is a major new government-sponsored health insurance scheme in India. This studyresearch provides insights from itsthe beneficiaries based on their experiencesexperience of hospitalization under PM-JAY. It emphasizes both the importance and emphasized the interconnectednessinter-connected issues of responsiveness and financial protection for ain the health insurance system. This Among the very few research on the responsiveness of GSHIS schemes, this research provides valuable new evidence about the earlyand offers avenues of improvement in the implementation of PM-JAY and provides some concrete areas for improving its responsiveness and financial protection for beneficiaries. .

Keywords: Health System Responsiveness, PM-JAY, India, Financial Protection, Universal Health Coverage, Ayushman Bharat, Health Insurance

Introduction

An important goal of the health system of any country is to improve the health outcomes of its citizens. ~~-~~However, even a health system that successfully attains~~despite attaining~~ better health outcomes can be deemed unsuccessful if it fails~~, people may remain unhappy with the health system due either~~ to please users. Users can disapprove of a health system if ~~high expenses of~~ treatment costs are too high or if they perceive a ~~the~~ lack of responsiveness to their felt needs. Health~~a responsive health system. To that end, the health~~ systems must therefore~~across the world~~ also strive to provide financial protection ~~against the costs of ill health,~~ and to respond to the~~people's~~ expectations of the population as it works to preserve, promote~~, while preserving, promoting~~, and improve~~improving~~ their health. [1]

Health system responsiveness is defined as "the ability of the health system to meet the population's legitimate expectations regarding their interaction with the health system, apart from expectations for improvements in health or wealth."~~".~~ [2] Responsiveness includes both ~~The responsiveness is about the provision of~~ physical and affective support during ~~the~~ treatment. ~~-~~It incorporates~~includes~~ elements of respect for ~~people~~ and ~~client~~ orientation to the rights of clients, including ~~elements like~~ confidentiality, prompt attention, choice of providers, and the quality of amenities. The~~, etc. Also, the~~ flow and clarity of information between the health system and its~~the~~ patients also form an important element of responsiveness.~~, owing largely to information asymmetries in healthcare.~~ [3] Information asymmetries between patients and ~~While the information from the~~ providers are typical in healthcare—however, a good health system provides sufficient information to ~~the~~ patients ~~enables the latter~~ to enable them to make informed decisions on~~decide~~ the course of ~~action and appreciate the~~ treatment or other actions recommended by providers. Health systems should also have mechanisms to solicit~~, the~~ feedback from~~of~~ patients in order to gather~~forms important~~ information for

strengthening service provision. –In addition,the context of government programs can use, feedback from users to services providers and the government has been documented to be a useful tool to inform policy reforms. [4-6]

Much of the published While access to information is an important aspect of healthcare provision in general, the information imbalance gains more importance in the resource-constrained setting with low penetration of insurance and low level of financial and insurance literacy. [7] This is because the insurance providers – as payers – would carry informed negotiation with the healthcare providers on behalf of ill-informed insured patients to reduce unnecessary and expensive healthcare. This may ultimately lead to improved insurance literacy among the insured patients and their relatives. It is argued that households with higher financial and insurance literacy not only demand more insurance, they also tend to appreciate the terms and conditions of the contract. [8] While the discourse around health system responsiveness to the experiencesin terms of experience and expectations of patients isare limited to healthcare provisions perspectives fromin the developed countrycountries settings. Only a few, certain studies have explored it in the context of social health insurance schemes in developing countries. [9-11] Responsiveness is especially essential for government-sponsored health insurance schemes (GSHIS) that seek to ensure citizens' coverage by healthcare service delivery through improved strategic purchasing of healthcare services. [2, 10] GSHIS have been advocated as a means for governments to fulfil their responsibilities to citizens, including their commitments to move towards the Universal Health Coverage (UHC) envisioned under target 3.8 of the United Nation's Sustainable Development Goals (SDGs). [11]

In the past two decades, many low- and middle-income countries have introduced GSHIS of different scales and scopes. While many of these schemes have been effective in improving access and utilization of secondary and tertiary healthcare services [12, 13], there is evidence

that out-of-pocket (OOP) expenses lead to dissatisfaction among the beneficiaries [14-17]. Beyond financial protections, GSHIS responsiveness also needs to be improved through cultivating better interactions of beneficiaries with providers, processes, and institutional arrangements. [18] ~~Although not studied much, the responsiveness is essentially important in government sponsored health insurance schemes (GSHIS) that ensure the financial and physical coverage of healthcare service delivery through improved strategic purchasing of healthcare services on behalf of the citizens. [2, 12] The GSHIS have been advocated as a means to move towards Universal Health Coverage (UHC) envisioned in the form of the target 3.8 of the United Nation's Sustainable Development Goals (SDGs). [13] In the past two decades, many low and middle-income countries, including India, have introduced GSHIS of different scale and scopes. While many of these schemes are effective in improving access and utilization of secondary and tertiary healthcare services [14, 15], there has been evidence of out of pocket expenses (OOP) leading to dissatisfaction among the beneficiaries [16-19]. In addition to improving financial protections, it is important to address the responsiveness of GSHIS through better interactions of beneficiaries with providers, processes, and institutional arrangements. [20]~~

India is one of the countries that has been rapidly expanding health insurance coverage. The country has experience with both state-level and pan-Indian GSHIS. [19] These efforts culminated in 2018 in the launch of a nationwide GSHIS called Pradhan Mantri Jan Aarogya Yojana (PM-JAY). For successful implementation, this nationwide insurance scheme needs to cultivate its responsiveness to enrollees by considering enablers of and barriers to healthcare utilization, patients' perceptions of satisfaction, and access to information. [20] ~~India had a history of state level as well as pan-Indian GSHIS for a while now with the considerable expansion of coverage. [21] These efforts of more than a decade have culminated into the launch of a nationwide GSHIS i.e. Pradhan Mantri Jan Aarogya Yojana (PM-JAY). In the~~

context, India needs to consider the enablers and barriers of healthcare utilization, perceived satisfaction of patients, and their access to information for successful implementation of PM-JAY. [22]

About PM-JAY

The PM-JAY, the GSHIS launched by the Government of India launched PM-JAY in September 2018, to provide insurance coverage to approximatelyaims to cover around 500 million beneficiaries from thearound 100 million poor and vulnerable families that form the bottom 40% of the Indian population. [21] [23] The scheme is being rolled out in active partnership with the states. InWhile PMJAY eventually subsumed existing health insurance schemes in many states with a history of implementing GSHIS, PM-JAY subsumed existing health insurance schemes, such as the (for example, Mukhyamantri Amrutam scheme in Gujarat. In), it was launched as a new scheme in states where there was no pre-existing scheme, such as (for example, Madhya Pradesh, PM-JAY was launched as a new scheme.). The state governments are implementing the scheme through a trust or an insurance company, that empanels the hospitals, approves the pre-authorization process, and makes payment to the hospitals.

The scheme provides coverage of up to INR 500,000 (USD 6800) per family per year to cover a package of the expenses for medicines and diagnostics, including for selected 1393 different surgery, medical and outpatientdaycare treatments at public hospitals and empaneledempanelled private hospitals. [22] Some of these procedures, deemed prone to fraud and abuse, are reserved for public hospitals only. Beneficiaries must The expenses for these treatment/procedures are covered under PM-JAY only when it is availed in a public hospital, and the beneficiaries need to bear the expenses for these procedures ifif the treatment is availed in a private hospital. [23][24]

PM-JAY has established~~involves~~ certain touchpoints for beneficiaries' interactions~~interaction~~ with the health insurance system when seeking ~~from the time a beneficiary arrives at the hospital for~~ treatment. Guidelines are ~~to discharge. Accordingly,~~ clearly defined ~~guidelines have been developed~~ for each non-clinical step of the hospitalization process: ~~viz. a)~~ registration, ~~b)~~ selection of treatment package before hospitalization, ~~c)~~ preauthorization and admission during hospitalization, ~~d)~~ discharge and payments, and ~~e)~~ information exchange at various touchpoints. [24] To support beneficiaries' engagement with PM-JAY, its guidelines state that all empaneled hospitals must place ~~[25]~~

~~The guidelines state that empanelled hospital need to ensure~~ a dedicated ~~PM-JAY~~ helpdesk in ~~for PM-JAY at~~ a prominent location.~~place in the hospital.~~ The helpdesk is to be managed by an~~the~~ Ayushman Mitra (AM), a representative who manages ~~the~~ operations~~,~~ and liaises between~~liaison with~~ patients, doctors, hospital administration, and the scheme managers. The primary purpose of the helpdesk is to provide 'one-stop'~~stop~~ services during~~regarding~~ registration, hospitalization, and discharge. The PM-JAY guidelines state that ~~based on~~ the AM should be able to use a diagnosis sheet provided by the doctor to select~~, the AM/Computer operator should be able to block~~ the treatment package(s) in the~~using~~ PM-JAY IT system. Once the package is selected and blocked, the~~a~~ request is submitted to~~received by~~ the insurance company/trust for pre-authorization. According to guidelines, this ~~the guideline, the~~ request must~~has to~~ be either approved or rejected within six hours. When the patient is discharged from the hospital, ~~According to the guidelines for discharge, that~~ the AM similarly completes an~~/computer operator should be able to fill the~~ online discharge summary form. The~~Lastly, the~~ patient receives~~should be provided with~~ a discharge summary from the hospital. Being a cashless scheme, patients are not supposed to make any payments during any stage of hospitalization. The patient is also expected to receive text messages via short message service

(SMS) and calls regarding these processes from the scheme authorities.at the time of the discharge. It does not mention the time duration to complete this process. [25][26]

Rationale of the study

PM-JAY was designedis expected to beemerge as an effective mechanism for ensuring health insurance coverage to a large number of economically poor Indian citizens. This population has previously been considered to beIndians, who remain passive recipients of servicesthe healthcare care from the public and private sector healthcare, and neither sector has been especially responsive to their preferences. Policymakers currently lack sufficient knowledge about beneficiaries' experiences of navigating hospitalization processes and accessing information. . While health system responsiveness depends on a range of factors, within the health system, the beneficiaries' expectations and experiences experience of availing health coverage can also provide useful insight, bothinsights in the early phase of implementation as well as. However, there remains a gap in knowledge about the experiences of beneficiary interactions in navigating these hospitalization processes, and in receipt of the timely and useful information. Insights from early implementation about patients' experience of availing treatment and coverage benefits in the light of what is envisaged in the guideline can help policymakers and programme planners for timely and adequate midcourse corrections. [7] Tracking the [9] The responsiveness of the health insurance scheme like PM-JAY (and other insurance schemes) iscan also be an important element of monitoring and evaluation. of important health insurance system.

In this context, this research aims to understand the experiences of beneficiaries who utilizedhave availed services under PM-JAY at empaneledfrom empanelled public and private hospitals. Specific areas of interest These include their experiencesa) experience of: beneficiary identification and authentication;, b) experience of hospitalization and scope of services

received, including~~ (e.g. free~~ medicines;~~), c)~~ information exchange during beneficiary verification,~~ (e.g. progress in verification, and details of scheme and coverage to a verified beneficiary),~~ before hospitalization,~~ (e.g. receipt of SMS informing them about details of packages and balance),~~ during discharge, and ~~(e.g. receipt of discharge summary and copies of diagnostic reports, and SMS), and~~ after hospitalization; and,~~ (e.g. follow up call from the designated call centre to ask about the hospitalization experience), and d)~~ perception of overall quality of services and any~~, including~~ additional payments made to the hospital separate from~~, over and above~~ the cashless payments by~~payment provided to hospitals under~~ PM-JAY.

By documenting~~aiming to understand~~ the experiences of PM-JAY beneficiaries, this study seeks to enrich~~enriches~~ the existing ~~PM-JAY~~ literature on PM-JAY responsiveness~~base~~ and provide~~provides~~ actionable insights to strengthen the scheme.

Methodology

This~~The~~ study was conducted between~~during~~ March and ~~-~~August 2019. In consultation with the National Health Authority (NHA),~~Data for this study were obtained through a household survey of beneficiaries.~~ Gujarat and Madhya Pradesh ~~(MP)~~ were selected as the study locations. Gujarat is an economically developed state with a higher annual per capita Gross State Domestic Product (GSDP), and a higher level of health expenditure (Table 1). Study~~, in consultation with the NHA. The study~~ sites included seven hospitals in~~from~~ each state; these were~~,~~ selected by the respective State Health Agency (SHA) after ensuring representation of a) public and private hospitals, and b) multi-speciality and super-speciality hospitals. As shown in~~can be seen from~~ Table 2~~1~~, the ~~sampled~~ fourteen hospitals included in the study comprised nine multi-speciality and five super-speciality hospitals. Eight of~~Of~~ the ~~fourteen~~ hospitals~~, eight~~ were private ~~hospitals~~ and the rest were public hospitals.~~, two of which were autonomous hospitals.~~

Table 1: Comparison of study states across selected expenditure parameters			
State		Gujarat	Madhya Pradesh
Gross State Domestic Product (GSDP) per capita (INR)*		224896	99025
State health expenditure (2019-20 Budget Estimate)*	Per capita (INR)	1610	1284
	Percent of GSDP	0.7	1.3
	Percent of total revenue expenditure	7.1	5.9
Per capita health expenditure (INR) (2015-16)**	Total	3703	2820
	Government	1429	811
	OOP	1781	1944
Average total expenditure on account of hospitalization per hospitalization case (INR) (2014)***	Rural	32503	19385
	Urban	26401	17117
Sources: * State Finances: A Study of Budgets of 2020-21, Reserve Bank of India [26]; ** National Health Accounts India (2015-16) [27] *** National Health Profile 2019 [28]			

The study population was drawn from ~~included~~ patients who were hospitalized in April 2019. Sample~~ The sample~~ selection was ~~to be~~ done using~~from~~ the list of all claims from study hospitals, as provided by the SHAs in ~~respective SHA of~~ the two ~~study~~ states. Each~~ Towards this end, the~~ claims list was ~~first~~ converted into a patient list by retaining one claim per ~~patient, as needed in case of multiple claims filed for one~~ beneficiary. ~~-~~Next, to ensure ~~the hospital-level~~ representation of most and least popular services at each hospital, the ~~, it was decided to select~~ most ~~and least~~ popular specialties were selected.~~clusters and least popular clusters of specialty from each hospital.~~ For each hospital,~~of the hospitals, the~~ specialty clusters were arranged from those having the highest number of patients to those having the lowest. Those clusters that together contained the ~~number of patients. From this arrangement, starting from the~~ top~~, those clusters that together contain~~ 10% of ~~the~~ patients were identified as the 'most popular cluster'~~cluster~~ for that ~~particular~~ hospital. Similarly, ~~starting from the bottom,~~ those clusters that together contained the bottom~~contain~~ 10% of ~~the~~ patients were identified as the 'least popular cluster'~~cluster~~ for that ~~particular~~ hospital. The resulting~~This~~ list of patients in~~,~~

who availed the hospitalization for ‘top - -bottom’ clusters, was then used to generate a sample of 100 beneficiaries in each state through the probability proportional to size (PPS) method using thestudy hospital as the sampling unit. Thus a This meant that higher proportion of the sample was drawn fromallocated to the hospital with the mostmore number of claims and the smallest sample was derived from the hospital with the fewestleast number of claims.- The use of a multi-stage sampling method with the use of PPS ensured that the selection of respondents was not biased either in favour or against any type of specialty and hospital. [29] The final distribution of patients included in the studythat were surveyed across the different types of hospitals is shownean be found in Table 2. The1. These sampled patients were from six districts in Gujarat and five districts in Madhya Pradesh. Among the From among 100 patients inof Gujarat, 39 were only enrolled only in the Mukhyamantri Amrutamthen existing critical illness coverage i.e. Mukhyamantri Amrutam (MA) scheme that existed at the time, not the full PM-JAY scheme (which has since subsumed Mukhyamantri Amrutam). .

Table 21: Study hospitals and surveyedsample beneficiaries,: a comparison by statestates

Hospital type	Ownership type	Gujarat		Madhya Pradesh	
		Number of hospitals	Sample of beneficiariespatients	Number of hospitals	Sample of beneficiariespatients
Multi-specialty	Private	2	35	2	29
	Public	2	21	3	57
Super-specialty	Private	2	21	2	14
	Public	1	23	-	-
Grand Total		7	100	7	100

Data for this study were obtained through a household survey of the sampled beneficiaries. The data collection was done during May-June 2019. A structured questionnaire was developed to solicitinclude details of the patient’s hospitalization experience keeping in consideration, following the guidelines fromof PM-JAY. —The draft questionnaire was reviewed and approved by the technical experts at WHO and the NHA. Before data collection

Formatted: Font: 10 pt
Formatted: Font: 10 pt
Formatted: Font: 10 pt
Formatted: Font: 10 pt
Formatted: Font: 10 pt
Formatted: Font: 10 pt
Formatted: Font: 10 pt
Formatted: Font: 10 pt
Formatted: Font: 10 pt
Formatted: Font: 10 pt, Not Bold
Formatted: Font: 10 pt
Formatted: Font: 10 pt
Formatted: Font: 10 pt, Not Bold
Formatted: Right
Formatted: Font: 10 pt

commenced, the The revised instrument was then piloted for accuracy and validation through, before commencing the data collection. This included a discussion with a panel of in-house experts and a field test. The survey questionnaire is available as supplementary material to this paper. The data collection was done by a team testing of six investigators in May and June 2019. Before going to the field, these investigators underwent a one-day training specifically designed for the data collection under this research. The questions were asked in the local language i.e. Gujarati for Gujarat and Hindi for Madhya Pradesh. Each survey administration lasted for 30-40 minutes.instrument.

The survey was administered to - either the patient patients or his/her primarytheir main caregiver -relatives were survey respondents and were interviewed at their residences. They were contacted telephonically in advance to ascertain their availability for the survey. All participants were provided with a participant information sheet that described the ethical concerns and emphasized the rightsplace of the respondents.residence. After providing a brief description of the study and its objectives, surveyors requested the respondent's participationrequest to participate in the survey was made and verbal non-witnessed informed consent was taken. Nine ofsought from the initially contacted respondents refused to participate in the survey. The consented. The respondents were asked to indicate the reasons for and duration of their hospitalization. -They were also asked in detail about their experiences and any challenges experienced duringaround beneficiary identification and authentication, the experience of hospitalization and the scope of services received (includinge.g. free medicines). The responsiveness was measured in the form of a) effectiveness of helpdesk, b) time taken at different stages of hospitalization and discharge events, and c) information exchange between patient and scheme authorities.To ascertain the overall quality of services, the respondents were asked to rate their experience of registration and hospitalization on a five-point Likert scale that ranged from 'highly dissatisfied' to 'highly satisfied'. To ascertain the effectiveness of

the helpdesk services~~ at the hospitals~~, respondents were asked ~~in ~~closed-ended questions about whether~~if~~ they had received three types of help from the registration desk: 1~~ viz. a~~) information about PM-JAY; 2~~, b~~) help with documents and computerized registration; and, 3~~ c~~) guidance about treatment within the hospital. Respondents were asked to provide ~~ As a part of the patient survey, the respondents were asked to report how much time did the patient/ relatives spent during admission and pre-authorization request. To ascertain the extent of out-of-pocket payment (OOP), the respondents were asked to provide information on the additional payments made to the hospital over and above the cashless payment provided to hospitals under PMJAY. Lastly, the respondents were asked to provide ~~details of any message~~SMS~~ and telephone calls ~~that patient/relative may have ~~received from the scheme authorities about registration, preauthorization, and discharge. Respondents were also asked to report how long the admission, pre-authorization request and discharge processes took. The financial protection was measured in terms of OOP payment by the patients. To ascertain the extent of OOP payments, respondents were asked to provide information on any additional payments made to the hospital separate from the cashless payment provided to hospitals by PMJAY. The quality of services was measured in the form of patient satisfaction. To ascertain the overall quality of services, respondents were asked to rate their experiences of registration and hospitalization on a five-point Likert scale that ranged from ‘highly dissatisfied’ to ‘highly satisfied’.

The~~ structured~~ questionnaire for the household survey was configured using~~in the~~ Open Data Kit (ODK) software, a free and open-source tool developed by the University of Washington. The data ~~data ~~were collected using ~~a ~~hand-held electronic devices~~device~~. On-site monitoring and cross-verification of data collection were carried out to ascertain the reliability of the data being collected. This~~ The data~~ was done by the research supervisors through observation and spot-checking. The data were cleaned using MS-Excel software and ~~analyzed to generate~~ descriptive statistics were generated using STATA (Version~~software version~~ 12), developed

by StataCorp LLC.~~.~~ The study received ethical clearance from the institutional ethics committee of the Indian Institute of Public Health Gandhinagar (IIPHG) (~~vide letter~~ TRC-IEC No: 14/2018-19).~~.~~

Results ~~and Findings~~

The results ~~and findings~~ of the study are presented in six ~~divided across five sub-~~sections. The first section provides basic sociodemographic information on the 200 survey respondents, as well as an overview of the health care services they received during their hospital visits and their overall satisfaction. The~~,~~ following sections are organized based on the beneficiaries' ~~the experiences of the beneficiary~~ interactions with aspects of the PM-JAY system: identification and ~~viz. a)~~ registration;~~, b)~~ treatment package selection before hospitalization;~~, c) preauthorization and admission during hospitalization, d)~~ discharge and payments;~~,~~ and~~, e)~~ information exchange.

Respondents' characteristics and services received

Table 3 presents ~~Before presenting the results for the experiences of interactions,~~ a summary of the socio-demographic profile of the survey respondents. With a high proportion of respondents having a low level of education, belonging to socially marginalized sections of scheduled caste and other backward castes, and fewer having a salaried job, it indicates that the PM-JAY is reaching the socio-economically poorer section of the society. ~~household survey is presented below as Table 2.~~

~~.~~

Table 3~~2~~: Profile of the surveyed~~sample~~ beneficiaries ~~(In percentage)~~

Particulars	Details	Gujarat (n=100)	Madhya Pradesh (n=100)	Total (%) ~~(n=200)~~
Type of hospital~~hospitals~~	Private	56	43	49.5
	Public	44	57	50.5
Gender	Female	38	53	45.5

Formatted Table

	Male	62	47	54.5
Mean age (years~~Age (year~~)		49.1 (Range 6-79)	42.2 (Range 2-75)	
Religion	Hindu	89	89	89
	Muslim	11	11	11
Caste	Scheduled Tribe	5	0	2.5
	Scheduled Caste	20	5	12.5
	Other Backward Caste	49	69	59
	Other~~Others~~ (General)	26	25	25.5
	Refuse to answer	0	1	0.5
Highest level of education attained in the household	Illiterate or no formal education	39	15	27
	Primary education (1-8 standard)	35	50	42.5
	Secondary education (9-12 standard and diploma etc.)	23	26	24.5
	Class 12 and above	3	9	6
Occupation	Farm Labor~~Labour~~	18	4	11
	Other labor~~labour~~ work in a rural area	8	7	7.5
	Labor~~Labour~~ work in an urban area	14	22	18
	Self-employment (agricultural ~~work~~)	20	7	13.5
	Self-employment (non-agricultural)	19	38	28.5
	Salaried job	19	22	20.5
	Other~~Others~~	2	0	1
Respondents~~The proportion of respondents~~ who received benefits under state-sponsored health coverage before PMJAY		75	3	39

Formatted Table

In terms of~~Of~~ the services received by the beneficiaries~~200 respondents~~, 39% had hospitalization ~~'Hospitalization~~ with surgery~~surgery'~~, 37% reported hospitalization~~'Hospitalization~~ without surgery~~surgery'~~, and the remaining ~~one fourth~~ respondents received daycare treatment as an outpatient without having to remain overnight at the hospital. These daycare treatments included~~like~~ dialysis or ~~sessions of~~ cancer treatment sessions.~~-~~ These proportions varied~~were different~~ across states and type~~ownership~~ of hospital.~~hospitals.~~ Among the patients who utilized~~availed~~ services in public hospitals in~~of~~ Gujarat, 61% received ~~the proportion of~~ day-care procedures. ~~was 61%.~~ In Madhya Pradesh, ~~a higher proportion of respondents (~~61% of respondents~~%)~~ from ~~the~~ public hospitals reported receiving hospitalization without surgery. In both states, around ~~Around~~ half of the respondents who received~~availed~~ treatment in private hospitals had~~did so for~~ surgical procedures. ~~in both the states. The details can be better understood through Figure 1 below.~~

~~Figure 1 Proportion of beneficiaries by reasons for hospitalization: Comparison across the type of hospitals and state~~

The average length of stay for hospitalizations~~hospitalization~~, excluding day care procedures, was 3.5 days for Gujarat and 6.1 days for Madhya Pradesh. Stays were longer~~This was higher~~ for surgical hospitalization (6.6 days) than for~~), followed by~~ non-surgical hospitalization (5.6 days). The~~ Similarly, the~~ average length of hospitalization was 5.1 days in public hospitals and 4 days in~~for~~ private hospitals. There was little~~ and 5.1 days for public hospitals. While there was not much~~ difference between the~~across~~ two types of hospitals in Madhya Pradesh: ~~(~~6.1 days for private and 6.2 days for public hospitals. Private~~), private~~ hospitals in Gujarat, however, had noticeably shorter stays (~~a lower length of stay of~~ 2.3 days) than ~~, as compared to around 3.9 days in~~ public hospitals (3.9 days). ~~of Gujarat. With this background, the experiences of respondents along the various touchpoints of hospitalization are presented below.~~

~~A.~~ Beneficiary identification and registration

The first touchpoint of the patient experience that was examined was beneficiary identification and registration. This involves beneficiaries' awareness of ~~about the scheme and~~ the insurance scheme and their experiences using~~experience of availing~~ the e-card indicating ~~beneficiary~~ registration in the scheme. The number~~ From among 100 patients~~ of respondents from Gujarat in this section is 61, as at the time~~,~~ 39 were enrolled in the ~~only in the then existing critical illness coverage i.e.~~ Mukhyamantri Amrutam ~~(MA)~~ scheme, which had not~~and were~~ yet been~~to be~~ subsumed under PMJAY; these~~. These~~ patients did not have a comparable registration experience.

Formatted: Font: Times New Roman, 12 pt

Formatted: Heading 2, Left, Space Before: 0 pt, Line spacing: single, No bullets or numbering

Formatted: Font: 12 pt

Formatted: Font: 12 pt

Formatted: Font: 12 pt

Formatted: Font: 12 pt

Formatted: Font: 12 pt

Formatted: Font: 12 pt

Formatted: Font: 12 pt

Formatted: Font: 12 pt

Formatted: Font: 12 pt

Therefore, the 'n' for Gujarat in this section is 61. A large proportion (74%) of PM-JAY beneficiaries in Gujarat (74%) indicated that they were made aware ofgot to know about the scheme throughas they received a letter from the government indicating their entitlement. Another 11% indicated that they got to know about the scheme through village level health workers and 7% got to know about the scheme only at the time of the hospitalization. The remaining respondents knew about the scheme from friends and relatives or through newspapers. None of the respondents from Madhya Pradesh reported receiving any In MP, no such letter. was reported by any respondent. Instead, nearlyaround one-third of the beneficiaries (30%) learnedgot to know about the scheme when they arrived at the hospital, whilevisited the hospitals for hospitalization, and another one-quarter of thefourth respondents got to know about the scheme from their friends and relatives. The remaining sources included newspaper and internet, etc.

Most (82%)As far as the checking of eligibility is concerned, most beneficiaries from Gujarat (82%) mentioned that the letter from the government indicated their eligibility for entitlements in the scheme as well. In Madhya Pradesh, around half of the respondents indicated that they checked their eligibility at the common service centers, government-authorized one-stop-facility for availing digital services on various public schemes and entitlements.centres (Figure 1). 2). This proportion was 63% among those usingbeneficiaries from the private hospitals in Madhya Pradesh;, as around one-third of beneficiaries usingfrom public hospitals only checked their eligibility at the hospital at the time of the admission at the hospital.

Figure 12: Proportion of beneficiaries by the source of eligibility checking: a comparison across states (In %)

As mentioned, survey respondents were asked specifically about whether they received help with three aspects~~for the effectiveness of the helpdesk services~~ at the PM-JAY helpdesks. Half~~hospitals, half~~ of the beneficiaries (52%) reported receiving ~~to have received~~ information about PM-JAY, help with documents and computerized registration, and guidance about treatment within the hospital. ~~-~~The proportion of respondents who received all three kinds of help was highest in the public hospitals of Gujarat (82%), and lowest in the private hospitals of Madhya Pradesh (35%). ~~-~~Only 7% of respondents~~, mainly from a public hospital in Madhya Pradesh,~~ indicated that they did not receive any help at the helpdesk—these were respondents who had been at a public hospital in Madhya Pradesh. Fewer~~. Less~~ than one in ten beneficiaries reported facing problems with ~~that they faced problem in~~ registration~~,~~ in the form of long waiting time or payment before registration.~~.~~

As shown in ~~can be seen from~~ Figure 2~~3~~, 81% of respondents felt~~were~~ either ‘satisfied’ or ‘highly satisfied’ with the registration process. This was higher in Gujarat (86%) than in~~as compared to~~ Madhya Pradesh (77%), and among beneficiaries from public sector hospitals (83%) than their counterparts from the private sector (77%).

Figure 2~~3~~: Proportion of beneficiaries by their level of satisfaction with registration: a comparison across states (In %)

~~B.~~ Treatment package~~Package~~ selection and blocking

PMJAY guidelines state that once the patient’ eligibility is ascertained, the AM should be able to block the benefit package(s) efficiently using the PMJAY IT system. The self-reported average time for ‘admission and pre-authorization request’ – i.e. time that the AM took in preparing and submitting the request - was 30 minutes~~half an hour~~ in Gujarat and three hours in Madhya Pradesh. In Madhya Pradesh, the~~This~~ duration was approximately~~around~~ one hour

Formatted: Heading 2, Left, Space Before: 0 pt, Line spacing: single, No bullets or numbering

Formatted: Font: Times New Roman, 12 pt, Bold

for the patients at~~who availed treatment in~~ public hospitals and more than five hours for those who were hospitalized in private hospitals.~~ of Madhya Pradesh.~~ The average time for the ‘pre-authorization approval’ to be received from the scheme authorities~~approval~~ was four hours for the patients from Gujarat and nine and a half hours for patients from MP. Patients in~~ While the patients who availed hospitalization from~~ the public hospitals of MP reported a mean time of 1.7 hours, while the beneficiaries at~~of~~ private hospitals reported a mean time of 19.5 hours for receiving approvals.~~ (Figure 4).~~

~~**Figure 4: Average time (in hours) in selected processes under PM-JAY hospitalization**~~

~~C.~~ Discharge and ~~Out of pocket (OOP)~~ payments

According to the patients, ~~patient’s experience, the~~ hospitals in Gujarat took around one hour to complete the discharge process,~~, as~~ compared to 2.3~~three~~ hours in Madhya Pradesh. While all hospitals took a similar time for discharge in Gujarat, this was higher for private hospitals (3 hours) as compared to public hospitals (1.8 hours) in Madhya Pradesh.~~ ~~ The possibility of having to make additional payments outside of insurance coverage ~~payment~~ at the time of discharge remained~~remains~~ an important concern for the patients. As shown in ~~can be seen from~~ Table 4, 27% of~~3, around one-fourth~~ respondents ~~(27%)~~ reported that they paid OOP fees either before, during, or after hospitalization. A lower~~higher~~ proportion of respondents who reported making OOP payments~~‘zero payment’~~ were from Gujarat (11~~89%) as compared to MP (57%), and from Public hospitals (80~~%) as compared to 43% in Madhya Pradesh. A lower proportion (20%) of patients from public hospitals made OOP payment as compared to those at private hospitals (34%). The highest~~Private hospitals (66%). There was a higher~~ incidence of OOP was among patients of private hospitals in Madhya Pradesh~~MP~~.

Formatted: Font: Times New Roman, 12 pt, Bold

Formatted: Heading 2, Left, Space Before: 0 pt, Line spacing: single, No bullets or numbering

Formatted: Font: Times New Roman, 12 pt, Bold

Table 4~~3~~: Incidence of OOP payments during hospitalization (%)~~(In %)~~			
Hospital type	Gujarat	Madhya Pradesh	Total
Private	13<br>(n=56)	63<br>(n=43)	34<br>(n=99)
Public	9<br>(n=44)	28<br>(n=57)	20<br>(n=101)
Total	11<br>(n=100)	43<br>(n=100)	27<br>(n=200)

Most~~On exploring reasons for the payment, it was found that most~~ respondents who reported making OOP~~from public and private hospitals indicated that these~~ payments indicated they were for either medicines or ~~medicine and~~ diagnostic tests.~~investigations.~~ Patients were required~~needed~~ to make ~~such~~ payments directly to the pharmacies or laboratories outside the hospitals,~~pharmacy or laboratory~~ as they were told that certain ~~treatment~~ inputs were either not available or not covered under the PM-JAY. ~~-~~In addition, around one-fifth of respondents who made OOP payments,~~–~~ mostly from private hospitals in Madhya Pradesh,~~–~~ indicated that they were asked to make a lump-sum payment directly at the hospital billing counter. These patients reported that they~~They~~ were informed at the helpdesk that the actual costs of their treatments or procedures were~~cost of the procedure was~~ higher than the amount~~funds~~ the hospital would receive from ~~in the form of~~ claim reimbursements~~reimbursement~~ from PM-JAY;~~the scheme, and~~ thus, patients needed to pay the balance~~amount as part-payment~~.

In terms of the amounts~~magnitude~~ of the OOP payments~~payment~~, there were notable differences~~was a significant difference~~ across states~~state~~ and types~~type~~ of hospitals, as shown~~can be seen~~ in Table 5.~~4.~~ The four patients who had to make OOP payments while receiving~~availed~~ treatment in public hospitals in Gujarat had the lowest average amount: ~~OOP payment at~~ INR 1,550. Patients at ~~1550 (n=4) and the patients from~~ private hospitals in Madhya Pradesh ~~MP~~ paid the highest average amount of OOP: ~~(~~INR 40,996 (n=27). Patients in ~~)). The~~

Formatted: Font: 12 pt

Formatted: Font: 12 pt

Formatted: Font: 12 pt

Formatted: Font: 12 pt, Not Bold

Formatted: Font: 12 pt

Formatted: Font: 12 pt

Formatted: Font: 12 pt

Gujarat~~ patients~~, as well as public hospital patients in Madhya Pradesh~~of MP~~ who reported lower OOP payments, had to purchase ~~, spent on ~~medicine and other supplies ~~that were to be purchased ~~from outside the hospital. ~~-~~Most of the ~~high OOP ~~respondents from private hospitals of MP who made high OOP payments had ~~either ~~claimed for either cancer-related procedures or cardiovascular procedures that involved the replacement of a~~the~~ valve or pacemaker. The ~~It is important to note that the ~~average OOP payment was around three times higher for those patients who were asked by the hospitals to make a ~~'~~part-payment~~payment'~~ on top of their insurance~~the~~ coverage (mean:~~Mean expenses~~ INR 47,840; median:~~. 47840, Median expenses~~ INR. 42,500), when~~as~~ compared with~~to~~ those who reported paying OOP for medicines, ~~spending on medicine/~~tests, ~~/~~blood, or other individual costs (mean:~~ etc. (Mean expenses~~ INR 17,172; median~~. 17172 Median~~ expenses:~~ —~~ INR 3,250~~. 3250~~).

Table 5~~4:~~ Average payment during hospitalization (~~In ~~INR)

Hospital type	Gujarat	Madhya Pradesh	Total
Private	5,558~~5558~~ (n=7)	40,996~~40996~~ (n=27)	31,341~~31341~~ (n=34)
Public	1,550~~1550~~ (n=4)	5,123~~5123~~ (n=16)	4,409~~4409~~ (n=20)
Total	4,100~~4100~~ (n=11)	27,648~~27648~~ (n=43)	22,851~~22851~~ (n=54)

Overall, a significant majority of survey respondents were either ‘satisfied’ or ‘highly-satisfied’ with their hospitalization experience. This was higher in Gujarat (82%), compared with Madhya Pradesh (71%), and among public hospitals (82%), compared with private hospitals (66%) Dissatisfied respondents reported problems, including bad behaviour on the part of healthcare providers, long waiting times during preauthorization and admission, and OOP expenses.

Formatted: Font: 10 pt
Formatted: Font: 12 pt
Formatted: Font: 10 pt
Formatted: Font: 12 pt
Formatted: Font: 12 pt, Not Bold
Formatted Table
Formatted: Font: 12 pt
Formatted: Font: 12 pt
Formatted: Font: 12 pt
Formatted: Font: 12 pt
Formatted: Font: 12 pt
Formatted: Font: 12 pt
Formatted: Font: 12 pt
Formatted: Font: 12 pt
Formatted: Font: 12 pt
Formatted: Font: 12 pt
Formatted: Font: 12 pt
Formatted: Font: 12 pt

Information exchange before, during, and after hospitalization~~As per the results of the survey, three – fourth respondents were either 'satisfied' or 'highly satisfied' with their hospitalization experience. This was higher in Gujarat (82%) as compared to MP (71%). The dissatisfied respondents were those who also reported the problem in the form of a) poor quality services in the form of the bad behaviour of healthcare providers, b) long waiting time during preauthorization and admission, and c) out-of-pocket expenses.~~

~~**D. Experience of receipt of SMS during and feedback call after hospitalization**~~

Formatted: Heading 2, Left, Space Before: 0 pt, Line spacing: single, No bullets or numbering

Formatted: Font: (Default) +Headings (Calibri Light), 13 pt, Not Bold

The PM-JAY guidelines indicate that patients should receive information about various processes concerning their hospitalization through calls and SMSs, which are made by the scheme authorities.~~As can be seen from~~ Figure 3 shows that~~5, in Gujarat,~~ more than half of the patients in Gujarat reported receiving messages via SMS during the verification step;~~, while~~ lower proportions of patients reported receiving SMSs~~SMS~~ for preauthorization, admission and discharge. In Madhya Pradesh, a significantly lower proportion of patients reported receiving SMSs across processes.

Figure 3~~5~~: Proportions of beneficiaries who received SMS/call about specific processes (in %)

The most commonly reported~~common~~ information exchange was a call ~~that the beneficiaries received~~ after ~~the~~ discharge soliciting their feedback.~~–~~ Nearly half of the beneficiaries in Madhya Pradesh and 60% in Gujarat reported having received a post-discharge feedback call. This proportion was 64% among public hospital beneficiaries of Gujarat and 40% among Madhya Pradesh beneficiaries who received services from public hospitals.

Discussion

Health outcomes

~~Good health~~, responsiveness, and financial protection are three main elements of the performance of any~~the~~ health system, including a ~~in general, and~~ health insurance scheme such as PM-JAY. ~~, in particular.~~ This study~~research~~ looked at issues ~~two distinct but~~ related to~~issues of~~ responsiveness and financial protection under PM-JAY by soliciting data on beneficiaries'~~through~~ experiences with~~of the beneficiary's interaction with the~~ providers, processes, institutions, and information. The~~This~~ research provides evidence on patient~~of such~~ experiences across a range of touchpoints during hospitalizations covered by PM-JAY. It also~~in a journey of the patient while they availed hospitalization services under PM-JAY. Also, it~~ provides evidence on the degree of financial protection afforded by~~in~~ the scheme by quantifying~~form of~~ OOP expenses during hospitalizations.~~hospitalization that influences an important objective of the scheme.~~

Formatted: Font: Times New Roman, 12 pt

Overall, the study found that PM-JAY was relatively successful in both responsiveness and financial protection, although significant room for improvement on both fronts remains. The study also showed that patient experiences vary depending on what state they are in and what type~~source~~ of hospital they use.

To begin with, patients need basic information about their coverage under PM-JAY. In Gujarat, many patients had received a letter about the ~~the~~ scheme and their eligibility ~~thereof through a letter~~ before they experienced illness—this ~~, as the case was with Gujarat,~~ is a positive sign that the scheme is reaching out to beneficiaries. This is an improvement over poor information among the beneficiaries of similar GSHIS in Gujarat. [16, 30] Knowing about the nature of their coverage before the onset of an illness can~~may~~ influence patients'~~the~~ treatment-seeking behaviour. This contrasts ~~as the patients knew about the coverage before the onset of the illness. This is in contrast~~ with the less-ideal situation for most patients in Madhya Pradesh, who only

learned~~getting to know~~ about the scheme and their eligibility only at the time of illness and arrival at a hospital~~, as was the case with patients in Madhya Pradesh~~.

Interactions with~~The interactions at~~ the PM-JAY helpdesks are~~helpdesk remains~~ crucial sources~~as a source~~ of information about ~~the~~ hospitalization coverage at the time of ~~the~~ illness. Access to a helpdesk was correlated with overall patient satisfaction:~~ The better performance of~~ public sector hospitals in Gujarat performed better and~~ worst performance of~~ private sector hospitals in Madhya Pradesh performed poorly at~~in~~ this first contact point for patient interaction. These performance levels mirrored~~ corroborated well with the pattern of~~ patient satisfaction regarding beneficiary identification and registration. Ensuring access to functional helpdesks at PM-JAY's empaneled hospitals and using ICT to enable two-way communication with beneficiaries have emerged as important tools that make the scheme more responsive to its clients and which need to be further expanded. These efforts will also contribute to patient-level insurance education.

The duration of~~time taken in~~ various non-clinical processes was also~~is~~ an important element of service delivery responsiveness. This~~ The importance of the quality of non-clinical care~~ was evident~~reiterated~~ in the~~ form of~~ higher satisfaction among patients who experienced lower waiting times during~~time in~~ package selection, admission, and discharge processes. The connection of low waiting time and higher patient satisfaction was also observed among the beneficiaries of Rashtriya Swasthya Bima Yojana (RSBY). [31] This~~. The~~ finding of the importance to patients of prompt attention and short~~with low~~ waiting times~~time~~ is similar to observations from~~the one observed in~~ the Nigerian social health insurance scheme, which highlighted the three important elements as priority areas for action to improve responsiveness:~~importance of a)~~ autonomy in terms of the involvement of patients in decisions;~~, b)~~ communication between~~of~~ providers and~~with~~ beneficiaries;~~,~~ and~~, c)~~ prompt

attention with low waiting times.~~time as priority areas for action to improve responsiveness.~~ [7]~~[9]~~

The high incidence and extent of OOP payments~~payment~~, especially in Madhya Pradesh, ~~and~~ corresponding with low levels of ~~poor~~ patient satisfaction, is ~~along the lines of~~ similar to other GSHIS experiences in India. [18, 27, 28]~~-~~ Such OOP payments in PM-JAY could be connected with the design of the scheme in terms of process flow, poor package rates, and policy of government-reserved procedures. Hospitals may be requiring such payments from patients for ~~Many hospitals may insist on payment of~~ pre-operative diagnostic procedures due to concern~~fearing~~ that they will not receive~~there is~~ a payment if~~possibility that~~ the patient is~~may~~ not ultimately~~be~~ hospitalized. Patients ~~based on the negative results. The policymakers needs to look into this design feature. Next, the patients~~ in private hospitals may also be asked to make payments for~~in~~ certain multimodal treatments that are ~~include hospitalization with surgical as well as medical treatment, which are exclusively~~ reserved for public facilities. This is most relevant~~especially important~~ for patients with conditions, like cancer, that requires customized ~~where there is a need for customizing~~ treatment by a team of doctors using multiple treatment modalities. Finally,~~Last, the~~ patients may be asked to pay for the difference in costs~~expenses~~ if the hospital chooses a~~choice of~~ treatment that~~(for example, medicine or implant)~~ is different from what is covered as a package reimbursement (for example, medicine or an implant for treatment of a cardiovascular condition).~~.~~ [34]~~[29]~~ Irrespective of the reasons, ~~the~~ OOP payments affect~~affects~~ financial and physical coverage and affect~~ultimately affects~~ user satisfaction. If not addressed optimally, such payments can lead to a paradoxical situation in which ~~of poor financial and physical coverage, despite~~ an overall increase in the utilization of services results in poor financial and physical coverage.~~.~~ [35]

The scheme managers should streamline the existing treatment guidelines following the prevailing standard practices among the healthcare providers, but there will always be gaps between options covered by the scheme and the best options from providers' perspectives. It may be difficult for policymakers to revise existing PM-JAY packages now, in the early stages of implementation, due to the need for cost containment. However, with the expansion of coverage and utilization over time, it will be important to ensure upward revision of the existing treatment package rates to reduce OOP payments and increase patient satisfaction with PM-JAY. The benefit package rates and the restriction of certain procedures to government hospitals need to be further rationalized by streamlining existing treatment guidelines. Also, information about the list of procedures reserved for government hospitals needs to be communicated effectively to patients.~~[30]~~

Lastly, ~~beneficiary feedback - i.e.~~ communication among~~between the~~ patients, ~~and~~ healthcare providers and ~~as well as~~ insurance providers remain~~as well and acting upon the inputs of the stakeholders – remains~~ an important element of the responsiveness of the health insurance system, as does acting upon these inputs from stakeholders.~~–~~ In PM-JAY, an~~this~~ integrated mechanism for~~of~~ information exchange with patients is based on the ~~patients' i.e.~~ timely provision of SMSs and calls for various hospital-based transactions. However, this study found that the mechanism is~~to have been~~ performing sub-optimally, with significant room for~~a scope of~~ improvement in ~~terms of~~ its reach. Survey respondents' reports~~The instances~~ of low~~poor~~ patient satisfaction due to~~because of~~ bad provider behaviour also points to the need for a mechanism to collect such information ~~regularly~~ and tie it to corrective actions. ~~thereof.~~

The study has the following limitations. First, the sample size is small. In future, similar studies with a larger sample size need to be carried out to explore the phenomena from a larger pool of PM-JAY beneficiaries. Second, for this study the hospitals to shortlist PM-JAY beneficiaries

were selected by the respective SHAs. This may have unknowingly introduced some biases. In future, studies may look at alternate ways of sampling the hospitals and patients.

Conclusion

ThisIn this research explored, the experiences of patients who used hospital services during an availed hospitalization in the early phase of PM-JAY implementation. of PM-JAY were examined. The findings provide insight into the scheme's responsivenessresearch provided certain valuable insights about the ability of the scheme in being responsive and success in providing financial protection. These findings highlight It highlighted the importance of prompt attention and timely access to information as important attributes of patients' non-clinical expectations of health care services.patients. To that end, functional helpdesks at the empanelled hospitals and a vibrant ICT- enabled two-way communication with beneficiaries have emerged as important elements to make the scheme more responsive. Such efforts will also contribute to patient-level efforts for insurance education. Access to better information shouldmay enable PM-JAY'sthe beneficiaries to make informed choices and seek accountability from the healthcare providers and the scheme managers, which ultimately wouldcan enhance the performance of the scheme on all three fronts of health outcomes, responsiveness and financial protection. .

Ensuring financial protection through reducingreduced OOP expenses is anotheran additional agenda forof action by PM-JAY. This studyfor the planners of the scheme. The research highlighted that PM-JAY beneficiaries still facethe existing high OOP costs, through in PM-JAY as the forbidden practice of balance billing, resulting from the is a result of intricacies ofin provider payment mechanisms and poor governance. While there is a need to streamline the existing treatment guidelines in accordance with prevailing standard practice, there would always remain a gap in optimal choices provided under the scheme and the best available

Formatted: Font: 12 pt

Formatted: Heading 1, Left, Space Before: 0 pt, After: 0 pt, Line spacing: single

options from the provider's perspective. It may be difficult for the planners to revise the existing package rates in the early stage of implementation due to the cost containment. However, it is important to ensure upward revision in the existing low rates of treatment packages with the expansion of coverage and utilization, to reduce the OOP and increase patient satisfaction in PM-JAY.

Lastly, the findings indicateresearch pointed out that the policy of restricting certain procedures to public hospitals bothnot only reduces financial coverage through OOP costs and , but it also limits physical coverage and choice of provider, which are bothproviders — an important elementselement of health system responsiveness. The package rates and the restriction of packages to government hospitals need to be rationalized by streamlining the existing treatment guidelines. Also, the information on the list of procedures reserved for government hospitals needs to be communicated effectively to the patients so that they are not caught unaware.

This research provides importantTo sum up, this research contributes to the existing literature by providing insights from PM-JAYthe beneficiaries based on their experiencesexperience of receiving treatment at hospitalshospitalization under the new scheme.PM-JAY. The results of this study emphasize that the inter-connected issues of responsiveness and financial protection are inter-connected issues for a in the health insurance system; they also, and highlight several areas where the changes are neededthat need to be made to strengthen the scheme. Because PM-JAY While there is a new scheme, the thin literature on various aspects of it remains limited. Research on the PM-JAY being a new scheme, the research on responsiveness of GSHIS schemes, in general, isare also limited. This studyfew. In this context, this research provides new and valuable evidence in this sphere and offers policymakers options for improvingavenues of improvement in the implementation of PM-JAY. Perfected

implementation of this pioneering scheme will have a major impact on the health of many of India’s poorest citizens and help the country move toward achieving UHC.

Acknowledgements

We are thankful to the National Health Authority, India, for the guidance and necessary approvals for the research. We ~~also~~ acknowledge the inputs of WHO India team members Dr Vinod Verma in conducting the research, and Dr~~.~~ Grace Kabaniha ~~(WHO, India)~~ in improving the manuscript. We would also like to thank Dr Chandrakant Lahariya and Dr Hilde De Graeve for their support and guidance.

Formatted: Font: 12 pt, Font color: Auto

Formatted: Heading 1, Left, Line spacing: single, Don't suppress line numbers

Formatted: Don't suppress line numbers

Formatted: Font color: Auto

References

1. World Health Organization. The world health report: health systems financing: the path to universal coverage. Geneva: World Health Organization; 2010.
2. WHO. The World Health Report 2000: Health Systems : Improving Performance. Geneva: World Health Organization, 2000 9789241561983.
3. Murray CJL, Frenk J, World Health Organization. Global Programme on Evidence for Health P. A WHO framework for health system performance assessment / Christopher J. L. Murray, Julio Frenk. Geneva: World Health Organization; 1999.
4. Olmen Jv, Criel B, Bhojani U, Marchal B, Belle Sv, Chenge MF, et al. The Health System Dynamics Framework: The introduction of an analytical model for health system analysis and its application to two case-studies. 2012. 2012;2(1):21. Epub 2012-05-15. doi: 10.5195/hcs.2012.71.
5. Thi Thu Ha B, Mirzoev T, Morgan R. Patient complaints in healthcare services in Vietnam's health system. SAGE Open Medicine. 2015;3:2050312115610127. doi: 10.1177/2050312115610127. PubMed PMID: 26770804.
6. Paul S. Public Spending, Outcomes and Accountability: Citizen Report Card as a Catalyst for Public Action. Economic and Political Weekly. 2006;41(4):333-40.
7. Mohammed Ahmar U. Microinsurance in India: Insurance literacy and demand. Business and Economic Horizons (BEH). 2017;13(2):182-91 DOI: 10.15208/beh.2017.14. doi: DOI: 10.15208/beh.2017.14.
8. Cole S, Giné X, Tobacman J, Topalova P, Townsend R, Vickery J. Barriers to Household Risk Management: Evidence from India. American Economic Journal: Applied Economics. 2013;5(1):104-35. doi: 10.1257/app.5.1.104.
9. Mohammed S, Bermejo JL, Souares A, Sauerborn R, Dong H. Assessing responsiveness of health care services within a health insurance scheme in Nigeria: users' perspectives. BMC Health Services Research. 2013;13(1):502. doi: 10.1186/1472-6963-13-502.
10. Valentine NB, de Silva A, Kawabata K. Health system responsiveness: concepts, domains and operationalization. In: Darby C, Murray CJ, Evans DB, editors. Health systems performance assessment: debates, methods and empiricism. 96. Geneva: World Health Organization; 2003. p. 573–96.
11. Carrin G, James C. Reaching universal coverage via social health insurance: key design features in the transition period2004; 13(EIP/FER/DP.04.2). Available from: https://apps.who.int/iris/handle/10665/69018.
12. WHO. Everybody's business -- strengthening health systems to improve health outcomes : WHO's framework for action. Geneva: World Health Organization; 2007.
13. United Nations. Transforming our world: the 2030 Agenda for Sustainable Development. New York, USA,: United Nations Publications; 2015.
14. Giedion U, Andrés Alfonso E, Díaz Y. The impact of universal coverage schemes in the developing world: a review of the existing evidence. Wahington DC: The World Bank; 2013.
15. Escobar ML, Griffin CC, Shaw RP. The Impact of Health Insurance in Low- and Middle-Income Countries. Wasahington DC: Brookings Institution Press; 2011.
16. Garg S, Chowdhury S, Sundararaman T. Utilisation and financial protection for hospital care under publicly funded health insurance in three states in Southern India. BMC Health Services Research. 2019;19. doi: https://doi.org/10.1186/s12913-019-4849-8.
17. Prinja S, Chauhan AS, Karan A, Kaur G, Kumar R. Impact of Publicly Financed Health Insurance Schemes on Healthcare Utilization and Financial Risk Protection in India: A Systematic Review. PLOS ONE. 2017;12(2):e0170996. doi: 10.1371/journal.pone.0170996.
18. Devadasan N, Seshadri T, Trivedi M, Criel B. Promoting universal financial protection: evidence from the Rashtriya Swasthya Bima Yojana (RSBY) in Gujarat, India. Health Research Policy and Systems. 2013;11(1):29. doi: 10.1186/1478-4505-11-29.

Formatted: Font: 12 pt

Formatted: Heading 1, Left, Space Before: 0 pt, Line spacing: single

Formatted: Font: 12 pt

19. Karan A, Yip W, Mahal A. Extending health insurance to the poor in India: An impact evaluation of Rashtriya Swasthya Bima Yojana on out of pocket spending for healthcare. Social Science & Medicine. 2017;181:83-92. doi: https://doi.org/10.1016/j.socscimed.2017.03.053.
20. Mirzoev T, Kane S. What is health systems responsiveness? Review of existing knowledge and proposed conceptual framework. BMJ Global Health. 2017;2(4):e000486 doi: 10.1136/bmjgh-2017-. doi: 10.1136/bmjgh-2017-000486.
21. La Forgia G, Nagpal S. Government-Sponsored Health Insurance in India: Are You Covered? New Delhi: World Bank; 2012.
22. Peters DH, Kanjilal B. Health insurance & responsiveness to communities & patients: the future of health systems in India. Indian J Med Res. 2011;133(1):9-10. PubMed PMID: 21321414.
23. NHA. Guidelines for Pradhan Mantri Rashtriya Swasthya Suraksha Mission (PMRSSM). 2018.
24. PIB. Cost of Treatment Under Ayushman Bharat PMJAY. In: GoI MoSHaFW, editor. Delhi: Press Information Bureau, GoI; 2019.
25. NHA. Lessons Learned in one year implementation of PM-JAY. New Delhi: National Health Authority, 2019.
26. NHA. PMRSSM GUIDELINES ON PROCESSES FOR HOSPITAL TRANSACTION. Delhi2018.
27. Sundararaman T, Muraleedharan V, Mukhopadhyay I. NSSO 71st round data on health and beyond: Questioning frameworks of analysis. 2016;51:85-8.
28. Nandi S, Schneider H, Dixit P. Hospital utilization and out of pocket expenditure in public and private sectors under the universal government health insurance scheme in Chhattisgarh State, India: Lessons for universal health coverage. PLOS ONE. 2017;12(11):e0187904. doi: 10.1371/journal.pone.0187904.
29. Trivedi M, Saxena A, Mavalankar D, Sharma M. Improving Hospital-Based Processes for Effective Implementation Of PMJAY: Evidence from Early Implementation. New Delhi: National Health Authority, 2019.
30. Gupta I, Chowdhury S, Trivedi M, Prinja S. Do health coverage schemes ensure financial protection from hospitalization expenses? Evidence from eight districts in India. Journal of Social and Economic Development. 2017;19(1):83-93. doi: 10.1007/s40847-017-0040-4.

Editor Comments:

Comment: Studying responsiveness, quality and financial protection for the newly launched PMJAY scheme will add value to Indian policy and is useful for international literature to know factors that influence these attributes for government insurance schemes. However, the paper needs to be better structured to follow it more clearly, especially for international audience. There are several Grammatical issues and certain sections are really lengthy. The discussion and conclusion section needs to be better linked to results.

Response: We have revised the manuscript substantially with the help of a professional editor. This has reduced grammatical errors and improved the content.

Comment: The indicators under responsiveness, quality and financial protection must be explained upfront.

Response: These are now defined and discussed in the methodology section. Please refer to lines number 196-211.

Comment: The abstract needs to be rewritten with clear results and conclusions. Abstract needs revision. Does not present any results from the text and conclusion does not follow from the result.

Response: We have revised the abstract to reflect these details.

Comment: The title does not have quality, but you do mention about quality in results. If it is part of responsiveness, please say it clearly. Also AB in the title is not explained or used throughout the manuscript.

Response: We measure quality only in terms of patient satisfaction. As this is a relatively small focus of our research, we chose not to mention this under the title. We have removed AB from the title.

Comment: Very long introduction, needs to be structured with background linked to objectives studied.

Response: We have revised the introduction substantially to improve the linkages of the background with the study objectives. The introduction is reduced by more than fifteen lines.

Comment: In the methods section, clearly explain the variables/ indicators used for responsiveness, quality and financial protection used in your study.

Response: These are now defined and discussed in the methodology section. Please refer to line numbers 196-211.

Comment: The indicators are mixed up under the procedures - from admission to discharge to follow up. EG. Section c has a mix of responsiveness and financial protection in results. Use the same format then for results and discussion. These are given but the presentation needs to be clearer for the reader to follow.

Response: The results of this study were documented using the flow of processes in the standard official guideline as a template. The findings are therefore divided into subsections that correspond to the PM-JAY guidelines for hospital-based transactions. These sub-sections are routine processes carried out for beneficiary identification and registration, treatment package selection and blocking Discharge and payments, and information exchange. The discussion is now streamlined and focused on the objectives of the study along the lines of responsiveness and financial protection.

Comment: Discussion needs to be arranged in the context of major sections studied, what your research says and how does it corroborate with the research from other government health sponsored schemes from past in INDIA such as RSBY or state insurance schemes or other countries. In what ways PMJAY is better than existing insurance schemes and what needs to be improved.

Response: We have revised the discussion section. We have added information on comparisons of findings of this research with similar experiences of previous government health-sponsored schemes, including RSBY. We have compared our findings of awareness of schemes (Please refer to line number 379-381), and about waiting- time and satisfaction (Please refer to line number 397-398). Additionally, a comparison around OOP has already existed in the manuscript. Please refer to line number 404-406.

1. Please ensure that your manuscript meets PLOS ONE's style requirements, including those for file naming. The PLOS ONE style templates can be found at

https://journals.plos.org/plosone/s/file?id=wjVg/PLOSOne_formatting_sample_main_body.pdf
and

https://journals.plos.org/plosone/s/file?id=ba62/PLOSOne_formatting_sample_title_authors_affiliations.pdf

Response: We have revised the manuscript following PLOS ONE's style requirements.

2. Please include additional information regarding the survey or questionnaire used in the study and ensure that you have provided sufficient details that others could replicate the analyses. For instance, if you developed a questionnaire as part of this study and it is not under a copyright more restrictive than CC-BY, please include a copy, in both the original language and English, as Supporting Information.

Response: We have submitted a copy of the questionnaire as supplementary material.

3. Please provide additional details regarding participant consent. In the ethics statement in the Methods and online submission information, please ensure that you have specified how verbal consent was documented and witnessed.

Response: A participant information sheet was provided to the respondents. They were provided all details of the research. Their participation was requested through verbal, non-witnessed informed consent. We have provided these details in the manuscript. Please refer to line number 188-192.

4. We note that you have indicated that data from this study are available upon request. PLOS only allows data to be available upon request if there are legal or ethical restrictions on sharing data publicly. For information on unacceptable data access restrictions, please see http://journals.plos.org/plosone/s/data-availability#loc-unacceptable-data-access-restrictions.

In your revised cover letter, please address the following prompts:

a) If there are ethical or legal restrictions on sharing a de-identified data set, please explain them in detail (e.g., data contain potentially identifying or sensitive patient information) and who has imposed them (e.g., an ethics committee). Please also provide contact information for a data access committee, ethics committee, or other institutional body to which data requests may be sent.

b) If there are no restrictions, please upload the minimal anonymized data set necessary to replicate your study findings as either Supporting Information files or to a stable, public repository and provide us with the relevant URLs, DOIs, or accession numbers. Please see http://www.bmj.com/content/340/bmj.c181.long for guidelines on how to de-identify and prepare clinical data for publication. For a list of acceptable repositories, please see http://journals.plos.org/plosone/s/data-availability#loc-recommended-repositories.

We will update your Data Availability statement on your behalf to reflect the information you provide.

Response: This data contained in this study were made available to the research team by the National Health Authority, India for exclusive use in this study. The data belong to the National Health Authority. For more information please contact Dr Ruchira Agrawal , Consultant M&R, National Health Authority at Mel.cons1@nha.gov.in ." We have revised these details in the cover letter.

5. Your ethics statement should only appear in the Methods section of your manuscript. If your ethics statement is written in any section besides the Methods, please delete it from any other section.

Response: This information is presented only in the method section.

6. We noted in your submission details that a portion of your manuscript may have been presented or published elsewhere. [The contents of this manuscript have not been submitted for publication elsewhere. A modified version of this manuscript is available as a working paper on https://pmjay.gov.in/sites/default/files/2020-02/WP_IIPH_study_2.pdf.] Please clarify whether this publication was peer-reviewed and formally published. If this work was previously peer-reviewed and published, in the cover letter please provide the reason that this work does not constitute dual publication and should be included in the current manuscript.

Response: This is not a peer-reviewed publication. This is also not formally published and is only put on the website of the national health authority as one of many outputs of the research carried out on PMJAY. This is a mandatory requirement of the authority. This working paper contains wider information beyond the objective of the manuscript submitted for publication. We have modified the cover letter to highlight these details.

Reviewers' #1 comments:

General comments: The manuscript reviews major language editing and rewriting. There are far too many language errors across the manuscript, which makes it non readable. All sections need major editing and shortening. The repetition should be eliminated. The discussion section needs to be improved with editing and in the quality of analysis.

Response: We have revised the manuscript substantially with the help of a professional editor. This has reduced grammatical errors and improved the content.

Specific comment 1: Abstract need to be completely re-written. It does not capture the essence of manuscript. There are 200 study participants from whom data was collected. Some quantitative analysis findings should be part of the abstract, which currently focuses upon qualitative aspects only. The conclusion in abstract is generic. Please re-write. The acronyms can be avoided.

Response: We have revised the abstract to reflect these details.

Specific comment 2: Financial disclosure: Please confirm if this is full disclosure of grant or was there any other funding in addition to Health policy and system research at WHO HQ? Please also confirm if authors have acknowledged all the key people who contributed to this work?

Response: We have revised the financial disclosure statement. It now reads, "The research was made possible through the financial support provided by the World Health Organization. This included support from the Alliance for Health Policy and Systems Research, WHO Geneva as well as from the WHO Country Office, India under grant numbers 68345 (WHO, Geneva) and 67378 (WHO, India). Support was provided under the research programme titled- Health Systems Research for PM-JAY: Improving hospital-based processes for effective implementation. The grant was received by Prof. Dileep Mavalankar, Prof. Mayur Trivedi, and Prof. Anurag Saxena of the Indian Institute of Public Health Gandhinagar. The grant period was from 4 March to 3 September 2019. The URL of the funder is www.who.int. Dr Zubin Shroff of WHO provided guidance in strengthening the methodology and finalizing the instruments, and reviewed the manuscript critically."

We have revised the acknowledgment statement. It now reads, "We are thankful to the National Health Authority, India, for the guidance and necessary approvals for the research. We acknowledge the inputs of WHO India team members Dr. Vinod Verma in conducting the research, and Dr. Grace Kabaniha in improving the manuscript. We would also like to thank Dr. Chandrakant Lahariya and Dr. Hilde De Graeve for their support and guidance. Please refer to line number 472-476.

Specific comment 3: Ethical statement: was there any specific reason that only verbal consent was sought and not the written consent?

Response: The respondents as poor beneficiaries of the PMJAY scheme were expected to have a low level of education to be poor and would be illiterate or semi-literate individuals from urban and rural areas. Since the data collection for the survey was done using handheld digital devices, the thumb impression was difficult to record. Therefore, informal verbal consent was obtained. However, the respondents were provided all details of the research, including their rights to refuse to participate and not answer any questions. We have provided these details in the manuscript. Please refer to line number 188-192.

Specific comment 4: Introduction / main text: The language need major re-writing for academic standards. The wordings such as; line 3: 'people may remain unhappy with the health systems" could be avoided. This section is a bit superfluous and verbose. It need to edited and shortened. There are total 108 lines, it is difficult to follow and read. The information about PM-AY can be moved into a box.

Response: We have revised the introduction substantially. The introduction is also shortened by more than fifteen lines. The line "people may remain unhappy with the health systems" is also removed. The information on PMJAY Is now reduced and thus, not moved in a box.

Specific comment 5: Methods section, there is duplication of information and repetitions.

Response: We have revised the methodology section substantially to remove duplication of information and repetitions.

Specific comment 6: Results section. One of the limitations is that the small sample size and then then findings have been used in both tables and text.

Response: We have revised the results section to remove repetitions in the findings that have been used in both tables and text. We have acknowledged the small sample size as one of the limitations. Please refer to line number 438-443.

Specific comment 7: In discussion, the key actionable suggestions should be included. The limitations of this work need to be elaborated. More India specific literature review and citations should be used.

Response: We have revised the discussion section substantially. Certain key actionable suggestions were provided under the conclusion section. These are now shifted to the discussion section. Also, we have added India-specific literature while comparing our findings of awareness of schemes. Please refer to line number 379-381. We have also added literature on waiting time and satisfaction. Please refer to line number 397-398. Additionally, a comparison around OOP has already existed in the manuscript. Please refer to line number 404-406. The limitations of this work are added in the discussion section. Please refer to line number 438-443.

Specific comment 8: Conclusion: Should be succinct and single or maximum of two paragraphs.

Response: We have revised the conclusion section substantially. After shifting certain key actionable suggestions to the discussion section, the conclusion section is now of two paragraphs only.

Specific comment 9: Figure 1- 4 are repetition and can be removed.

Response: Agreeing to the suggestion, we have removed these figures, as the details are provided in the text. These are now provided as supplementary material.

Reviewer #2 comments:

Specific comment 1. The paper may give some context of health care utilization and expenditure about the states chosen

Response: We have now added this information, along with a Table. Please refer to line number 148-49 and Table 1 for these details.

Specific comment 2.Need to strengthen the discussion section with some comparison of the findings of PM-JAY with private health insurance schemes to judge the strengths and shortcomings of PM-JAY.

Response: We couldn't find relevant research on responsiveness and financial protection from the perspective of beneficiaries of private health insurance schemes. Thus, we could not add such a comparison of PMJAY with private health insurance schemes. However, we have compared our findings with other GSHIS. Please refer to line number 406-420 for these details.

Specific comment 3. Quantitative analysis may be augmented by estimating odds-ratios for the chosen indicators.

Response: The study did not analyze the associations or determinants of responsiveness (measured in terms of patient satisfaction) and financial protection (measured in terms of incidence and magnitude of OOP). Estimation of odds ratio was therefore not attempted as it was not related to the study objectives. Additionally, small sample size was a limitation of the study restricting the scope of statistical analysis. We, however, explored the subgroup difference in responsiveness and financial protection across socio-economic categories of the respondents. These details are presented in Table 1 as supplementary material.

Specific comment 4. The word "improving" may be dropped from the title.

Response: Agreeing to the suggestion, we have removed the word 'improving' from the title.

Specific comment 5. There are many grammatical errors and many sentences need editing to have a more terse presentation.

Response: We have revised the manuscript substantially with the help of a professional editor. This has reduced grammatical errors and improved the content.

Specific comment 6. In line 251, the figures in the Table and in the description do not match.

Response: These were because the findings were presented to highlight the proportion of the respondents who did not make any OOP payments. We have now revised the language and figures to reflect the information in terms of those who made the OOP payment, as presented in the table

Specific comment 7. Line 274, and other lines, the style of writing figures is not uniform.

Response: We have corrected the style of writing figures to make them uniform at all places.

Specific comments in the attached document:

Comment 1: check grammar.

Response: We have revised the abstract and edited it for grammatical errors.

Comment 2: The abbreviation in title and text should be the same, otherwise need to repeat the full form here as well.

Response: We have revised the abbreviation in the title.

Comment 3: Indicate dollar values also

Response: We have revised the abstract and added dollar values.

Comment 4: delete. Repeated

Response: We have revised the abstract accordingly.

Comment 5: Avoid abbreviation

Response: We have revised the abstract accordingly.

Comment 6: The conclusion seems more like objective and does not follow from results.

Response: We have revised the conclusion part of the abstract accordingly.

Comment 7: Very long introduction. Clearly provide the background in the context of the objective of the study.

Response: We have revised the introduction substantially. The introduction is also shortened by more than fifteen lines.

Comment 8: Please place references as per the plos one style - different places before and after full stops.

Response: We have revised the references as per PLOS style. They are now after full stops at all places.

Comment 9: check grammar.

Response: We have revised the entire document for grammatical errors.

Comment 10: Major features to be more succinct and summarized in a box under headings

Response: We have revised the introduction of PM-JAY substantially and now it is shorter, and in the flow of the text. At this stage, it need not be in the box. However, if the reviewer feels appropriate, we would be happy to put the details in the box.

Comment 11: Please provide full references with links and dates accessed as per PLOS style.

Response: We have revised this reference. We have used the 'PLOS' style in the EndNote referencing software for generating full references for this manuscript.

Comment 12: what are autonomous hospitals

Response: These are publicly funded hospitals that have special status through legislation and thus, are governed independently. However, since this was not relevant for the objectives, we have removed this sentence.

Comment 13: what if the claim os from the same episode of ailment.

Response: All patients with single claims were retained in the population list. Few patients required multiple sessions of treatment like dialysis and chemotherapy. In such cases, each session was claimed separately, and thus, there were multiple claims of those patients. We retained all these patients.

Comment 14: Would this not add a bias for responsiveness, as the hospitals with least claims can also be the ones where people did not want to go.

Response: The hospitals for the study were selected by the state authorities. It was important to ensure the representation of patients across hospitals and specialties within the hospitals. The use of multi-stage sampling method with the use of Probability Proportional to Size ensured that the selection of respondents was not biased either in favour or against any type of specialty and hospital. This information is now explained in the manuscript. Please refer to line number 169-171.

Comment 14: They would already have better knowledge of the health scheme, so their responsiveness may be better.

Response: The selection of the state with previous experience of implementing a GSHIS was an element of study at the time of selection of the study location. The responsiveness in terms of their registration in the scheme was not analyzed and therefore, 'n' for the beneficiary registration did not include these patients. The responsiveness of their hospitalization experience was measured only for the particular episode during the study duration. Thus, such possibility of their experience influencing the knowledge and responsiveness of the scheme may be ruled out.

Comment 15: Can be added as supplementary material

Response: We have submitted a copy of the questionnaire as supplementary material.

Comment 15: Would this not create seasonal bias

Response: The study population includes patients who availed hospitalization under PMJAY during April 2019. This was given by the state authorities, and thus, was not under the control of the researchers. The data collection was done during May-June 2019. There could be seasonal effects on the type of illnesses, but the responsiveness and financial protection may not get affected by the time of the year.

Comment 16: Can say informed consent was taken

Response: We have revised the statement and provided more details in the manuscript. Please refer to line number 190-194.

Comment 17: These can be summarized under different headings in a box - in the same format as the results are presented. Some of it also repeated from above. Need to add them together.

Response: These are now well-defined and discussed in the methodology section between lines 196-211. We have removed the repetition. The results of this study were documented using the flow of processes in the standard official guideline as a template. The findings are therefore divided into subsections that correspond to the PM-JAY guidelines for hospital-based transactions. These sub-sections are beneficiary identification and registration, treatment package selection and blocking, discharge and payments, and information exchange.

Comment 18: Is anything covered before and after hospitalization. Was information collected on experience before and after hospitalization? If referral etc. had the influence on registration process. What about coverage after hospitalization.

Response: The study collected information on pre-and post-hospitalization expenses in detail. The analysis of OOP payment includes these details in light of the provision of such coverage under the scheme. The information exchange is an in-built element regarding a) registration done before or at the time of the hospitalization, b) package selection during hospitalization, and c) calls for feedback after the hospitalization. These were studied in detail.

Comment 19: why is education of beneficiary/ or highest education in HH not taken as indicator, as awareness and responsiveness depends quite a bit on that.

Response: We have now added this information in Table 3.

Comment 20: mismatch - 61 vs 74. Also if they were already enrolled in a scheme, one would expect better experience about the process. Could it be they did not want to switch.

Response: This is not a mismatch. 74% of the total 61 respondents (i.e. 45 respondents) indicated that they knew about the scheme from the letter. The remaining 39 participants at the time of the research were yet to be transferred from the MA scheme to PMJAY. For all practical purposes, all beneficiaries, irrespective of the scheme received the same coverage for their hospitalization. We did not include these 39 participants for their registration experience as they were not registered in

PMJAY till April 2019. The participants did not have choice in switching as the existing scheme was getting subsumed under PMJAY over time.

Comment 21: what was the difference in public vs. private will be useful to know

Response: The satisfaction was higher among beneficiaries from public sector hospitals (83%) than their counterparts from the private sector (77%). This is now added in the result section. Please refer to line number 296 and 297.

Comment 22: You mention preauthorization above also. These are longer than for admission and preauthorization. Do you mean only admission above? Otherwise hours should be more above.

Response: The first mention of pre-authorization is in the context of 'admission and pre-authorization request' – i.e. time that the AM took in preparing and submitting the request. The second mention is about 'pre-authorization approval' to be received from the scheme authorities. The first one is the efficiency of the insurance processes at the level of healthcare providers, and the latter is about the efficiency of the scheme managers at the level of the insurance company / Trust. These details are clarified and streamlined in the subsection of 'treatment package selection and blocking' in the result section.

Comment 23: Why is the sum of total smaller than public plus private

Response: The total of 'n' is 100 (after adding 56 private and 44 public). The incidence of OOP is in proportion. So, the total need not be the summation of the two proportions. It is the mean of the two (i.e. 13 and 9).

Comment 24: Does PMJAY not cover the medicine bills for medicines not available. Also for a procedure, would everything not be covered?

Response: Ideally, all expenses should be covered. According to the guideline, the patient need not pay and the hospital must not do balance billing. However, the finding of this study, therefore, brings evidence on OOP and connects it to the reasons for such OOP in the discussion section. We, therefore, raise the need to address underlying supply-side reasons for OOP.

Comment 25: Are they informed and consulted about it before the registration.

Response: Yes. In most cases, the patients were informed beforehand about the possibility of such part payments. This was ascertained during the survey through informal discussions with the respondents.

Comment 26: Is insurance agency or the scheme informed about these payments charged to the patients?

Response: The survey respondents did not know of such communication. Therefore, this is not part of our analysis. However, since part payment is not allowed under the scheme, there is no mechanism/ mandate for the hospitals to make this information available to the insurer/scheme managers.

Comment 27: This is really high. Can this be compared with OOPS data from HH survey.

Response: This is high in the case of patients who availed of hospitalization in a private hospital in Madhya Pradesh, a state which saw such GSHIS with 5 lac coverage for the first time. Therefore, this needs to be seen contextually for the GSHISs covering secondary and tertiary hospitalization. We refrained from comparing the magnitude of OOP of this study (which was measured for those who made some payment) with similar data from the HH survey as the study population is not comparable. Our study population comprises those who exclusively opted for secondary and tertiary hospitalization, and thus are not comparable with households incurring expenses on a range of healthcare, including primary care and outpatient treatment. We have compared the incidence of OOP among the insured patients with similar research on beneficiaries of past GSHIS like RSBY that offered relatively low coverage. Please refer to line number 406-420 for these details.

Comment 28: This should and most of it is part of introduction. Not required here.

Response: We have revised this paragraph and removed irrelevant sentences from here.

Comment 29: What about the links to the socio economic conditions of the households. Also does education matter.

Response: The study did not analyze the determinants of responsiveness (measured in terms of patient satisfaction) and financial protection (measured in terms of incidence and magnitude of OOP). However, on comparing these parameters across the socio-economic subgroups of the respondents, it was found that the satisfaction for registration (91%) and hospitalization (82%) was highest among the illiterate respondents. This sub-group has the lowest incidence of OOP (15%), but reported the highest amount of OOP payment (Rs. 41075). We have provided this information with a table as supplementary material.

Comment 30: What about the links to the rights and information. Also not discussed clearly about the payments and links to Aarogya Mitra. Should AM not play a role in helping the patients to keep their payments low. Even if AM desk is not there in hospitals, should AM not visit the hospitalized patient under the scheme. That would improve the quality and financial protection.

Response: Aarogya Mitra is a staff of the hospital appointed to streamline the liaison between patients, doctors, and scheme authorities. As an employee of the hospital, the AM interacts with the patients on behalf of the hospitals – at the helpdesk or bedside. The AM’s interaction with the patient is limited to information and documentation for preauthorization and discharge. As for the payment, the AM merely conveys the decision of ‘balance payment’, if any, taken by the

hospital authorities. Lastly, being a household survey of the patients, this research did not dwell deeper in the supply side issues of how AM can play an improved role at the provider level.

Comment 31: how do this and some other conclusions follow from your results. Please restrict discussion and conclusions to what your results show.

Response: This is connected to the reasons that patients indicated for their OOP, especially in the case of those who made a lump sum payment. These patients reported that they were informed at the helpdesk that the actual costs of their treatments or procedures were higher than the amount the hospital would receive from claim reimbursements from PM-JAY; thus, patients needed to pay the balance. This is indicated in the result section (Please refer to line number 32-335). We have revised the language and added corresponding arguments and evidence (Please refer to line number 414-416).
