# Supplementary material for: Experiences and challenges in accessing hospitalization in a government-funded health insurance scheme: Evidence from early implementation of Pradhan Mantri Jan Aarogya Yojana (PM-JAY) in India
Source: PLoS One. 2022 May 12;17(5):e0266798. doi: 10.1371/journal.pone.0266798 (PMC9098065; doi:10.1371/journal.pone.0266798)
Supplement: Attachment — Submitted filename: Response to Reviewers.docx [file pone.0266798.s004.docx]

### Editor Comments:

**Comment:** Studying responsiveness, quality and financial protection for the newly launched PMJAY scheme will add value to Indian policy and is useful for international literature to know factors that influence these attributes for government insurance schemes. However, the paper needs to be better structured to follow it more clearly, especially for international audience. There are several Grammatical issues and certain sections are really lengthy. The discussion and conclusion section needs to be better linked to results.

**Response:** We have revised the manuscript substantially with the help of a professional editor. This has reduced grammatical errors and improved the content.

**Comment:** The indicators under responsiveness, quality and financial protection must be explained upfront.

**Response:** These are now defined and discussed in the methodology section. Please refer to lines number 196-211.

**Comment:** The abstract needs to be rewritten with clear results and conclusions. Abstract needs revision. Does not present any results from the text and conclusion does not follow from the result.

**Response:** We have revised the abstract to reflect these details.

**Comment:** The title does not have quality, but you do mention about quality in results. If it is part of responsiveness, please say it clearly. Also AB in the title is not explained or used throughout the manuscript.

**Response:** We measure quality only in terms of patient satisfaction. As this is a relatively small focus of our research, we chose not to mention this under the title. We have removed AB from the title.

**Comment:** Very long introduction, needs to be structured with background linked to objectives studied.

**Response:** We have revised the introduction substantially to improve the linkages of the background with the study objectives. The introduction is reduced by more than fifteen lines.

**Comment:** In the methods section, clearly explain the variables/ indicators used for responsiveness, quality and financial protection used in your study.

**Response:** These are now defined and discussed in the methodology section. Please refer to line numbers 196-211.

**Comment:** The indicators are mixed up under the procedures - from admission to discharge to follow up. EG. Section c has a mix of responsiveness and financial protection in results. Use the same format then for results and discussion. These are given but the presentation needs to be clearer for the reader to follow.

**Response:** The results of this study were documented using the flow of processes in the standard official guideline as a template. The findings are therefore divided into subsections that correspond to the PM-JAY guidelines for hospital-based transactions. These sub-sections are routine processes carried out for beneficiary identification and registration, treatment package selection and blocking Discharge and payments, and information exchange. The discussion is now streamlined and focused on the objectives of the study along the lines of responsiveness and financial protection.

**Comment:** Discussion needs to be arranged in the context of major sections studied, what your research says and how does it corroborate with the research from other government health sponsored schemes from past in INDIA such as RSBY or state insurance schemes or other countries. In what ways PMJAY is better than existing insurance schemes and what needs to be improved.

**Response:** We have revised the discussion section. We have added information on comparisons of findings of this research with similar experiences of previous government health-sponsored schemes, including RSBY. We have compared our findings of awareness of schemes (Please refer to line number 379-381), and about waiting- time and satisfaction (Please refer to line number 397-398). Additionally, a comparison around OOP has already existed in the manuscript. Please refer to line number 404-406.

1. Please ensure that your manuscript meets PLOS ONE's style requirements, including those for file naming. The PLOS ONE style templates can be found at

<https://journals.plos.org/plosone/s/file?id=wjVg/PLOSOne_formatting_sample_main_body.pdf> and

<https://journals.plos.org/plosone/s/file?id=ba62/PLOSOne_formatting_sample_title_authors_affiliations.pdf>

**Response:** We have revised the manuscript following PLOS ONE’s style requirements.

2. Please include additional information regarding the survey or questionnaire used in the study and ensure that you have provided sufficient details that others could replicate the analyses. For instance, if you developed a questionnaire as part of this study and it is not under a copyright more restrictive than CC-BY, please include a copy, in both the original language and English, as Supporting Information.

**Response:** We have submitted a copy of the questionnaire as supplementary material.

3. Please provide additional details regarding participant consent. In the ethics statement in the Methods and online submission information, please ensure that you have specified how verbal consent was documented and witnessed.

**Response:** A participant information sheet was provided to the respondents. They were provided all details of the research. Their participation was requested through verbal, non-witnessed informed consent. We have provided these details in the manuscript. Please refer to line number 188-192.

 4. We note that you have indicated that data from this study are available upon request. PLOS only allows data to be available upon request if there are legal or ethical restrictions on sharing data publicly. For information on unacceptable data access restrictions, please see <http://journals.plos.org/plosone/s/data-availability#loc-unacceptable-data-access-restrictions>.

 In your revised cover letter, please address the following prompts:

 a) If there are ethical or legal restrictions on sharing a de-identified data set, please explain them in detail (e.g., data contain potentially identifying or sensitive patient information) and who has imposed them (e.g., an ethics committee). Please also provide contact information for a data access committee, ethics committee, or other institutional body to which data requests may be sent.

 b) If there are no restrictions, please upload the minimal anonymized data set necessary to replicate your study findings as either Supporting Information files or to a stable, public repository and provide us with the relevant URLs, DOIs, or accession numbers. Please see <http://www.bmj.com/content/340/bmj.c181.long> for guidelines on how to de-identify and prepare clinical data for publication. For a list of acceptable repositories, please see <http://journals.plos.org/plosone/s/data-availability#loc-recommended-repositories>.

 We will update your Data Availability statement on your behalf to reflect the information you provide.

**Response:** This data contained in this study were made available to the research team by the National Health Authority, India for exclusive use in this study. The data belong to the National Health Authority. For more information please contact Dr Ruchira Agrawal , Consultant M&R, National Health Authority at [Mel.cons1@nha.gov.in](mailto:Mel.cons1@nha.gov.in) ." We have revised these details in the cover letter.

5. Your ethics statement should only appear in the Methods section of your manuscript. If your ethics statement is written in any section besides the Methods, please delete it from any other section.

**Response:** This information is presented only in the method section.

6. We noted in your submission details that a portion of your manuscript may have been presented or published elsewhere. [The contents of this manuscript have not been submitted for publication elsewhere. A modified version of this manuscript is available as a working paper on <https://pmjay.gov.in/sites/default/files/2020-02/WP_IIPH_study_2.pdf>.] Please clarify whether this publication was peer-reviewed and formally published. If this work was previously peer-reviewed and published, in the cover letter please provide the reason that this work does not constitute dual publication and should be included in the current manuscript.

**Response:** This is not a peer-reviewed publication. This is also not formally published and is only put on the website of the national health authority as one of many outputs of the research carried out on PMJAY. This is a mandatory requirement of the authority. This working paper contains wider information beyond the objective of the manuscript submitted for publication. We have modified the cover letter to highlight these details.

### Reviewers' #1 comments:

General comments: The manuscript reviews major language editing and rewriting. There are far too many language errors across the manuscript, which makes it non readable.
All sections need major editing and shortening. The repetition should be eliminated. The discussion section needs to be improved with editing and in the quality of analysis.

**Response:** We have revised the manuscript substantially with the help of a professional editor. This has reduced grammatical errors and improved the content.

**Specific comment 1**: Abstract need to be completely re-written. It does not capture the essence of manuscript. There are 200 study participants from whom data was collected. Some quantitative analysis findings should be part of the abstract, which currently focuses upon qualitative aspects only. The conclusion in abstract is generic. Please re-write. The acronyms can be avoided.

**Response:** We have revised the abstract to reflect these details.

**Specific comment 2:** Financial disclosure: Please confirm if this is full disclosure of grant or was there any other funding in addition to Health policy and system research at WHO HQ? Please also confirm if authors have acknowledged all the key people who contributed to this work?

**Response:** We have revised the financial disclosure statement. It now reads, “The research was made possible through the financial support provided by the World Health Organization. This included support from the Alliance for Health Policy and Systems Research, WHO Geneva as well as from the WHO Country Office, India under grant numbers 68345 (WHO, Geneva) and 67378 (WHO, India). Support was provided under the research programme titled- Health Systems Research for PM-JAY: Improving hospital-based processes for effective implementation. The grant was received by Prof. Dileep Mavalankar, Prof. Mayur Trivedi, and Prof. Anurag Saxena of the Indian Institute of Public Health Gandhinagar. The grant period was from 4 March to 3 September 2019. The URL of the funder is [www.who.int](http://www.who.int). Dr Zubin Shroff of WHO provided guidance in strengthening the methodology and finalizing the instruments, and reviewed the manuscript critically.”

We have revised the acknowledgment statement. It now reads, “We are thankful to the National Health Authority, India, for the guidance and necessary approvals for the research. We acknowledge the inputs of WHO India team members Dr. Vinod Verma in conducting the research, and Dr. Grace Kabaniha in improving the manuscript. We would also like to thank Dr. Chandrakant Lahariya and Dr. Hilde De Graeve for their support and guidance. Please refer to line number 472-476.

**Specific comment 3:** Ethical statement: was there any specific reason that only verbal consent was sought and not the written consent?

**Response:** The respondents as poor beneficiaries of the PMJAY scheme were expected to have a low level of education to be poor and would be illiterate or semi-literate individuals from urban and rural areas. Since the data collection for the survey was done using handheld digital devices, the thumb impression was difficult to record. Therefore, informal verbal consent was obtained. However, the respondents were provided all details of the research, including their rights to refuse to participate and not answer any questions. We have provided these details in the manuscript. Please refer to line number 188-192.

**Specific comment 4:** Introduction / main text: The language need major re-writing for academic standards. The wordings such as; line 3: ‘people may remain unhappy with the health systems” could be avoided. This section is a bit superfluous and verbose. It need to edited and shortened. There are total 108 lines, it is difficult to follow and read. The information about PM-AY can be moved into a box.

**Response:** We have revised the introduction substantially. The introduction is also shortened by more than fifteen lines. The line “people may remain unhappy with the health systems” is also removed. The information on PMJAY Is now reduced and thus, not moved in a box.

**Specific comment 5:** Methods section, there is duplication of information and repetitions.

**Response:** We have revised the methodology section substantially to remove duplication of information and repetitions.

**Specific comment 6:** Results section. One of the limitations is that the small sample size and then then findings have been used in both tables and text.

**Response:** We have revised the results section to remove repetitions in the findings that have been used in both tables and text. We have acknowledged the small sample size as one of the limitations. Please refer to line number 438-443.

**Specific comment 7:** In discussion, the key actionable suggestions should be included. The limitations of this work need to be elaborated. More India specific literature review and citations should be used.

**Response:** We have revised the discussion section substantially. Certain key actionable suggestions were provided under the conclusion section. These are now shifted to the discussion section. Also, we have added India-specific literature while comparing our findings of awareness of schemes. Please refer to line number 379-381. We have also added literature on waiting time and satisfaction. Please refer to line number 397-398. Additionally, a comparison around OOP has already existed in the manuscript. Please refer to line number 404-406. The limitations of this work are added in the discussion section. Please refer to line number 438-443.

**Specific comment 8:** Conclusion: Should be succinct and single or maximum of two paragraphs.

**Response:** We have revised the conclusion section substantially. After shifting certain key actionable suggestions to the discussion section, the conclusion section is now of two paragraphs only.

**Specific comment 9:** Figure 1- 4 are repetition and can be removed.

**Response:** Agreeing to the suggestion, we have removed these figures, as the details are provided in the text. These are now provided as supplementary material.

**Reviewer #2 comments:**

**Specific comment** 1. The paper may give some context of health care utilization and expenditure about the states chosen

Response: We have now added this information, along with a Table. Please refer to line number 148-49 and Table 1 for these details.

**Specific comment** 2.Need to strengthen the discussion section with some comparison of the findings of PM-JAY with private health insurance schemes to judge the strengths and shortcomings of PM-JAY.

**Response:** We couldn’t find relevant research on responsiveness and financial protection from the perspective of beneficiaries of private health insurance schemes. Thus, we could not add such a comparison of PMJAY with private health insurance schemes. However, we have compared our findings with other GSHIS. Please refer to line number 406-420 for these details.

**Specific comment** 3. Quantitative analysis may be augmented by estimating odds-ratios for the chosen indicators.

**Response:** The study did not analyze the associations or determinants of responsiveness (measured in terms of patient satisfaction) and financial protection (measured in terms of incidence and magnitude of OOP). Estimation of odds ratio was therefore not attempted as it was not related to the study objectives. Additionally, small sample size was a limitation of the study restricting the scope of statistical analysis. We, however, explored the subgroup difference in responsiveness and financial protection across socio-economic categories of the respondents. These details are presented in Table 1 as supplementary material.

**Specific comment** 4. The word "improving" may be dropped from the title.

**Response:** Agreeing to the suggestion, we have removed the word ‘improving’ from the title.

**Specific comment** 5. There are many grammatical errors and many sentences need editing to have a more terse presentation.

**Response:** We have revised the manuscript substantially with the help of a professional editor. This has reduced grammatical errors and improved the content.

**Specific comment** 6. In line 251, the figures in the Table and in the description do not match.

**Response:** These were because the findings were presented to highlight the proportion of the respondents who did not make any OOP payments. We have now revised the language and figures to reflect the information in terms of those who made the OOP payment, as presented in the table

**Specific comment** 7. Line 274, and other lines, the style of writing figures is not uniform.

**Response:** We have corrected the style of writing figures to make them uniform at all places.

**Specific comments in the attached document:**

**Comment	1:** check grammar.

**Response:** We have revised the abstract and edited it for grammatical errors.

**Comment	2:** The abbreviation in title and text should be the same, otherwise need to repeat the full form here as well.

**Response:** We have revised the abbreviation in the title.

**Comment	3:** Indicate dollar values also

**Response:** We have revised the abstract and added dollar values.

**Comment	4:** delete. Repeated

**Response:** We have revised the abstract accordingly.

**Comment	5:** Avoid abbreviation

**Response:** We have revised the abstract accordingly.

**Comment	6:** The conclusion seems more like objective and does not follow from results.

**Response:** We have revised the conclusion part of the abstract accordingly.

**Comment	7:** Very long introduction. Clearly provide the background in the context of the objective of the study.

**Response:** We have revised the introduction substantially. The introduction is also shortened by more than fifteen lines.

**Comment	8:** Please place references as per the plos one style - different places before and after full stops.

**Response:** We have revised the references as per PLOS style. They are now after full stops at all places.

**Comment	9:** check grammar.

**Response:** We have revised the entire document for grammatical errors.

**Comment	10:** Major features to be more succinct and summarized in a box under headings

**Response:** We have revised the introduction of PM-JAY substantially and now it is shorter, and in the flow of the text. At this stage, it need not be in the box. However, if the reviewer feels appropriate, we would be happy to put the details in the box.

**Comment	11:** Please provide full references with links and dates accessed as per PLOS style.

**Response:** We have revised this reference. We have used the ‘PLOS’ style in the EndNote referencing software for generating full references for this manuscript.

**Comment	12:** what are autonomous hospitals

**Response:** These are publicly funded hospitals that have special status through legislation and thus, are governed independently. However, since this was not relevant for the objectives, we have removed this sentence.

**Comment	13:** what if the claim os from the same episode of ailment.

**Response:** All patients with single claims were retained in the population list. Few patients required multiple sessions of treatment like dialysis and chemotherapy. In such cases, each session was claimed separately, and thus, there were multiple claims of those patients. We retained all these patients.

**Comment	14:** Would this not add a bias for responsiveness, as the hospitals with least claims can also be the ones where people did not want to go.

**Response:** The hospitals for the study were selected by the state authorities. It was important to ensure the representation of patients across hospitals and specialties within the hospitals. The use of multi-stage sampling method with the use of Probability Proportional to Size ensured that the selection of respondents was not biased either in favour or against any type of specialty and hospital. This information is now explained in the manuscript. Please refer to line number 169-171.

**Comment	14:** They would already have better knowledge of the health scheme, so their responsiveness may be better.

**Response:** The selection of the state with previous experience of implementing a GSHIS was an element of study at the time of selection of the study location. The responsiveness in terms of their registration in the scheme was not analyzed and therefore, ‘n’ for the beneficiary registration did not include these patients. The responsiveness of their hospitalization experience was measured only for the particular episode during the study duration. Thus, such possibility of their experience influencing the knowledge and responsiveness of the scheme may be ruled out.

**Comment	15:** Can be added as supplementary material

**Response:** We have submitted a copy of the questionnaire as supplementary material.

**Comment	15:** Would this not create seasonal bias

**Response:** The study population includes patients who availed hospitalization under PMJAY during April 2019. This was given by the state authorities, and thus, was not under the control of the researchers. The data collection was done during May-June 2019. There could be seasonal effects on the type of illnesses, but the responsiveness and financial protection may not get affected by the time of the year.

**Comment	16:** Can say informed consent was taken

**Response:** We have revised the statement and provided more details in the manuscript. Please refer to line number 190-194.

**Comment	17:** These can be summarized under different headings in a box - in the same format as the results are presented. Some of it also repeated from above. Need to add them together.

**Response:** These are now well-defined and discussed in the methodology section between lines 196-211. We have removed the repetition. The results of this study were documented using the flow of processes in the standard official guideline as a template. The findings are therefore divided into subsections that correspond to the PM-JAY guidelines for hospital-based transactions. These sub-sections are beneficiary identification and registration, treatment package selection and blocking, discharge and payments, and information exchange.

**Comment	18:** Is anything covered before and after hospitalization. Was information collected on experience before and after hospitalization? If referral etc. had the influence on registration process. What about coverage after hospitalization.

**Response:** The study collected information on pre-and post-hospitalization expenses in detail. The analysis of OOP payment includes these details in light of the provision of such coverage under the scheme. The information exchange is an in-built element regarding a) registration done before or at the time of the hospitalization, b) package selection during hospitalization, and c) calls for feedback after the hospitalization. These were studied in detail.

**Comment	19:** why is education of beneficiary/ or highest education in HH not taken as indicator, as awareness and responsiveness depends quite a bit on that.

**Response:** We have now added this information in Table 3.

**Comment	20:** mismatch - 61 vs 74. Also if they were already enrolled in a scheme, one would expect better experience about the process. Could it be they did not want to switch.

**Response:** This is not a mismatch. 74% of the total 61 respondents (i.e. 45 respondents) indicated that they knew about the scheme from the letter. The remaining 39 participants at the time of the research were yet to be transferred from the MA scheme to PMJAY. For all practical purposes, all beneficiaries, irrespective of the scheme received the same coverage for their hospitalization. We did not include these 39 participants for their registration experience as they were not registered in PMJAY till April 2019. The participants did not have choice in switching as the existing scheme was getting subsumed under PMJAY over time.

**Comment	21:** what was the difference in public vs. private will be useful to know

**Response:** The satisfaction was higher among beneficiaries from public sector hospitals (83%) than their counterparts from the private sector (77%). This is now added in the result section. Please refer to line number 296 and 297.

**Comment	22:** You mention preauthorization above also. These are longer than for admission and preauthorization. Do you mean only admission above? Otherwise hours should be more above.

**Response:** The first mention of pre-authorization is in the context of ‘admission and pre-authorization request’ – i.e. time that the AM took in preparing and submitting the request. The second mention is about ‘pre-authorization approval’ to be received from the scheme authorities. The first one is the efficiency of the insurance processes at the level of healthcare providers, and the latter is about the efficiency of the scheme managers at the level of the insurance company / Trust. These details are clarified and streamlined in the subsection of ‘treatment package selection and blocking’ in the result section.

**Comment	23:** Why is the sum of total smaller than public plus private

**Response:** The total of ‘n’ is 100 (after adding 56 private and 44 public). The incidence of OOP is in proportion. So, the total need not be the summation of the two proportions. It is the mean of the two (i.e. 13 and 9).

**Comment	24:** Does PMJAY not cover the medicine bills for medicines not available. Also for a procedure, would everything not be covered?

**Response:** Ideally, all expenses should be covered. According to the guideline, the patient need not pay and the hospital must not do balance billing. However, the finding of this study, therefore, brings evidence on OOP and connects it to the reasons for such OOP in the discussion section. We, therefore, raise the need to address underlying supply-side reasons for OOP.

**Comment	25:** Are they informed and consulted about it before the registration.

**Response:** Yes. In most cases, the patients were informed beforehand about the possibility of such part payments. This was ascertained during the survey through informal discussions with the respondents.

**Comment	26:** Is insurance agency or the scheme informed about these payments charged to the patients?

**Response:** The survey respondents did not know of such communication. Therefore, this is not part of our analysis. However, since part payment is not allowed under the scheme, there is no mechanism/ mandate for the hospitals to make this information available to the insurer/scheme managers.

**Comment	27:** This is really high. Can this be compared with OOPS data from HH survey.

**Response:** This is high in the case of patients who availed of hospitalization in a private hospital in Madhya Pradesh, a state which saw such GSHIS with 5 lac coverage for the first time. Therefore, this needs to be seen contextually for the GSHISs covering secondary and tertiary hospitalization. We refrained from comparing the magnitude of OOP of this study (which was measured for those who made some payment) with similar data from the HH survey as the study population is not comparable. Our study population comprises those who exclusively opted for secondary and tertiary hospitalization, and thus are not comparable with households incurring expenses on a range of healthcare, including primary care and outpatient treatment. We have compared the incidence of OOP among the insured patients with similar research on beneficiaries of past GSHIS like RSBY that offered relatively low coverage. Please refer to line number 406-420 for these details.

**Comment	28:** This should and most of it is part of introduction. Not required here.

**Response:** We have revised this paragraph and removed irrelevant sentences from here.

**Comment	29:** What about the links to the socio economic conditions of the households. Also does education matter.

**Response:** The study did not analyze the determinants of responsiveness (measured in terms of patient satisfaction) and financial protection (measured in terms of incidence and magnitude of OOP). However, on comparing these parameters across the socio-economic subgroups of the respondents, it was found that the satisfaction for registration (91%) and hospitalization (82%) was highest among the illiterate respondents. This sub-group has the lowest incidence of OOP (15%), but reported the highest amount of OOP payment (Rs. 41075). We have provided this information with a table as supplementary material.

**Comment	30:** What about the links to the rights and information. Also not discussed clearly about the payments and links to Aarogya Mitra. Should AM not play a role in helping the patients to keep their payments low. Even if AM desk is not there in hospitals, should AM not visit the hospitalized patient under the scheme. That would improve the quality and financial protection.

**Response:** Aarogya Mitra is a staff of the hospital appointed to streamline the liaison between patients, doctors, and scheme authorities. As an employee of the hospital, the AM interacts with the patients on behalf of the hospitals – at the helpdesk or bedside. The AM’s interaction with the patient is limited to information and documentation for preauthorization and discharge. As for the payment, the AM merely conveys the decision of ‘balance payment’, if any, taken by the hospital authorities. Lastly, being a household survey of the patients, this research did not dwell deeper in the supply side issues of how AM can play an improved role at the provider level.

**Comment	31:** how do this and some other conclusions follow from your results. Please restrict discussion and conclusions to what your results show.

**Response:** This is connected to the reasons that patients indicated for their OOP, especially in the case of those who made a lump sum payment. These patients reported that they were informed at the helpdesk that the actual costs of their treatments or procedures were higher than the amount the hospital would receive from claim reimbursements from PM-JAY; thus, patients needed to pay the balance. This is indicated in the result section (Please refer to line number 32-335). We have revised the language and added corresponding arguments and evidence (Please refer to line number 414-416).
