# Supplementary material for: Experiences and challenges in accessing hospitalization in a government-funded health insurance scheme: Evidence from early implementation of Pradhan Mantri Jan Aarogya Yojana (PM-JAY) in India
Source: PLoS One. 2022 May 12;17(5):e0266798. doi: 10.1371/journal.pone.0266798 (PMC9098065; doi:10.1371/journal.pone.0266798)
Supplement: Attachment — Submitted filename: Responses to comments.docx [file pone.0266798.s006.docx]

- Comment: The title has responsiveness and financial protection. please discuss these clearly.

Response: The meaning of responsiveness and its dimensions are discussed on line number 72 – 82. The details of responsiveness and financial protection used in the study are discussed from line number 138-144, and indicators of responsiveness and financial protection are detailed in line number 211 – 227. We have revised the title to “Experiences and challenges in accessing hospitalization in a Government-Funded Health Insurance Scheme: Evidence from Early Implementation of Pradhan Mantri Jan Aarogya Yojana (PM-JAY) in India”.

- Comment: Abstract is still very vague. Please rewrite the abstract clearly

Response: We have revised the abstract to have objectives linked to the title of the study, methods stating the way indicators of responsiveness and financial protection were defined, and conclusion following from the results. Please refer line number 27 – 61.

- Comment: Introduction: Line 95 – not clear - 500 million beneficiaries from the 100 million poor and vulnerable families.

Response: We have revised this. Please refer line 90 – 96.

- Comment: Introduction could clearly state how responsiveness and financial protection would be studied.

Response: We have added the details. In line 72-82 the meaning of responsiveness and its dimensions are discussed. Towards the end of the Introduction section, in line 138 -144 further details of the way this study attempts to study responsiveness and financial protection have been specified. In the Methods section, the indicators of responsiveness and financial protection used in the study are discussed from line number 211 – 227.

- Comment: Table 1: the data should not be a part of the methodology. Even if is based on secondary sources, these are results. However not being a part of the main study, these could be supplementary material and the figures can be used in the context of responsiveness and financial protection in discussion. The hospital OOP data can be found in the latest version of the NSSO key indicators for 2017-18. Plus, the OOP differs when hospitalization is in public or private facilities and in rural and urban areas. The NHA data is also dated and available for more recent years

Response: We have updated the table with the latest data. We have also taken the table to supplementary material. Please refer to Supplementary Table 1.

- Comment: Was data collection completed within one month.

Response: No. The sample was derived from the patients who availed treatment during this one month. The study duration was March – August 2019. We have worded the description accordingly. Please refer line number 154, and 169.

- Comment: Methods- Still; very weak to compare the two states. No clear statistical analysis to understand the differences in the two states. Just presenting table 1 in methods does not show how the results are compared across two states and why the differences are observed. It’s just data presented from a survey with no good analysis. The paper lack rigorous statistical analysis. No statistical tools were applied to explain differences in two states or controlling for hospital types.

Response: We have revised the methods sections. We have used Non-parametric test (Mann–Whitney U test) to compare beneficiaries’ responses across states (Gujarat and Madhya Pradesh), hospitals across states (public and private hospitals), hospitals within a state (public and private hospital in Gujarat, public and private hospital in Madhya Pradesh), and across socio-economic categories. We have also carried out cumulative odds ordinal logistic regression with proportional odds to determine the effect of state, hospital type, OOP incidence, beneficiary’s caste, beneficiary’s location, beneficiary level of education, type of care/services received, help received at PM-JAY registration desk, and the number of days for which the beneficiary was admitted in a hospital (independent variables), on the beneficiary’s satisfaction with the experience of hospitalization under PMJAY (dependent variable). Please refer line numbers 233-272. Please refer to the results section for the results of the Non-parametric test (Mann–Whitney U test) and cumulative odds ordinal logistic regression with proportional odds. Please refer to the results section for the description of results obtained.

- Comment: Results- Why are the n in tables 4 and 5 different. Tables 4 and 5 can be combined.

Response: Table 4 was about the incidence of OOP and thus ‘n’ refers to those who made such expenses. Table 5 was about the magnitude of OOP payment for those who experienced OOP. We have combined Tables 4 and 5. Please refer to Table 3 on line number 422.

- Comment: No Differences between two states were tested by each socio-economic conditions

Response: We have used Non-parametric test (Mann–Whitney U test) to test the differences by each socio-economic condition. Please refer line number 445 – 450. We have also used caste, level of education, location of residence as independent variables in cumulative odds ordinal logistic regression with proportional odds. Please refer line 246-272, refer to table 4 and 5 at line 471 and 482.

- Comment: The discussion section is very weak and does not clearly discuss the results of the study. None of the points seem to cater to the international audience and are very broad even for Indian perspective

Response: We have revised the discussion section to highlight the significance of the results. We have also compared the findings of this study with other studies carried out in national and international contexts. We have also discussed reasons for difference in performance across states and types of hospitals. Please refer to line from 500 – 637.

- Comment: Lines 383, can you pls compare with the references mentioned in quantitative terms to provide a perspective of improvement in providing the information to patients.

Response: Information provided. Please refer to line 511 – 512.

- Comment: OOP discussion should be in perspective with non-insured patients and also insured in other programs.

Response: We have updated the discussion section. In the discussion section, results related to OOP payment are discussed in comparison with the OOP payment made by non-insured, those insured under PM-JAY in other states of India. Please refer line number 568 – 588.

- Comment: Neither objectives nor main outcome measurements were clearly defined in the paper. The conclusion seems don't appear from the findings of the survey.

Response: The objectives and aims of the study, outcome measures are outlined in the introduction section. Please refer line 130 – 144. The details of the way outcome measures were operationalized and data was collected are provided in line number 208-228. We have thoroughly revised the results, discussion, and conclusion sections of the paper.

- Comment: There is still a lot of repetition in conclusions and there needs to be a para highlighting important points.

Response: We have revised the conclusion part to highlight important takeaways. Please refer to lines 639-663.

- Comment: It appears sample is biased when we talk about the share of SC & ST patients in Madhya Pradesh.

Response: The profile of survey respondents as provided in table 2 on line 292 was a finding of the study. The sample of respondents for the study was drawn from the list of claims provided by the state health agency of the respective states. These were patients who received treatment from sample hospitals during April 2019. Scheduled Tribe, Scheduled Caste, Other Backward Caste represent the marginalized section of society in India. Taken together beneficiaries from these castes constitute 74% of the sample of beneficiaries from Madhya Pradesh.

- Comment: The major flaw is in the sample selection procedure where they just did quota sampling from two tales of the patients hospitalized during April 2019 in 14 hospitals. One can't portray a robust picture using the top 10% and bottom 10% of patients using services as "Highest"/ "Lowest" consumption of health care resources in the sampled hospitals. Instead of following a randomization process, they eliminated the middle 80% of cases.

Response: The sample of this study did not eliminate 80% of cases. We applied the ‘top-bottom’ rule` to the specialties, and not to patients, at each of the 14 sample hospitals for which we received the claims data from the insurance authorities. The hospitals included in this study were single- and multi-speciality in nature, and thus, we decided to include a representation of patients across a range of services, by selecting clusters having top 10% (most popular services) and bottom 10% loads of patients (Least popular services) from each hospital. These are not 10% patients, but clusters. By following this procedure, we had a pool of 57% of patients (67% in Gujarat and 53% in Madhya Pradesh) from which we randomly selected the respondents. There is a possibility that this way of selecting the respondents may have introduced some bias. We have acknowledged this a possibility in the discussion section of the paper. Please refer to lines 626-633.

- Comment: I have mentioned comments in the body of the paper and on tables; e.g. I can't understand they are using "Total" heading in both columns and rows even whilst presenting statistics on average for two states.

Response: We have revised the paper to take care of the comments received in the body of paper.
